# Single-cell integration reveals metaplasia in inflammatory gut diseases

**DOI:** 10.1038/s41586-024-07571-1

**Published:** 2024-11-20

**Authors:** Amanda J. Oliver, Ni Huang, Raquel Bartolome-Casado, Ruoyan Li, Simon Koplev, Hogne R. Nilsen, Madelyn Moy, Batuhan Cakir, Krzysztof Polanski, Victoria Gudiño, Elisa Melón-Ardanaz, Dinithi Sumanaweera, Daniel Dimitrov, Lisa Marie Milchsack, Michael E. B. FitzPatrick, Nicholas M. Provine, Jacqueline M. Boccacino, Emma Dann, Alexander V. Predeus, Ken To, Martin Prete, Jonathan A. Chapman, Andrea C. Masi, Emily Stephenson, Justin Engelbert, Sebastian Lobentanzer, Shani Perera, Laura Richardson, Rakeshlal Kapuge, Anna Wilbrey-Clark, Claudia I. Semprich, Sophie Ellams, Catherine Tudor, Philomeena Joseph, Alba Garrido-Trigo, Ana M. Corraliza, Thomas R. W. Oliver, C. Elizabeth Hook, Kylie R. James, Krishnaa T. Mahbubani, Kourosh Saeb-Parsy, Matthias Zilbauer, Julio Saez-Rodriguez, Marte Lie Høivik, Espen S. Bækkevold, Christopher J. Stewart, Janet E. Berrington, Kerstin B. Meyer, Paul Klenerman, Azucena Salas, Muzlifah Haniffa, Frode L. Jahnsen, Rasa Elmentaite, Sarah A. Teichmann

**Affiliations:** 1 Wellcome Sanger Institute, Wellcome Genome Campus, Cambridge, UK; 2 Department of Pathology, University of Oslo and Oslo University Hospital–Rikshospitalet, Oslo, Norway; 3 Department of Systems Biology, The University of Texas MD Anderson Cancer Center, Houston, US; 4 Inflammatory Bowel Disease Unit, Institut d’Investigacions Biomèdiques August Pi I Sunyer (IDIBAPS), Hospital Clínic, Barcelona, Spain; 5 Centro de Investigación Biomédica en Red de Enfermedades Hepáticas y Digestivas (CIBEREHD), Barcelona, Spain; 6 Institute for Computational Biomedicine, Heidelberg University, Faculty of Medicine, Heidelberg University Hospital, Bioquant, Heidelberg, Germany; 7 Translational Gastroenterology and Liver Unit, Nuffield Department of Medicine, University of Oxford, Oxford, UK; 8 Translational and Clinical Research Institute, Newcastle University, Newcastle, UK; 9 Department of Histopathology and Cytology, Cambridge University Hospitals, Cambridge, UK; 10 Department of Pathology, University of Cambridge, Cambridge, UK; 11 Translational Genomics, Garvan Institute of Medical Research, Sydney, New South Wales Australia; 12 School of Biomedical Sciences, University of New South Wales, Sydney, New South Wales Australia; 13 Department of Surgery, University of Cambridge, Cambridge, UK; 14 Cambridge Biorepository for Translational Medicine, Cambridge NIHR Biomedical Research Centre, Cambridge, UK; 15 Department of Haematology, Cambridge Stem Cell Institute, Cambridge, UK; 16 Cambridge Stem Cell Institute, University of Cambridge, Cambridge, UK; 17 University Department of Paediatrics, University of Cambridge, Cambridge, UK; 18 Department of Paediatric Gastroenterology, Hepatology and Nutrition, Cambridge University Hospitals, Cambridge, UK; 19 Department of Gastroenterology, Oslo University Hospital, Oslo, Norway; 20 Institute of Clinical Medicine, University of Oslo, Oslo, Norway; 21 Peter Medawar Building for Pathogen Research, Nuffield Department of Clinical Medicine, University of Oxford, Oxford, UK; 22 NIHR Oxford Biomedical Research Centre, Oxford University Hospitals NHS Foundation Trust, Oxford, UK; 23 Biosciences Institute, Newcastle University, Newcastle upon Tyne, UK; 24 Department of Dermatology and National Institute for Health Research (NIHR) Newcastle Biomedical Research Centre, Newcastle upon Tyne Hospitals NHS Foundation Trust, Newcastle upon Tyne, UK; 25 Ensocell Therapeutics, BioData Innovation Centre, Wellcome Genome Campus, Cambridge, UK; 26 Theory of Condensed Matter, Cavendish Laboratory/Department of Physics, University of Cambridge, Cambridge, UK; 27 Department of Medicine, University of Cambridge, Cambridge, UK; 28 CIFAR Macmillan Multi-scale Human Program, CIFAR, Toronto, Ontario Canada

**Keywords:** Cell biology, Immunology, Gastrointestinal diseases, Transcriptomics, Data integration

## Abstract

The gastrointestinal tract is a multi-organ system crucial for efficient nutrient uptake and barrier immunity. Advances in genomics and a surge in gastrointestinal diseases^1,2^ has fuelled efforts to catalogue cells constituting gastrointestinal tissues in health and disease^3^. Here we present systematic integration of 25 single-cell RNA sequencing datasets spanning the entire healthy gastrointestinal tract in development and in adulthood. We uniformly processed 385 samples from 189 healthy controls using a newly developed automated quality control approach (scAutoQC), leading to a healthy reference atlas with approximately 1.1 million cells and 136 fine-grained cell states. We anchor 12 gastrointestinal disease datasets spanning gastrointestinal cancers, coeliac disease, ulcerative colitis and Crohn’s disease to this reference. Utilizing this 1.6 million cell resource (gutcellatlas.org), we discover epithelial cell metaplasia originating from stem cells in intestinal inflammatory diseases with transcriptional similarity to cells found in pyloric and Brunner’s glands. Although previously linked to mucosal healing^4^, we now implicate pyloric gland metaplastic cells in inflammation through recruitment of immune cells including T cells and neutrophils. Overall, we describe inflammation-induced changes in stem cells that alter mucosal tissue architecture and promote further inflammation, a concept applicable to other tissues and diseases.

## Abstract (web summary)

The study provides a comprehensive transcriptomic atlas of the human gastrointestinal tract across the lifespan, highlighting inflammation-induced changes in epithelial stem cells that alter mucosal architecture and promote further inflammation.

## Main

The human gastrointestinal tract is a complex system comprising several organs that work together to absorb nutrients while simultaneously providing an immunologically active barrier. Diseases of the gastrointestinal tract are prevalent: ulcerative colitis and Crohn’s disease affect over 7 million people worldwide, and 2 million new colorectal cancer (CRC) cases are diagnosed annually^1,2^. Single-cell transcriptomics has offered unprecedented molecular insights of gastrointestinal homeostasis, development and disease^5–9^. Over 25 single-cell RNA sequencing (scRNA-seq) studies of the human gastrointestinal tract have been published to date, primarily focused on specific organs and/or cell types. The integration of these publicly available datasets provides a valuable resource for the Human Cell Atlas community and beyond^3^, and enables cross-regional comparisons of gastrointestinal cell types.

The epithelial cells lining the gastrointestinal tract lumen arise from a common endoderm progenitor and acquire their regional identity early in embryogenesis^10^. This regional identity can be altered in adulthood leading to metaplasia, where mature tissue is replaced by cells normally occurring in other anatomical regions^4^. Intestinal metaplasia is well described in the stomach and in patients with Barrett’s oesophagus where the mucosa is transformed to intestinal epithelial cells, increasing the risk of gastric and oesophageal adenocarcinomas^11,12^. Conversely, pyloric metaplasia of intestinal tissue, comprising cells expressing *MUC6* and *MUC5AC*^4^, is less well characterized (also known as pseudopyloric metaplasia, gastric metaplasia, ulcer-associated cell lineage and spasmolytic polypeptide-expressing metaplasia). Histological studies^4,13,14^ have suggested that pyloric metaplasia may arise as part of the mucosal healing process and can transition to neoplasia^4^. However, the origin and functional role of metaplastic cells in acute and chronic tissue damage remain unresolved.

In this study, we created a gastrointestinal tract atlas by integrating published and newly generated scRNA-seq data spanning health and disease. Utilizing this resource (gutcellatlas.org) of 1.6 million cells across 271 donors, we examined cell types and signatures in inflammatory intestinal diseases. We identified *MUC6*^+^ metaplastic cells from inflamed intestines from patients with inflammatory bowel disease (IBD) and coeliac disease, uncovering the full transcriptome of pyloric gland metaplastic cells, which we termed inflammatory epithelial cells (INFLAREs). We propose that a shift in the epithelial stem cell state alters the differentiation pathway from healthy to metaplastic lineages, which in turn contribute to ongoing inflammation in chronic disease.

## Pan-gastrointestinal data integration

**Fig. 1:**
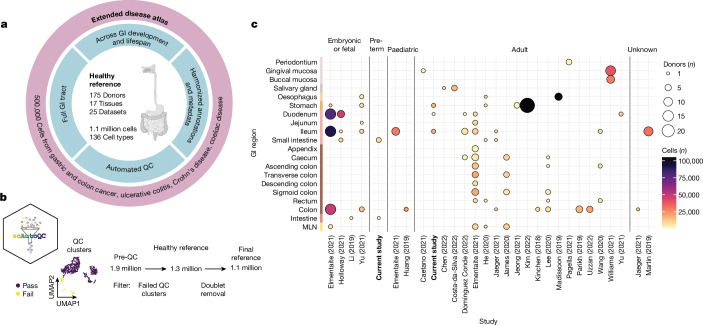
Overview of pan-gastrointestinal cell integration. **a**, Schematic overview of the atlas denoting the healthy reference as a core, with additional disease datasets mapped by transfer learning. GI, gastrointestinal; QC, quality control. Schematic in panel **a** was created with BioRender (https://biorender.com). **b**, Overview of scAutoQC, an automated, unsupervised quality control approach to remove low-quality cells. UMAP, uniform manifold approximation and projection. **c**, Overview of the number of cells and donors per study, broken down by age and region of the gastrointestinal tract (*y* axis). The dot size indicates the number of donors, and the colour indicates the number of cells. The colours of the *y* axis indicate broad-level organs (oral mucosa, salivary gland, oesophagus, stomach, small intestine, large intestine and mesenteric lymph node (MLN)). Caetano (2021), ref. ^50^; Chen (2022); ref. ^51^; Costa-da-Silva (2022), ref. ^52^; Domínguez Conde (2022), ref. ^53^; Elmentaite (2021), ref. ^5^; He (2020), ref. ^54^; Holloway (2021), ref. ^55^; Huang (2019), ref. ^56^; Jaeger (2021), ref. ^57^; James (2020), ref. ^58^; Jeong (2021), ref. ^59^; Kim (2022), ref. ^60^; Kinchen (2018), ref. ^9^; Lee (2020), ref. ^61^; Li (2019), ref. ^62^; Madissoon (2019), ref. ^63^; Martin (2019), ref. ^6^; Pagella (2021), ref. ^64^; Parikh (2019), ref. ^23^; Uzzan (2022), ref. ^65^; Wang (2020), ref. ^66^; Williams (2021), ref. ^19^; Yu (2021), ref. ^67^.

**Extended Data Fig. 1:**
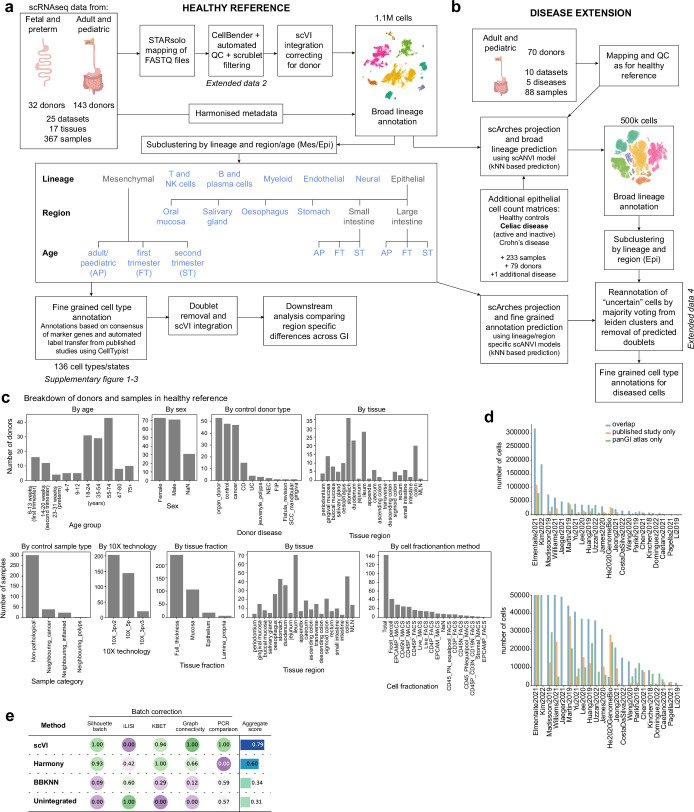
Overview of atlas assembly. a) Detailed flowchart of the methods used to assemble the healthy reference, datasets were remapped and filtered based on scAutoQC automated QC pipeline (Supplementary Fig. 2), integrated with scVI and annotated as broad lineages. Broad lineages were subclustered, and lineages with high level of heterogeneity (Epithelial and Mesenchymal lineages) were further subclustered based on age and/or region to accurately annotate at a fine-grained level. Cells in these subclustered views of the healthy reference were annotated by a semi-automated approach, taking into account the marker genes and CellTypist predictions from published studies. Schematic in panel **a** was created with BioRender (https://biorender.com). b) The healthy reference was used as an anchor to project disease datasets onto the atlas using scArches, fine-grained annotations were generated in a two-step approach, first with broad lineage prediction using scANVI and subclustering by lineage/region as with the healthy reference to predict the fine-grained annotations. Most disease data was remapped and QC’ed as with the healthy reference, except two additional studies from CD (Kong, 2023) and celiac disease (M.E.B.F., unpublished) which were added to the atlas from the published count matrices. c) Breakdown of the distribution of donors and samples in the healthy reference based on various metadata as specified. d) Overlapping and unique cells in our pan-GI atlas and the published studies (based on available count matrices). e) Benchmarking of batch correction across 3 integration methods for the healthy reference atlas versus the unintegrated atlas.

**Extended Data Fig. 2:**
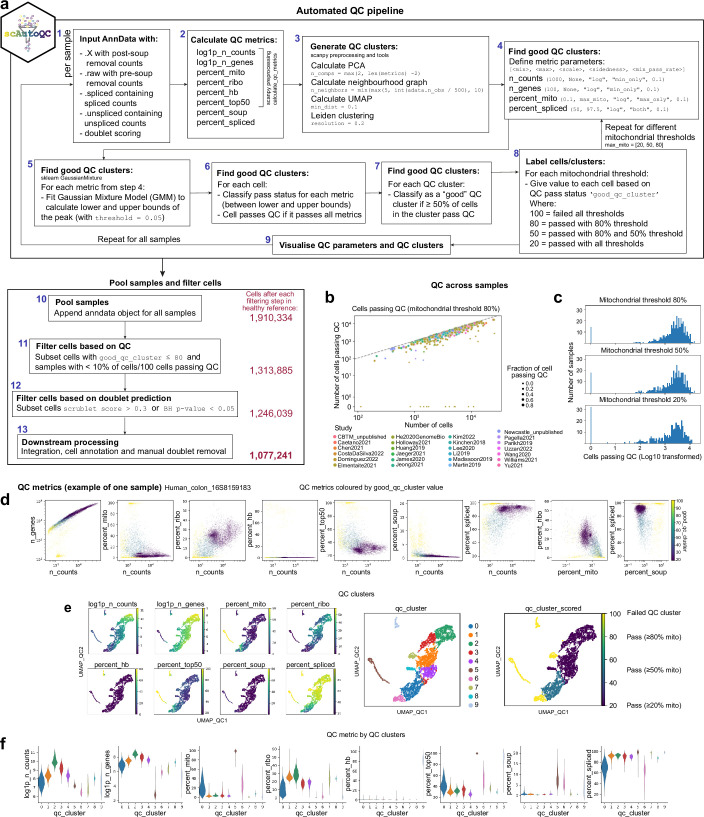
Overview of scAutoQC method. a) Summary of the automated QC pipeline. Standard QC metrics are calculated and dimensions of 8 QC metrics (listed in step 2) are reduced, neighbours calculated and UMAP generated. Clusters from this UMAP are classified as “good” if ≥ 50% fall within upper and lower bounds (calculated by Gaussian Mixture Model) of 4 QC metrics (listed in step 4). Step 4–7 was repeated for 3 different mitochondrial thresholds (20%, 50%, 80%) and all steps were repeated for all samples. Finally samples are pooled, and cells within clusters that failed automated QC when mitochondrial threshold is 80%, and predicted as doublets (based on scrublet score calculated on a per sample basis) are removed before downstream processing. b) Plot of cells passing QC vs number of cells per sample across studies. Dotted line represents threshold for 100% of cells/sample passing QC. c) Histogram showing distribution of cells passing QC (log base 10) across the 3 mitochondrial thresholds. d-f) Example QC plots from one sample where d) is showing QC distribution of QC metrics where each data point is a cell, coloured by good_qc_cluster value (see step 8 of panel a). e) shows the QC UMAPs with the 8 QC metrics (listed in step 2 panel a), QC leiden clusters and good_qc_cluster value (see step 8 of panel a). f) violin plot of the 8 QC metrics (listed in step 2 of panel a) for each QC leiden cluster. In this sample for example, cluster 5 has failed QC because cells in this cluster have high % of mitochondrial reads, low genes and high percentage of genes expressed within the top 50 genes.

We curated, integrated and harmonized healthy cells across the gastrointestinal tract from 23 published and 2 unpublished scRNA-seq datasets (Fig. 1a–c, Extended Data Fig. 1a,b and Supplementary Table 1). Tissues covered include the oral mucosa, oesophagus, stomach, small and large intestines, and mesenteric lymph nodes. To uniformly process the data, we remapped raw sequencing data and processed gene counts through our newly developed quality control pipeline (scAutoQC), removing low-quality cells in an unbiased and automated way (Methods; Fig. 1b, Extended Data Figs. 1 and 2 and Supplementary Note 1). We used single-cell variational inference (scVI) to integrate the data, which outperformed other methods (Extended Data Fig. 1e).

**Extended Data Fig. 3:**
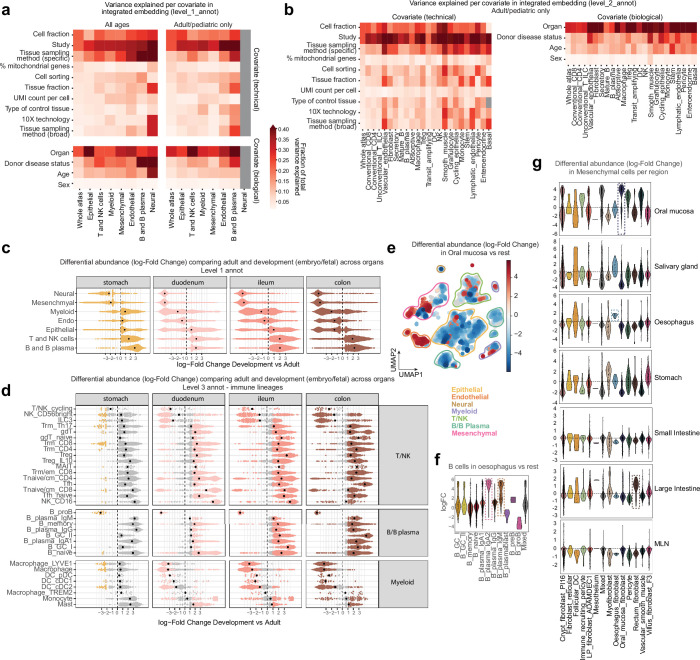
Analysis of cells within the healthy reference. a) Analysis of metadata covariate contribution of variance in the integrated healthy reference embedding per cell type at broad level annotations (level_1_annot). b) Analysis of covariate contribution of variance per cell type at mid-level annotations (level_2_annot). c) Differential abundance analysis (Milopy) comparing broad level cell type (level_1_annot) abundance between adult/pediatric samples and developing samples (embryo, fetal and preterm), broken down by GI region with sufficient data for comparison. Each datapoint is a neighbourhood with positive log-fold change values indicating enrichment of lineage in adult/pediatric GI vs developing GI. d) Differential abundance analysis (Milopy) comparing fine-grained cell type/state (level_3_annot) abundance from immune lineages between adult/pediatric samples and developing samples (embryo, fetal and preterm), broken down by GI region. Each datapoint is a neighbourhood with positive log-fold change values indicating enrichment of cell type/state in adult/pediatric GI vs developing GI. Coloured data points are significantly enriched/depleted neighbourhoods. e) UMAP showing differential abundant neighbourhoods in the healthy reference comparing Oral mucosa to other organs throughout the GI tract in adult/pediatric samples. Positive log-fold change indicates enrichment of neighbourhoods in Oral mucosa. Coloured neighbourhoods show significant enrichment/depletion. f) Violin plot of B and B plasma cells showing enrichment of IgA2 and IgM plasma cells in oesophagus compared to other organs in the atlas. g) Differential abundance of Mesenchymal populations in adult/pediatric samples across each GI region compared to all others combined. Three tissue specific fibroblast populations were annotated, oral mucosa, oesophagus and rectum fibroblasts.

The final integrated data were annotated into seven broad lineages (Extended Data Fig. 1a), subclustered and further annotated into fine-grained cell types (Supplementary Figs. 1–3). Owing to large heterogeneity across gastrointestinal regions and life stages (Extended Data Fig. 3a,b), we further subclustered epithelial and mesenchymal cells by age and/or region, to accurately annotate fine-grained cell types (Extended Data Fig. 1a). Cell types were annotated by a semi-automated method, with manual annotations based on known marker genes cross-referenced with automated annotations based on published studies^5,6,15^ (Methods). In total, our healthy reference atlas comprised approximately 1.1 million cells from 143 adult or paediatric and 32 embryonic, fetal or preterm donors, annotated to 136 fine-grained cell types (Extended Data Fig. 1 and Supplementary Figs. 1–3). We annotated 51 epithelial cell types or states, highlighting commonly occurring and temporally or spatially restricted populations (Supplementary Fig. 2). Our atlas highlighted rare and difficult to distinguish cell types with varying representation across donors, studies and locations (Supplementary Figs. 4 and 5 and Supplementary Note 1). We resolved diverse immune populations including 17 T or natural killer (NK), 16 myeloid and 11 B and B plasma cell subsets (Supplementary Fig. 1).

## Cellular changes in the healthy gastrointestinal tract

Comparing cell-type composition in the developing versus the mature (paediatric and adult) stomach, duodenum, ileum and colon, we observed enrichment of neural and mesenchymal lineages in developing tissues (Extended Data Fig. 3c). Myeloid populations, especially macrophages and LYVE1^+^ macrophages, were also enriched in developing compared with adult small and large intestines (Extended Data Fig. 3c,d). In line with the development of intestinal IgA responses after birth^16^, most B cell subsets were enriched in the mature gastrointestinal tract (Extended Data Fig. 3d). By contrast, progenitor B cells were enriched in developing gastrointestinal tissues, as previously observed^15^ (Extended Data Fig. 3d). Although most T cell populations were enriched in mature gastrointestinal tissues, ILC3 and CD56^bright^ cytotoxic NK cells were enriched in the developing gastrointestinal tissues (Extended Data Fig. 3d).

Differential abundance comparison across mature gastrointestinal regions revealed specific enrichment of endothelial cells in oral mucosa (Extended Data Fig. 3e), consistent with a high level of vascularization^17^. IgA2 and IgM plasma cells were enriched in the oesophagus compared with other tissues (Extended Data Fig. 3f). In mesenchymal populations, several region-specific fibroblasts were enriched in the oral mucosa, oesophagus and rectum (Extended Data Fig. 3g and Supplementary Fig. 1a).

## Disease-relevant cell dynamics in IBD

**Fig. 2:**
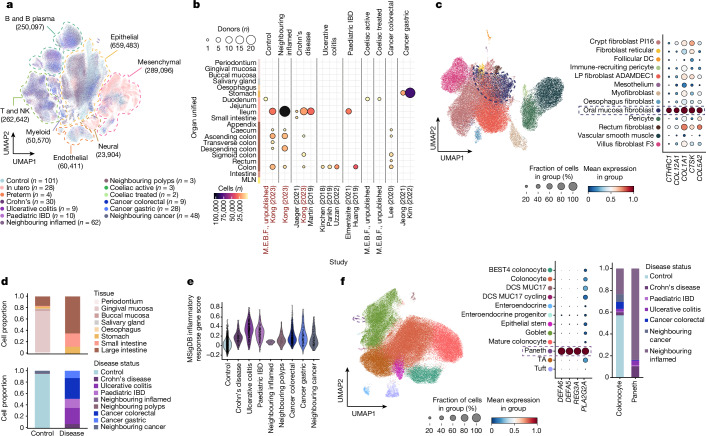
Metaplastic cell lineages in IBD. **a**, UMAP of joint healthy and disease atlas with cells coloured by disease category. *n* refers to the number of donors. The dashed lines indicate broad cell lineages, with cell numbers indicated in parentheses. **b**, Dotplot of extended disease data showing the number of cells (colour) and donors (dot size) per study and disease. Studies in red (M.E.B.F., unpublished and Kong (2023) (ref. ^22^)) were added to the atlas as count matrices. The colours of the *y* axis are the same as Fig. 1c. **c**, UMAP and marker gene dotplot of mesenchymal populations from healthy and diseased adult or paediatric tissue, with ‘oral mucosa fibroblasts’ outlined by dashed lines. DC, dendritic cell; LP, lamina propria. **d**, Barplots with proportions of oral mucosa fibroblasts or inflammatory fibroblasts in control (total *n* = 4,378 cells) and disease (total *n* = 2,403 cells) across gastrointestinal regions. **e**, Violin plot of the MSigDB inflammatory response gene score in oral mucosa or inflammatory fibroblasts across disease categories. The pathway is significant from gene set enrichment analysis comparing differential gene expressions between oral mucosa fibroblasts in healthy versus diseased samples (Extended Data Fig. 4h). **f**, UMAP (left) and marker gene dotplot (middle) of large intestinal epithelial cells from adult or paediatric healthy and diseased samples, highlighting metaplastic Paneth cells (dashed outline). A barplot (right) of cell proportions from control and disease of colonocytes versus Paneth cells is also shown. DCS, deep crypt secretory; TA, transit amplifying.

Next, we projected disease data from patients with ulcerative colitis, Crohn’s disease, paediatric IBD, coeliac disease (unpublished), CRC and gastric cancer onto the healthy reference (Methods; Fig. 2a,b and Supplementary Fig. 6). Overall, we added approximately 500,000 cells to our atlas, totalling 1.6 million cells across 27 studies, 271 donors and 6 gastrointestinal diseases. To annotate disease cells, we projected disease data onto our subclustered, lineage-specific and region-specific views of the atlas (Methods; Extended Data Fig. 1b and Supplementary Figs. 7 and 8).

**Extended Data Fig. 4:**
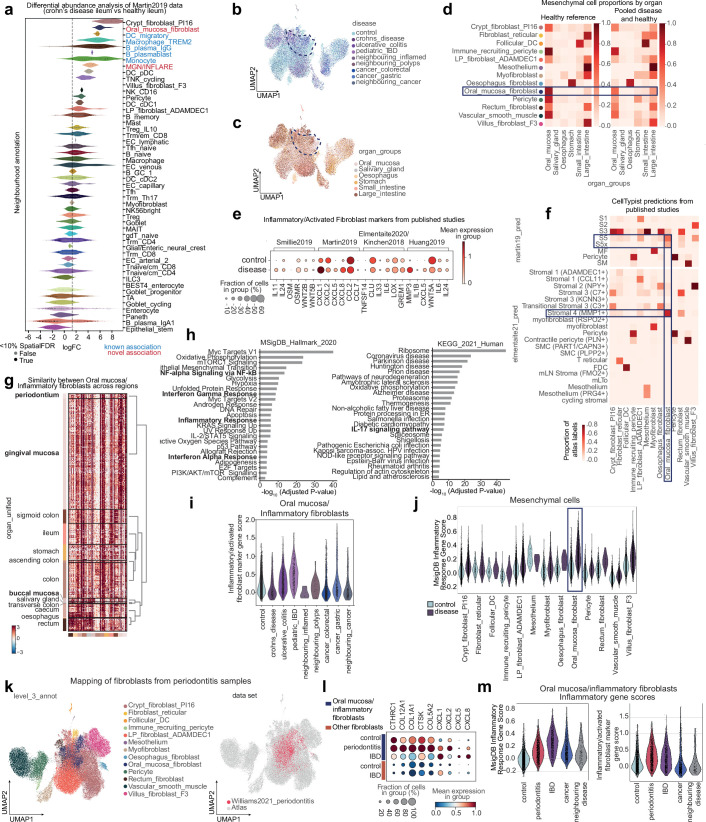
Inflammatory fibroblasts in disease share transcriptional similarity to homeostatic fibroblast population in the oral mucosa. a) Differential abundance analysis of cell neighbourhoods from Martin et al. (2019)^6^ dataset based on embedding on the whole atlas^84^. Cell neighbourhoods with positive log fold change are enriched in CD compared to healthy samples. b) UMAP of mesenchymal cells from adult/pediatric samples in health and disease, shown by disease category. Dashed line highlights the oral mucosa fibroblast cluster. c) UMAP of mesenchymal cells from adult/pediatric samples in health and disease, shown by organ. Dashed line highlights the oral mucosa fibroblast cluster. d) Proportion of mesenchymal cell types/states by organ in the healthy reference and combined healthy and disease. Oral mucosa fibroblasts appear in other organs in disease. e) Markers of inflammatory and activated fibroblasts from published studies^99^ showing expression in oral mucosa/inflammatory fibroblasts from controls (oral mucosa fibroblasts) and disease (inflammatory fibroblasts) samples. f) CellTypist predictions of cell annotations in mesenchymal populations from published studies^5,6^ showing oral mucosa fibroblasts predicted to be inflammatory/activated fibroblast populations in both studies. g) Differential gene expression and hierarchical clustering of oral mucosa/Inflammatory fibroblasts from different regions. Oral mucosa fibroblasts from gingival mucosa and periodontium are most distinct from fibroblasts in other organs. h) Gene set enrichment analysis showing pathways (including various inflammatory pathways) enriched in inflammatory fibroblasts (disease) compared to oral mucosa fibroblasts (healthy). The adjusted p-values have been calculated using wilcoxon rank-sum test. i) Gene score for inflammatory/activated fibroblasts markers in (d) expressed in oral mucosa/inflammatory fibroblasts across disease conditions. j) MSigDB inflammatory response gene score (significantly enriched in inflammatory vs oral mucosa fibroblasts), across all mesenchymal cell types/states in control and disease samples. k) UMAP of mesenchymal populations from the atlas with the addition of fibroblasts from periodontitis data^19^ mapped onto the atlas using scArches and scANVI, coloured by level 3 annotation and highlighting the added data. LP = lamina propria. l) Dotplot showing expression of oral mucosa marker genes and inflammatory chemokines in oral mucosa/inflammatory fibroblasts in healthy tissue, periodontitis and IBD. Expression in other fibroblasts (combined population including crypt_fibroblast_PI16, LP_fibroblast_ADAMDEC1, oesophagus fibroblast, rectum fibroblast and villus_fibroblast_F3) from control and IBD shown for comparison. m) Inflammatory gene scoring in oral mucosa/inflammatory fibroblasts across disease conditions, as in Fig. 2e and Extended Data Fig. 4i,j.

Focusing on IBD, we analysed differences in cell abundance and gene expression programs using unsupervised consensus non-negative matrix factorization (cNMF) and differential gene expression analysis (Methods). These analyses highlighted known cell-type abundance changes in IBD, along with disease-specific gene expression programs across lineages (Extended Data Fig. 4a, Supplementary Fig. 9 and Supplementary Note 2). We observed an enrichment of oral mucosa fibroblasts in Crohn’s disease compared with the healthy ileum (Extended Data Fig. 4a).

Inflammatory fibroblast populations in IBD and cancer have been described^18^ and are expected to map imperfectly onto a healthy reference. In our atlas, disease-specific fibroblasts from IBD and cancer samples from the stomach, and small and large intestines surprisingly mapped to oral mucosa fibroblasts. Thus, disease-specific fibroblasts share transcriptional similarity to healthy fibroblasts in the oral cavity, albeit with upregulated inflammatory gene signatures compared with their healthy counterparts (Fig. 2c–e, Extended Data Fig. 4b–j and Supplementary Note 3). In periodontitis, gingival mucosa fibroblasts similarly upregulate inflammatory genes, particularly those involved in recruiting neutrophils (*CXCL1*, *CXCL2*, *CXCL5* and *CXCL8*) to aid in wound healing^19,20^ (Extended Data Fig. 4k–m). We hypothesize that in the intestines, this inflammatory fibroblast state only arises in severe inflammatory environments similar to inflamed gingival mucosa.

**Extended Data Fig. 5:**
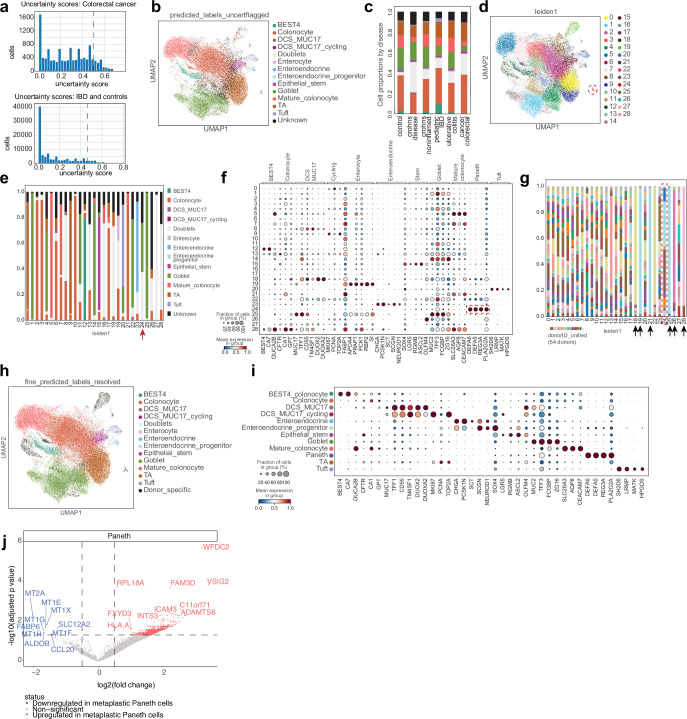
Identification of metaplastic Paneth cells in diseased large intestine. a-i) Example workflow to finalise transferred annotations from scANVI/weighted kNN trainer for large intestine epithelial cells in disease. a) Distribution of uncertainty scores in disease data from large intestine epithelial cells from cancer and non-cancer. Dashed line indicates the 90th percentile cut off, where cells with an uncertainty score above this are classified as “unknown”. b) UMAP of large intestine epithelial cells with predicted annotations and unknown cells flagged. DCS = deep crypt secretory cells. c) Proportions of predicted large intestine epithelial cell annotations (colours as in b) including unknown cells by disease. d) UMAP of large intestine epithelial cells with leiden clustering at resolution = 1, used to reclassify unknown cells based on majority voting. e) Proportions of predicted large intestine epithelial cell annotations by leiden cluster. Red arrow points to cluster 24, which was reannotated to Paneth cells but originally annotated as a combination of goblet cells, doublets and unknown cells. f) Marker gene dot plot of large intestine epithelial cells and Paneth cells by leiden cluster. Paneth cell markers are highlighted for cluster 24. g) Proportions of cells in each leiden by donor. Black arrows highlight clusters dominated by cells from only one donor (excluded from the atlas), and red arrow highlights cluster 24 which contains metaplastic Paneth cells. h) UMAP of reannotated large intestine epithelial cells from disease, including metaplastic Paneth cells. i) Marker gene dot plot for reannotated cell types in large intestine epithelial cells from disease. j) Pseudobulk (decoupler) and differential gene expression analysis (DESeq2) comparing Paneth cells from inflamed small intestine (n = 27) and metaplastic Paneth cells from inflamed large intestine (n = 9). Genes with a positive log2FC are upregulated in metaplastic Paneth cells compared to native small intestine Paneth cells, based on two-sided Wald test with Benjamini and Hochberg correction.

In the epithelial compartment, we observed a distinct disease-specific cluster of cells in the large intestine, which we annotated as Paneth cells based on the marker genes *DEFA5*, *DEFA6*, *REG3A* and *PLA2G2A* (Fig. 2f and Extended Data Fig. 5a–i). Paneth cells were found across inflamed and neighbouring tissue from patients with IBD, but not in the healthy controls, consistent with Paneth cell metaplasia in chronic colon inflammation^21,22^ (Fig. 2f and Extended Data Fig. 5g). Comparing gene expression profiles of native Paneth cells in the inflamed small intestine with metaplastic Paneth cells in the inflamed colon, we identified upregulation of *WFDC2* and *FAM3D* (Extended Data Fig. 5j). These genes are involved in colon homeostasis and controlling bacterial growth, supporting the role for Paneth cell metaplasia in barrier restoration^23,24^.

## Epithelial metaplasia in gut disease

**Fig. 3:**
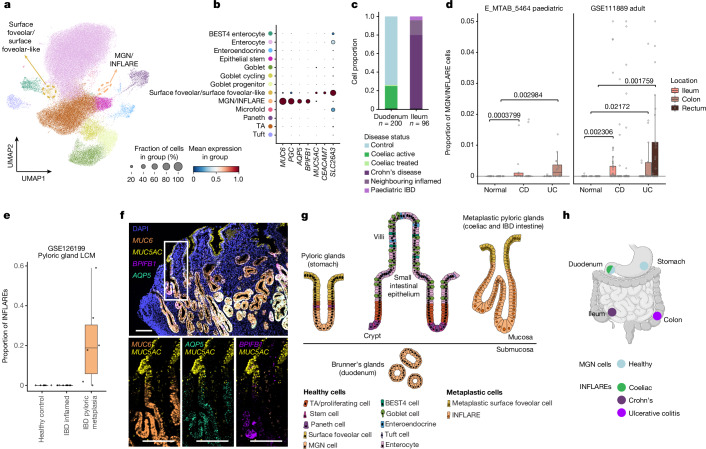
Identification of INFLAREs resembling pyloric or Brunner’s gland neck cells in health. **a**, UMAP showing cells from the small intestinal epithelium in the full atlas (healthy and diseased). MGN or INFLARE and surface foveolar cells, both involved in pyloric metaplasia, are highlighted with a dashed circle. **b**, Marker gene dotplot of pyloric gland cell markers (MGN and surface foveolar cells). The cell type legend is shared in **a** and **b**. **c**, Proportion of MGN or INFLAREs by disease category in the duodenum and ileum. **d**, Bulk deconvolution (BayesPrism) using disease intestinal epithelium as a reference in studies of Crohn’s disease (CD) and ulcerative colitis (UC). For E_MTAB_5464, *n* = 25 (CD), 27 (UC) and 27 (normal). For GSE111889, *n* = 122 (CD), 71 (UC) and 50 (normal). Numbers above brackets represent *P* values calculated by two-sided Wilcoxon rank-sum test. **e**, Bulk deconvolution as in **d** from the laser capture microdissection (LCM) epithelium from healthy crypts (*n* = 7), inflamed crypts from patients with IBD (*n* = 6) and metaplastic glands from patients with IBD (*n* = 6). For both **d** and **e**, the lower edge, upper edge and centre of the box represent the 25th (Q1) percentile, 75th (Q3) percentile and the median, respectively. The interquartile range (IQR) is Q3 − Q1. Outliers are values beyond the whiskers (upper, Q3 + 1.5 × IQR; lower, Q1 − 1.5 × IQR). **f**, smFISH staining of MGN and INFLARE cell marker genes (*MUC6*, *AQP5* and *BPIFB1*) and surface foveolar cell markers (*MUC5AC*) in a biopsy from the duodenum from a patient with Crohn’s disease and pyloric metaplasia. Representative images from *n* = 4. Scale bars, 100 µm. **g**, Organization of cells within the gastric glands in the stomach, small intestinal epithelium, Brunner’s glands and metaplastic pyloric glands. **h**, Schematic of MGN and INFLARE cell distribution across the stomach and intestines, defining MGN cells in the healthy stomach and duodenum and INFLAREs in the coeliac duodenum, Crohn’s disease ileum and ulcerative colitis colon. The schematic in panel **h** was created with BioRender (https://biorender.com).

**Extended Data Fig. 6:**
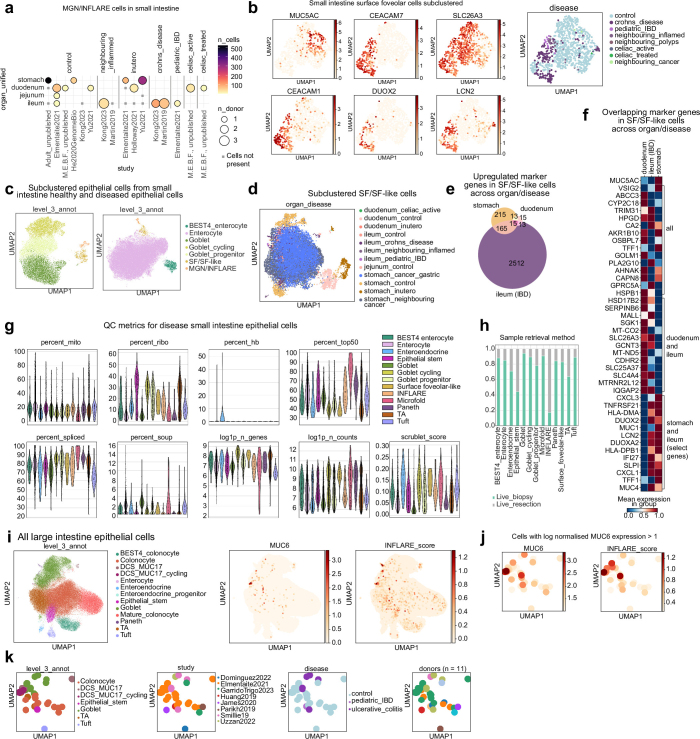
Identification of INFLAREs. a) Overview of the number of MGN (Mucous gland neck)/INFLAREs (Inflammatory Epithelial cells) and donors per study, broken down by age and region of the GI. Dot size indicates the number of donors, colour indicates the number of cells. b) UMAP of subclustered surface foveolar (SF) cells from small intestine, showing heterogeneity of marker genes and additional genes upregulated in disease cells annotated as SF cells (SF-like cells). c) UMAP of subclustered INFLAREs, SF/SF-like cells and either goblet or enterocyte populations, showing distinct separation of populations highlighting transcriptional differences. d) UMAP of subclustered SF and SF-like cells across the atlas, coloured by age, region and disease status. e) Overlap of SF/SF-like marker genes from different regions. Marker genes of SF/SF-like cells were calculated by differential gene expression (wilcoxon rank-sum test) of other stomach and small intestine epithelial cells separately for healthy adult stomach SF cells, healthy adult duodenum SF cells and ileum CD SF-like cells showing overlapping marker genes. f) Heatmap of overlapping marker genes calculated in (e) (with *MUC5AC* for reference) showing overlapping genes across all comparisons, healthy duodenum and CD ileum, and selected genes of the 165 overlapping in healthy stomach and CD ileum. g) Violin plot for QC metrics across epithelial cell subsets from diseased samples (mito = mitochondria, ribo = ribosomal, hb = haemoglobin). h) Stacked barplot for sample retrieval method for cells in disease small intestinal samples, highlighting that the majority of INFLAREs come from resections rather than biopsies. i) UMAP of epithelial cells from large intestine, with added data from studies^8,100^ (totalling an additional 209,347 cells from 23 control, 24 CD and 23 UC patients) coloured by cell type, *MUC6* gene expression and gene score for INFLARE markers (*MUC6, BPIFB1, AQP5, PGC*). j) Cells from (i) filtered by log-normalised *MUC6* expression greater than 1, coloured by *MUC6* gene expression and INFLARE marker score. k) Cells from (j) coloured by cell type, study, disease and donor.

In the small intestine, we observed two distinct epithelial populations with unique signatures across healthy and diseased samples. In the healthy duodenum, we observed *MUC6*^+^ mucous gland neck (MGN) cells and *MUC5AC*^+^ surface foveolar cells phenotypically resembling cells of the Brunner’s glands^25,26^ (Fig. 3a,b, Extended Data Fig. 6a, Supplementary Fig. 2e and Supplementary Notes 4 and 5). As expected, these cells were abundant in stomach samples, representing cells of the pyloric glands (Extended Data Fig. 6a and Supplementary Fig. 2d). Disease cells annotated as MGN or surface foveolar populations were enriched in the ileum of patients with IBD (Fig. 3c and Extended Data Figs. 4a and 6b,c). In the duodenum of patients with untreated coeliac disease, we observed more *MUC6*^+^ cells than in matched controls (Extended Data Fig. 6a). Marker genes of the MGN-like population included *MUC6*, *PGC*, *AQP5* and *BPIFB1* (Fig. 3b). Within the surface foveolar-like population in disease, we observed enhanced and heterogeneous expression of *CEACAM7*, *CEACAM1*, *DUOX2* and *LCN2* (Extended Data Fig. 6b). Owing to the low *MUC5AC* expression in scRNA-seq (Extended Data Fig. 6c–f and Supplementary Note 5), we refer to this distinct population in disease as ‘surface foveolar-like’.

In the coeliac duodenum and IBD ileum, we hypothesized that *MUC6*^+^ cells represent epithelial cells in pyloric metaplasia^13^ and provide additional supporting evidence in Supplementary Note 4 (Extended Data Fig. 6g–k). In previous studies of the diseased small intestine, *MUC6*^+^ cells were either annotated as a mixture of cell types (including microfold cells, *OLFM4*^+^ stem cells and goblet cells) or excluded entirely (Supplementary Fig. 10). By contrast, here we identified *MUC6*^+^ cells in the coeliac duodenum and IBD ileum as epithelial cells in pyloric metaplasia. This discovery reflects the power of data integration to classify rare cell types (for supporting evidence, see Supplementary Note 4). We henceforth refer to *MUC6*^+^ cells in disease as INFLAREs to distinguish them from healthy MGN cells. We next investigated the molecular and cellular roles of this metaplastic lineage in disease.

**Extended Data Fig. 7:**
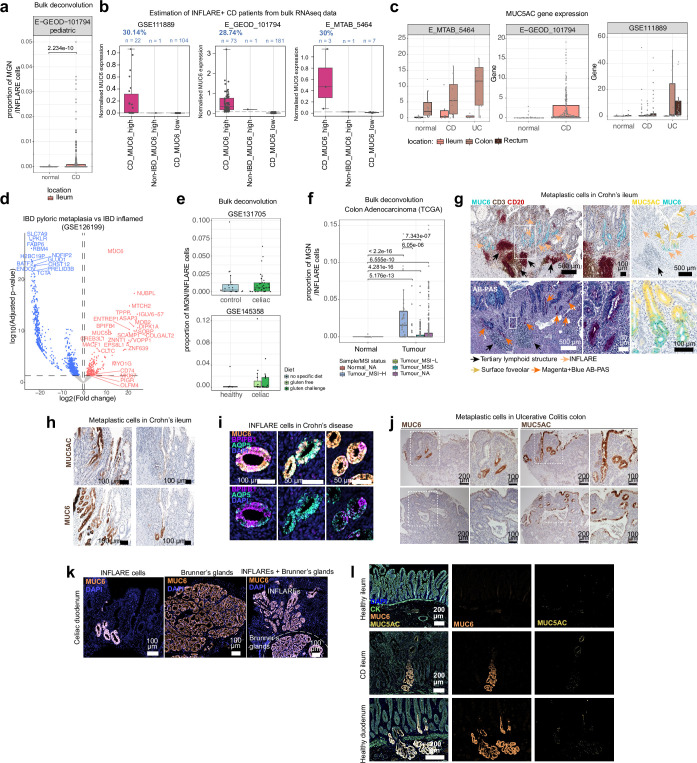
Validation of INFLAREs. a) Deconvolution (BayesPrism) of bulk RNAseq dataset comparing MGN and INFLAREs in healthy (normal, n = 50) and CD (n = 254). Statistical analysis was performed using two-sided Wilcoxon rank-sum test. b) Estimation of CD patients with INFLAREs based on stratification of high and low MUC6 expressing samples from the bulk datasets indicated, showing ~29% of patients have high MUC6 expression. c) Expression of *MUC5AC* from bulk datasets indicated comparing expression in controls, CD and UC patients. d) Differential gene expression analysis (DESeq2) from laser capture microdissected epithelium from healthy crypts (n = 7), inflamed crypts from IBD patients (n = 6) and metaplastic glands from IBD patients (n = 6) from published data (GSE126199). Genes with a log2FC greater than 0 are upregulated in metaplastic glands compared to inflamed IBD epithelium, based on two-sided Wald test with Benjamini and Hochberg correction. e) Deconvolution (BayesPrism) of bulk RNAseq from celiac disease comparing MGN and INFLARE proportions in healthy and celiac disease tissue. For GSE131705, n = 21 (healthy) and n = 33 (celiac). For GSE145358, n = 6 (healthy), n = 15 (celiac gluten free) and n = 15 (celiac gluten challenge). f) Deconvolution (BayesPrism) of TCGA bulk RNAseq data of MGN and INFLAREs in healthy tissue (normal, n = 41) and tumour tissue stratified by microinstability status, n = 40 (Tumour_MSI-H), n = 42 (Tumour_MSI-L), n = 126 (Tumour_MSS) and n = 272 (Tumour_NA). MSI-high tumours are predicted to have higher levels of INFLAREs. Statistical analysis was performed using two-sided Wilcoxon rank-sum test. For all box and whisker plots the lower edge, upper edge and centre of the box represent the 25th (Q1) percentile, 75th (Q3) percentile and the median, respectively. The interquartile range (IQR) is Q3 - Q1. Outliers are values beyond the whiskers (upper, Q3 + 1.5 x IQR; lower, Q1 − 1.5 x IQR). g) Protein and ABPAS (Alcian Blue Periodic acid-Schiff) staining of INFLAREs (MUC6, Magenta+Blue+ ABPAS staining) and metaplastic surface foveolar cells (MUC5AC) in CD ileum showing association with tertiary lymphoid structures (dense nuclei and CD3/CD20+ regions). Selected regions adjacent to lymphoid structures from n = 2 (CD3, CD20, MUC6 staining), n = 2 (AB-PAS staining) and n = 2 (MUC5AC, MUC6 staining). h) Protein staining of INFLAREs (MUC6) and metaplastic surface foveolar cells (MUC5AC) from CD ileum tissue from additional donors (n = 3). i) smFISH staining of INFLARE (Inflammatory Epithelial cell) markers (*MUC6, AQP5* and *BPIFB1*) in pyloric metaplasia of CD duodenum showing heterogeneity in *AQP5* and *BPIFB1* expression (n = 4). j) Protein staining of INFLAREs (MUC6) and metaplastic surface foveolar cells (MUC5AC) in colon resection tissue from UC patients (n = 3). Upper and lower panels are images from two different patients. k) Protein staining of MGN and INFLAREs (MUC6) in celiac disease duodenum showing INFLAREs and healthy MGN cells in Brunner’s gland in the submucosa (n = 2). l) Protein staining of MUC6, MUC5AC and cytokeratin (CK) in healthy ileum (n = 4), CD ileum (n = 4) and healthy duodenum (n = 2). All images show representative staining from the replicates indicated.

Pyloric metaplasia has been reported in approximately 28% of patients with IBD via histology^13,27,28^ (Supplementary Table 3). In our atlas, we found INFLAREs in only a small number of patients, potentially due to sampling biases (Extended Data Fig. 6h). To generalize our findings, we investigated bulk RNA-seq datasets of mucosal biopsies from paediatric and adult patients with IBD. Using bulk deconvolution with our single-cell data as a reference (Methods), we found significantly higher proportions of INFLAREs in Crohn’s disease and ulcerative colitis samples and microdissected metaplastic tissue than in healthy tissue, which agreed with previously reported prevalence and validated INFLARE marker genes (Fig. 3d,e, Extended Data Fig. 7a–d and Supplementary Note 6). INFLAREs were present across the intestines in Crohn’s disease but only in the large intestines of patients with ulcerative colitis, consistent with the aetiology and site of inflammation (Fig. 3d), and also detected in patients with coeliac disease and in patients with CRC with microsatellite instability (Extended Data Fig. 7e,f and Supplementary Note 6). MUC6 expression is associated with colonic neoplasms in ulcerative colitis, suggesting that INFLAREs may have a direct role in colitis-associated CRC^29,30^.

To validate the presence of INFLAREs in patients with IBD and coeliac disease, we performed immunohistochemistry and multiplexed single-molecule fluorescence in situ hybridization (smFISH) in patient samples (Supplementary Table 4). We located INFLAREs (*MUC6*^+^*AQP5*^+^*BPIFB1*^+^) at the crypt base and surface foveolar cells (*MUC5AC*^+^) at the crypt top of metaplastic glands in Crohn’s disease mucosa (Fig. 3f–h and Extended Data Fig. 7g,h). We noted heterogeneity in INFLAREs based on co-expression of *AQP5* and *BPIFB1* (Extended Data Fig. 7i) and observed their close association with ulcerated regions and tertiary lymphoid structures (Extended Data Fig. 7g). We also validated INFLAREs in disease tissue from untreated patients with coeliac and ulcerative colitis (Extended Data Fig. 7j,k). In untreated patients with coeliac disease, MUC6^+^ INFLARE metaplastic glands were distinguished from healthy MUC6^+^ Brunner’s gland cells by their mucosal localization (Extended Data Fig. 7k, left panel). MUC6^+^ or MUC5AC^+^ cells were not found in the healthy ileum (Extended Data Fig. 7l). Thus, INFLAREs are found across the intestines during chronic inflammation and share transcriptional similarities to healthy MGN cells, which are restricted to the stomach and duodenum (with important differences discussed below) (Fig. 3g). We describe INFLAREs, *MUC6*^+^ cells of pyloric metaplasia, at single-cell resolution for the first time, to our knowledge.

## Origin of INFLAREs

**Fig. 4:**
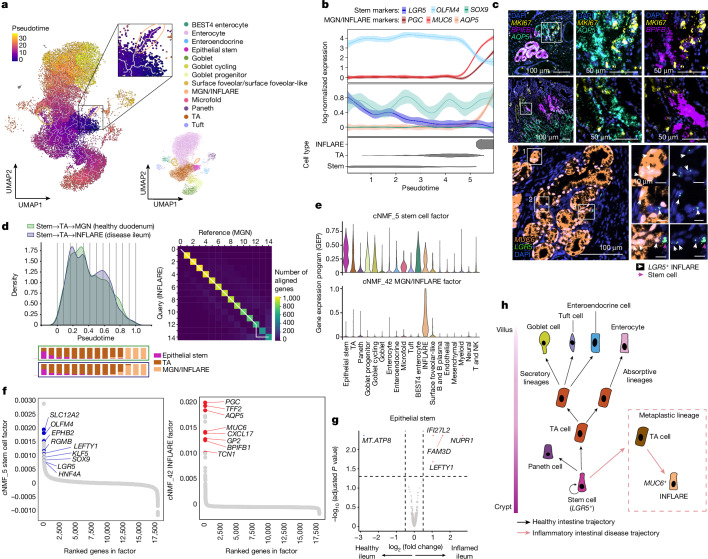
INFLAREs originate from stem cells and retain stem-like properties. **a**, UMAP of small intestinal epithelial cells coloured by pseudotime trajectory (Monocle3). Cells are from the ileum of inflamed IBD samples from studies^5,6,22^. INFLAREs are highlighted using the inset, and the UMAP plot on the right indicates cell types. **b**, Expression of key genes along the stem → TA → INFLARE trajectory. The error bands correspond to the mean ± 95% CI of log-normalized gene expression. **c**, Proliferation (*MKI67*) and stemness (*LGR5*) gene expression by smFISH in INFLAREs (*MUC6*^+^) from the Crohn’s disease ileum and duodenum. Representative image from *n* = 4. **d**, Alignment of Palantir pseudotime trajectories (Extended Data Fig. 8b) for stem → TA → INFLARE (disease ileum) and stem → TA → MGN (healthy duodenum) using Genes2Genes^68^. The cell density of the aligned trajectories, marked with 14 interpolation time bins, and the corresponding cell-type proportions of those bins as stacked barplots (left). The average alignment path (white line) of 1,171 transcription factors along the trajectories (right) is also shown. Each matrix cell of the heatmap gives the number of transcription factors with matched pseudotime points. **e**, Violin plots showing the expression of genes in factors from cNMF analysis related to MGN or INFLAREs and stem cells (*LGR5*^+^), across all small intestinal cells. **f**, Rankings of genes in factors 5 (stem cell factor) and 42 (MGN and INFLARE factor). The genes involved in stem cell function (blue) and MGN and INFLARE markers (red) are shown. **g**, Differential gene expression analysis comparing stem cells from control (*n* = 8) and IBD (*n* = 18) ileal pseudobulk samples. The genes with positive log_2_ fold change are upregulated in IBD compared with healthy samples, based on two-sided Wald test with Benjamini–Hochberg correction. **h**, Schematic of epithelial cell trajectories along the crypt–villus axis in the healthy small intestine (black arrows) and in inflammatory diseases (red arrows and dashed box), as hypothesized in our study.

**Extended Data Fig. 8:**
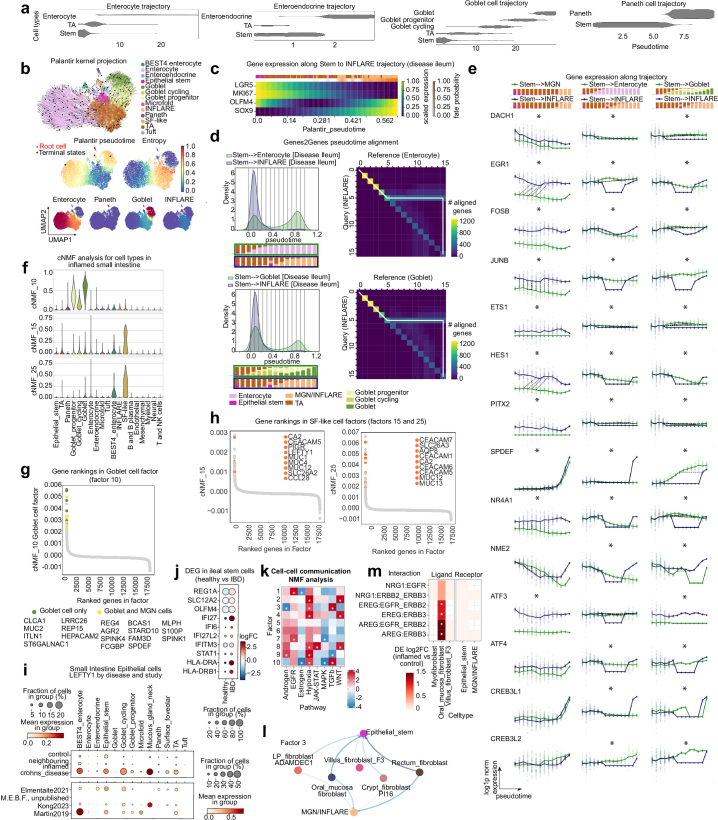
Origins and stem-like features of INFLAREs. a) Relative cell proportions along healthy trajectories as calculated by Monocle3, to give confidence in the reconstruction of known trajectories. b) Palantir trajectory analysis from remapped studies, showing CellRank kernel projection and pseudotime of 4 terminal cell states in inflamed ileum. c) Scaled expression of stem markers as in Fig. 4b in the Palantir pseudotime trajectory for INFLAREs. d) Genes2Genes alignment of Palantir pseudotime trajectories for stem → INFLARE compared with stem → enterocyte and stem → goblet in inflamed ileum. Left: Cell density plots of the aligned trajectories along pseudotime, marked with 15 interpolation time points (bins) used for each alignment, and the corresponding cell-type proportions of those bins as stacked bar plots for each comparison. Right: Overall average alignment paths (highlighted in white) of the 1262 transcription factors between the interpolation pseudotime points along the trajectories for both comparisons. Each matrix cell of the pairwise heatmap gives the number of TFs where the corresponding pseudotime points have been matched. e) Mismatched genes (alignment similarity ≤ 50% and optimal alignment cost ≥ 30 nits) in INFLARE compared to control trajectories as indicated, showing their pseudotime alignments in (d) and Fig. 4d using Genes2Genes. Bold lines represent mean expression trends and faded data points are 50 random samples from the estimated expression distribution at each time point. The black dashed lines visualise matches between time points. Asterix indicates significant mismatch in gene alignment (as outlined above) for the specific gene/trajectory comparison. f) cNMF analysis (Methods) of cell types from IBD small intestine in the atlas. Violin plots showing expression of ranked genes in factors related to SF-like cells and goblet cells. g) Gene rankings of genes in factor 10 (goblet cell factor) with goblet cell specific genes highlighted in green and those also expressed in Mucous gland cells (MGN and INFLARE and SF-like cells) highlighted in yellow. h) Gene rankings of genes in factors 15 and 25 (SF-like cell factors) with select genes highlighted. i) Dotplot of *LEFTY1* expression in small intestine epithelial cells across cell types and conditions (upper) and across cell types and study (lower). j) Dot plot of selected differential expressed genes (wilcoxon rank-sum test) in epithelial stem cells (*LGR5*+) from the ileum of patients with IBD compared with healthy controls. k) NMF factors from cell-cell communication analysis using ligand/receptor mean expression and cell type pairs to determine factors. Heatplot shows the expression of ligand/receptor pairs categorised into pathways for each factor. l) Connectivity of high ranking cell types in factor 3, showing interactions between fibroblasts (sources) and epithelial stem cells or INFLAREs (targets). Line thickness indicates a higher number of ligand/receptor pairs per cell type pairing. m) Expression (log2FC from DESeq2) comparing ligand and receptor expression in healthy controls vs IBD samples in relevant cell types from (l) for ligands and receptors within the NRG1/AREG/EREG pathway. Positive log2FC indicates upregulation of ligand/receptor expression in IBD compared to healthy controls.

To interrogate the origin of INFLAREs, we performed trajectory analysis (Methods) on small intestinal epithelial cells (Fig. 4a and Extended Data Fig. 8a,b). INFLAREs branched from *LGR5*^+^ stem cells (Fig. 4a) and retained expression of stemness genes along the trajectory (Fig. 4b and Extended Data Fig. 8c). Using smFISH, we found *LGR5* and *MKI67* expression in INFLAREs in tissue from the ileum of individuals with Crohn’s disease (Fig. 4c), validating a stemness and proliferative phenotype.

To identify drivers of the INFLARE trajectory, we performed gene-level pseudotime trajectory alignment of stem cells to either MGNs or INFLAREs (Fig. 4d) or to other inflamed lineages (enterocytes and goblet cells) from the duodenum (Methods; Extended Data Fig. 8b–d). We focused our analysis on transcription factors, due to their importance in determining cell fates, and found 19 mismatched transcription factors (potentially involved in determining INFLARE cell fate) (Extended Data Fig. 8e and Supplementary Note 7). These transcription factors have been implicated in the regulation of stem cells, intestinal development and secretory programs, the epithelial injury response and metaplasia (Supplementary Note 5). In addition, we found mismatches across two of three comparisons in *NME2*, which is implicated in maintaining gastric cancer stemness^31^, and *ATF3*, *ATF4*, *CREB3L1* and* CREB3L2*, which encode cAMP response element-binding proteins implicated in injury responses and metaplasia in the stomach and pancreas^32,33^. These mismatched transcription factors highlight potentially conserved molecular mechanisms (inflammatory stress responses and tissue regeneration programs) for mucous cell metaplasia across tissues.

Applying cNMF analysis to diseased cells in the small intestine, we identified transcriptional programs shared between epithelial populations and INFLAREs. A stem cell gene program (Fig. 4e, factor 5) with high-ranking genes including *SLC12A2*, *RGMB* and *LGR5* (Fig. 4f) was highly expressed in INFLAREs. Other factors distinguished MGN and INFLAREs from other mucous-secreting cells, such as the INFLARE signature itself (factor 42), surface foveolar-like (factors 15 and 25) and goblet signatures (factor 10; Fig. 4e,f and Extended Data Fig. 8f), with the latter two including expected cell-type-specific genes (Extended Data Fig. 8f–h). INFLAREs are thus a distinct cell type with unique transcriptional signatures and expression of stemness genes.

Comparing stem cell gene expression, *LEFTY1*, a marker of intestinal metaplasia progenitors in the stomach and oesophagus, was enriched in inflamed versus healthy ileum (Fig. 4g, Extended Data Fig. 8i and Supplementary Note 8). *REG1A*, *OLFM4* and *SLC12A2* were also enriched in IBD (Extended Data Fig. 8j), suggesting that inflamed stem cells differ from those in healthy tissue, which may explain their potential to give rise to metaplastic cells. Cell–cell communication analysis highlighted differentially regulated stem cell factors that may contribute to a metaplastic niche. In particular, we identified the ligands *NGR1*, *AREG* and *EREG*, which were upregulated in oral mucosa/inflammatory fibroblasts and signalled to stem cells and INFLAREs via *EGFR*, *ERBB2* and *ERBB3* (Extended Data Fig. 8k–m and Supplementary Note 9).

Together, our data suggest that metaplasia can arise from inflammation-induced changes within crypt-based stem cells giving rise to INFLAREs, the major lineage of pyloric metaplasia (Fig. 4h). Moreover, INFLAREs retain stem-like properties in intestinal disease, representing a plastic population.

## Dual role of INFLAREs in disease

**Extended Data Fig. 9:**
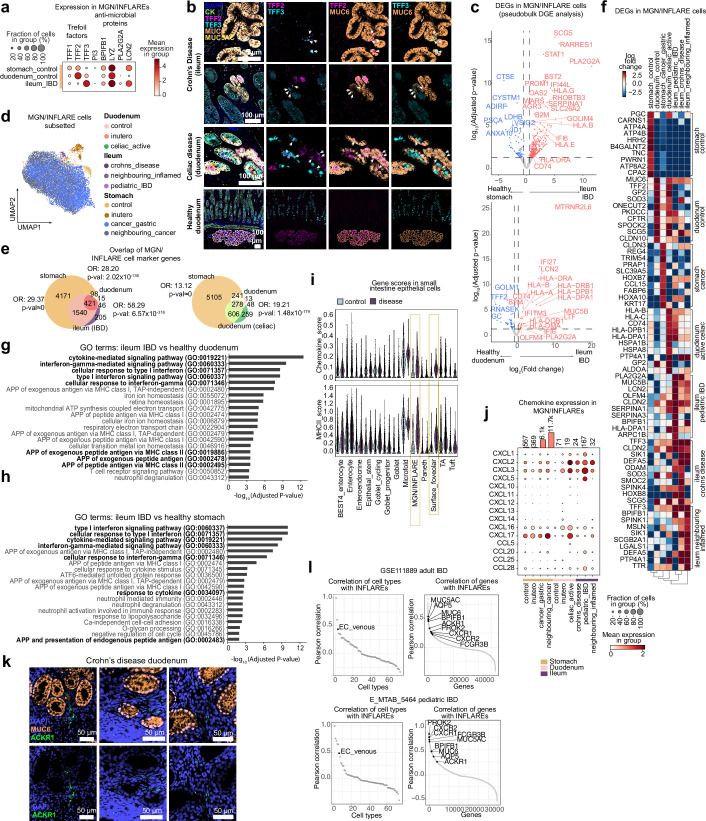
Dual role of pyloric metaplasia in mucosal healing and inflammation. a) Expression of genes related to mucosal barrier function in MGN (Mucous gland neck)/INFLAREs (Inflammatory Epithelial cells) in healthy stomach, healthy duodenum and IBD ileum. b) Protein staining of TFF2, TFF3, MUC6 (MGN and INFLARE), MUC5AC (surface foveolar) and cytokeratin (CK) across from CD ileum (n = 4), celiac duodenum (n = 2) and healthy proximal duodenum (n = 2). White arrows indicate MUC6 + TFF3+ cells. c) Pseudobulk (decoupler) and differential gene expression analysis (DESeq2) comparing INFLAREs from IBD ileum (n = 4 pseudobulk samples) with MGN from healthy stomach (n = 35) or healthy duodenum (n = 5) with INFLAREs from IBD ileum. Genes with positive log2FC are upregulated in INFLAREs compared with healthy cells, based on two-sided Wald test with Benjamini and Hochberg correction. d) Subclustered MGN and INFLAREs from across the atlas (locations, ages and diseases). MGN and INFLAREs from different regions and/or developmental stages (ie. *in utero*) occupy separate coordinates in the UMAP. e) Overlap of MGN and INFLARE marker genes from different regions. Marker genes of MGN and INFLAREs were calculated by differential gene expression (wilcoxon rank-sum test) of other stomach and small intestine epithelial cells separately for healthy adult stomach MGN, healthy adult duodenum MGN, ileum CD INFLARE and duodenum celiac disease INFLARE. Overlapping marker genes show greater similarity of INFLAREs to healthy adult stomach MGN cells, than to healthy adult duodenum MGN cells. f) Heatmap of differentially expressed genes (wilcoxon rank-sum test) in MGN and INFLAREs across healthy and diseased adult conditions. Stomach control is combined control and neighbouring cancer stomach MGN cells. g) GO terms from upregulated genes (wilcoxon rank-sum test) in IBD INFLAREs (CD and pediatric IBD) compared with healthy control duodenum. Highlighted pathways are inflammatory, MHC-II mediated antigen presentation and exogenous peptide antigen presentation related pathways. h) Analysis as in (g) comparing IBD INFLAREs to healthy control stomach. i) Chemokine and MHC-II gene scores (see Supplementary Table 5 for gene list) comparing small intestine epithelial cells in the atlas in healthy control and disease (IBD and celiac) samples showing specificity of upregulated chemokine and MHC-II related gene expression in particularly in INFLAREs vs MGN cells. j) Expression of chemokines in MGN and INFLAREs, across healthy and diseased tissues. k) Additional smFISH staining (as in Fig. 5c, representative from n = 3) of INFLAREs (*MUC6*) association with ACKR1+ vessel in CD duodenum. l) Correlation between INFLARE cell proportions and cell types/genes from deconvolution (BayesPrism) of bulk RNAseq adult and pediatric IBD datasets using the atlas as a reference. Analysis indicates consistent correlation of EC_venous cells (*ACKR1*+ endothelial population) with INFLAREs, and metaplastic surface foveolar and neutrophil marker genes with INFLAREs.

Previous studies have suggested that metaplasia is an adaptation in mucosal tissues in response to injury and healing^4,34^. Supporting this hypothesis, INFLAREs expressed *TFF3*, a trefoil factor normally expressed by goblet cells, which has a key role in mucosal healing^35^ and causes mucinous metaplasia and neutrophil infiltration in fundic glands when overexpressed in mice^36^. By contrast, healthy MGN cells in the stomach and duodenum expressed mostly *TFF2* (Extended Data Fig. 9a,b). INFLAREs had significantly decreased *TFF2* expression and also increased expression of *PLA2G2A*, which encodes an antibacterial protein important for the stem cell niche^37,38^ (Extended Data Fig. 9c).

However, INFLAREs also expressed programs that may contribute to chronic intestinal inflammation. We compared MGN and INFLAREs across different tissues, life stages and diseases in our atlas, identifying distinct features depending on the context (Extended Data Fig. 9d). We found greater similarity between diseased INFLAREs and healthy MGN cells in the stomach than in the healthy duodenum (Extended Data Fig. 9e,f). Compared with MGN cells in the healthy duodenum and stomach, INFLAREs upregulated cytokine-induced inflammatory programs and IFNγ-mediated pathway genes, similar to ileal stem cells from patients with Crohn’s disease (Extended Data Fig. 9c,g,h).

**Fig. 5:**
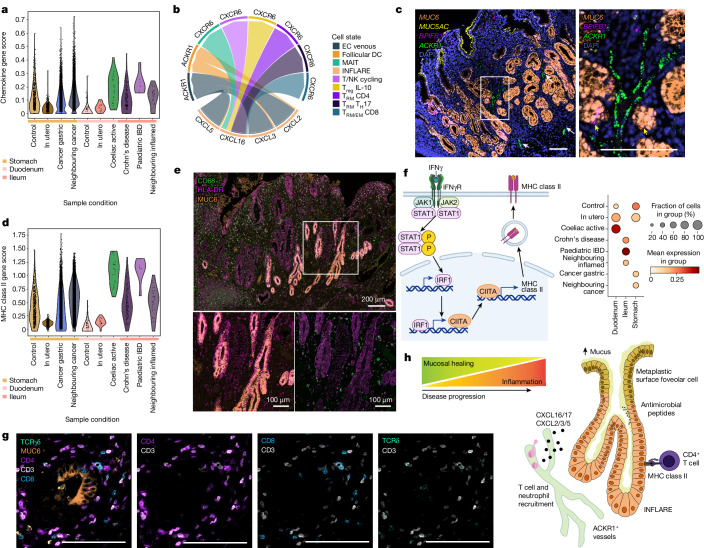
INFLAREs recruit and interact with immune cells in IBD. **a**, Gene score of chemokines across MGN and INFLAREs from the stomach, duodenum and ileum across different conditions. **b**, Cell–cell interactions mediated by CXCL chemokines expressed by INFLAREs and various immune cells or venous endothelial cells (ECs). MAIT, mucosal-associated invariant T cell; T_EM_, effector memory T cell; T_H_17, T helper 17 cell; T_reg_, regulatory T cell; TRM, tissue resident memory T cell. **c**, smFISH staining of INFLARE (*MUC6* and *BPIFB1*), surface foveolar (*MUC5AC*) and activated endothelial (*ACKR1*) cells showing the proximity of vessels to metaplastic glands in Crohn’s disease duodenum. Representative image from *n* = 3. Scale bars, 100 µm. White arrows highlight *ACKR1*^+^ vessels, yellow arrows indicate *BPIFB1*^+^*MUC6*^+^ cells. For both images, the scale bar represents 100 μm. **d**, Gene score of MHC class II genes and peptide processing genes across MGN and INFLAREs from the stomach, duodenum and ileum across different conditions. **e**, Protein staining of INFLAREs (MUC6), macrophages (CD68) and MHC class II (HLA-DR) in the ileum from a Crohn’s disease resection showing high MHC class II expression in INFLAREs. Representative image from *n* = 2. **f**, Schematic of the signalling pathway from IFNγR to MHC class II (left), with a dotplot of gene scores from this pathway in MGN and INFLAREs from the stomach, duodenum and ileum across different conditions (right). Schematics in panel **f** were created with BioRender (https://biorender.com). **g**, Protein staining of INFLAREs (MUC6), CD4 T cells (CD3^+^CD4^+^), CD8 T cells (CD8^+^CD3^+^) and γδ T cells (TCRγδ^+^CD3^+^) in Crohn’s disease ileum, showing interaction between CD4 T cells and INFLAREs. Representative image from *n* = 4. Scale bars, 100 µm. **h**, Schematic of the potential role of pyloric metaplasia in inflammatory intestinal diseases. INFLAREs arise in response to local inflammation to promote mucosal healing via mucous and antimicrobial peptide secretion. As disease progresses, INFLAREs contribute to ongoing inflammation through association with activated vessels, the recruitment of various immune cells and direct interactions with CD4^+^ T cells via MHC class II.

To interrogate inflammatory signalling from INFLAREs in disease, we performed cell–cell interaction analysis (Methods). INFLAREs overexpressed the chemokines *CXCL16* (T cell recruiting), *CXCL2*, *CXCL3* and *CXCL5* (neutrophil recruiting) and *CXCL17* (myeloid-recruiting angiogenic factor^39^) compared with healthy MGN cells (Fig. 5a,b and Extended Data Fig. 9i). Healthy stomach MGN cells more closely resembled INFLAREs, with upregulated chemokine expression compared with healthy duodenum MGN cells (Fig. 5a and Extended Data Fig. 9i,j). *CXCL2*, *CXCL3* and *CXCL5* on INFLAREs were predicted to interact with *ACKR1*, which encodes an atypical receptor that can transport chemokines into the vessel lumen^40^, on venous endothelial cells (Fig. 5b). *ACKR1* expression in the endothelium is associated with resistance to anti-TNF and anti-integrin α4β7 therapy in IBD^6^ and can be upregulated through neutrophil interactions^40^. Using smFISH, we found a close association of *ACKR1*^+^ vessels with INFLAREs in Crohn’s disease tissue (Fig. 5c and Extended Data Fig. 9k). In agreement, venous endothelial cells correlated with INFLAREs in deconvoluted bulk RNA-seq data from Crohn’s disease tissue (Extended Data Fig. 9l). Neutrophil marker genes (*CXCR1*, *CXCR2*, *FCGR3B* and *PROK2*) also correlated with INFLAREs in bulk RNA-seq data (Extended Data Fig. 9l). Together, INFLAREs express immune-recruiting chemokines, which could potentiate inflammation in intestinal diseases.

**Extended Data Fig. 10:**
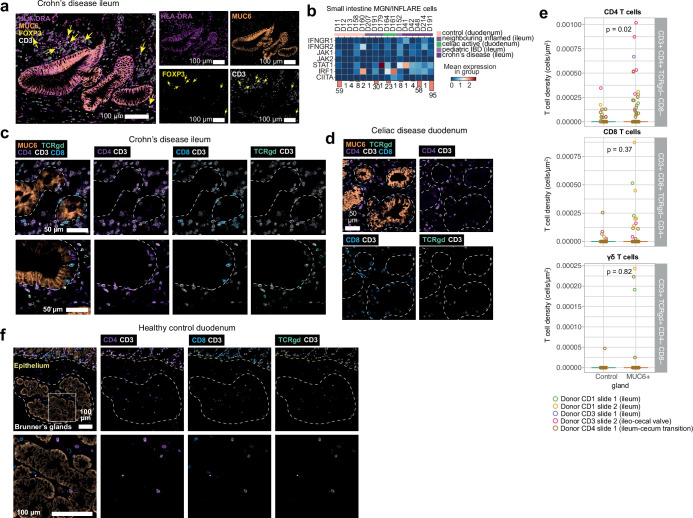
INFLARE:T cell interactions. a) Protein expression in CD ileum (representative of n = 2) of HLA-DR (MHC-II) in INFLAREs (MUC6) along with localisation of CD3+ T cells and regulatory T cells (FoxP3+CD3+). b) Expression per donor of genes involved in IFNGR to MHC-II signalling pathway in INFLAREs and MGN cells in small intestine, as summarised in Fig. 5f. c) Additional protein staining for INFLAREs (MUC6) in CD disease ileum (as in Fig. 5g, n = 4) with various T cell subsets (CD4+CD3+, CD8+CD3+, TCRγδ+CD3+T cells). d) Protein staining as in (c) in Celiac disease duodenum tissue (n = 2). e) Quantitation of T cell densities for the T cell subsets indicated in MUC6+ glands and adjacent control epithelium across 5 sections from 3 donors as represented in (c). P-values calculated based on ROIs as replicates (n = 126 MUC6+ ROIs and 59 adjacent control ROIs) using negative binomial linear regression, adjusting for log area, two-sided Wald test. f) Protein staining as in (c) and (d) in healthy proximal duodenum (n = 2) showing abundance and localisation of T cell subsets in Brunner’s glands.

In addition to inflammatory cytokines, INFLAREs have elevated MHC class II-related gene expression compared with healthy MGN cells, particularly those in the duodenum (Fig. 5d, Extended Data Fig. 9c,g–i and Supplementary Note 8). We confirmed this at the protein level in ileum sections from patients with Crohn’s disease, showing that INFLAREs had much higher HLA-DR expression than surrounding MUC6^−^ glands and surface epithelium (Fig. 5e and Extended Data Fig. 10a). Elevated levels of MHC was seen in other epithelial cells from inflamed tissue, including surface foveolar-like and *LGR5*^+^ stem cells; however, this increase was most prominent in INFLAREs compared with healthy MGN cells (Extended Data Fig. 9i). We observed increased IFNγ response signatures in INFLAREs from inflamed versus healthy tissue, consistent with the abundance of IFNγ in the inflamed intestine and its role in MHC class II regulation^41^ (Fig. 5f and Extended Data Figs. 9g,i and 10b). In addition, we observed CD8^+^, CD4^+^ and γδ T cells surrounding INFLAREs in Crohn’s disease and coeliac disease tissue, in contrast to low numbers of T cells surrounding healthy Brunner’s glands (Fig. 5g and Extended Data Fig. 10c–f). INFLAREs had higher densities of CD4 T cells (significant using regions of interest as replicates) than in neighbouring MUC6^−^ glands (Extended Data Fig. 10e). Consistent with elevated MHC class II, close interaction between CD4^+^ T cells and INFLAREs in the Crohn’s disease ileum suggests that INFLAREs may act as non-conventional professional antigen-presenting cells in chronic inflammation. Overall, in addition to the mucosal healing hypothesis for pyloric metaplasia, INFLAREs can exacerbate chronic inflammation through interactions with immune cells with known roles in IBD and coeliac pathogenesis (Fig. 5h).

## Discussion

Here we present an integrated single-cell atlas covering the whole human gastrointestinal tract and a workflow including bioinformatic tools (scAutoQC) that can aid the assembly of other large-scale atlases. Systematic regional comparisons between health and disease revealed metaplastic lineages with cellular identities of other gastrointestinal regions in chronic disease, including Paneth cells, oral mucosa/inflammatory fibroblasts and INFLAREs.

MGN cells, the healthy counterpart of INFLAREs, are best described in the healthy stomach and healthy duodenal Brunner’s glands^26^. A scRNA-seq study of paediatric treatment-naive patients with Crohn’s disease identified *MUC6*^+^*TFF2*^+^ and *BPIFB1*^+^*AQP5*^+^ populations, albeit annotated as goblet cells^42^. Similarly, another study of Crohn’s disease and ulcerative colitis identified INFLAREs as *MUC6*^+^*PGC*^+^*DUOX2*^+^ enterocytes, enriched in the inflamed Crohn’s disease ileum^43^. Pyloric metaplasia in patients with Crohn’s disease has been reported extensively from histology^13^ and we now annotate and interrogate pyloric metaplasia at the single-cell level, with full transcriptional resolution for the first time. We highlight distinguishing features of INFLAREs from their healthy counterparts and define changes both in stem cells and in mature, differentiated cells across intestinal inflammatory diseases.

Our observations support the view that metaplasia arises due to alterations in stem cell identity and differentiation. Recent studies in the oesophagus^12^ and stomach^44^ have proposed that metaplastic lineages emerge from altered undifferentiated stem cells. In the ileum of patients with IBD, we propose a similar change, in which intestinal injury promotes stem cell differentiation to INFLAREs. We provide multiple lines of evidence for stem-like features in INFLAREs. The mechanisms of pyloric metaplasia may partly mirror the mechanisms of intestinal metaplasia of the oesophagus and stomach^45^. We found that INFLAREs express genes and pathways implicated in intestinal metaplasia, for instance, *LEFTY1* and *NRG1*–*ERBB3*. Although the precise mechanisms of stem cell transition to INFLAREs will be the focus of future research, we highlight potential mechanisms, including inflammatory signalling pathways, stem and tissue regeneration factors and cell–cell communication pathways.

Pyloric metaplasia may arise to repair the mucosal barrier after injury^4^. Our results build on these observations, proposing that INFLAREs also recruit and interact with immune cells. Increased MHC class II expression on intestinal epithelial cells in patients with IBD has been described, along with functional interactions between epithelial cells and CD4^+^ T cells via MHC class II^46,47^. We propose that INFLAREs similarly interact directly with CD4^+^ T cells under inflammatory conditions. In addition, INFLAREs can recruit neutrophils, similar to inflammatory fibroblasts^48^, using a cellular circuit probably aided by the close association with *ACKR1*^+^ vessels. In support of a disease-promoting role, many genes expressed by INFLAREs have been implicated in genome-wide association studies of IBD, including chemokines *CXCL1*, *CXCL2*, *CXCL3* and *CXCL5* and IFNγ signalling genes^49^.

In conclusion, we present an integrated single-cell atlas along the gastrointestinal tract as a resource to study gastrointestinal cell populations in health, development and disease. Using our atlas, we identify and interrogate pyloric metaplasia, informing the origin and role of metaplastic cells in intestinal inflammation and potential progression to neoplasia.

## Methods

### Patient samples and tissue processing

#### Healthy tissue from adults

Healthy adult gastrointestinal tissue was obtained by the Cambridge Biorepository of Translational Medicine (CBTM) from deceased transplant organ donors (*n* = 2) after ethical approval (REC 15/EE/0152, East of England–Cambridge South Research Ethics Committee) and informed consent from the donor families. Details of the gastrointestinal regions processed and donor information are compiled in Supplementary Table 5. Donors were perfused with cold University of Wisconsin (UW) solution, fresh tissue was collected from the distal stomach (antrum/pylorus), duodenum and terminal ileum within 1 h of circulatory arrest, and tissue was stored in HypoThermosol FRS preservation solution (H4416, Sigma) at 4 °C until processing. Intestinal tissue was open longitudinally and rinsed with D-PBS and then processed to single-cell suspensions following standard protocols^5,58^. For tissues from donor A68/759B (D105), epithelium and lamina propria were separated into different fractions by dissection. Epithelial cells were removed by washing the intestinal mucosa twice in Hank’s balanced salt solution (HBSS) medium (Sigma-Aldrich) containing 5 mM EDTA (15575020, Thermo Fisher), 10 mM HEPES (42401042, Gibco), 2% (v/v) FCS supplemented with 10 mM ROCK inhibitor (Y-27632 (Y0503, Merck)) while shaking at 4 °C for 20 min. Epithelial wash-offs were centrifuged at 300*g* for 7 min at 4 °C and incubated at 37 °C with TrypLE (Thermo Fisher) supplemented with 0.1 mg ml^−1^ DNase I (11284932001, Sigma) for 5 min. Cells were pelleted and filtered through a 40-μm cell strainer and resuspended in Advanced DMEM F12 (12634028, Thermo Fisher) with 10% (v/v) FCS. The remaining epithelium-depleted tissue was minced and incubated in digestion media (HBSS medium, 0.25 mg ml^−1^ Liberase TL (5401020001, Roche) and 0.1 mg ml^−1^ DNase I (11284932001, Sigma)) on a shaker at 37 °C for up to 45 min. The tissue was gently homogenized using a P1000 pipette every 15 min. For tissues from donor A68/770C (D99), full-thickness tissue was diced with a scalpel and digested in digestion media, as described above. Cells were pelleted and filtered through a 70-μm strainer before proceeding to Chromium 10x Genomics single cell 5′ v2 protocol as per the manufacturer’s instructions. Libraries were prepared according to the manufacturer’s protocol and sequenced on an Illumina NovaSeq 6000 S2 flow cell with 50-bp paired-end reads.

#### Control tissue from preterm infants

Uninvolved tissue from preterm infants, between 23 and 31 post-conception weeks (pcw), with necrotizing enterocolitis (NEC), focal intestinal perforation or intestinal fistula (*n* = 4) were collected at the Neonatal Department of Newcastle upon Tyne Hospitals NHS Foundation Trust with consent and ethical approval as part of the SERVIS study (REC 10/H0908/39). Tissue was resected from the infant and placed immediately into ice-cold PBS. Within 3 h, samples were enzymatically dissociated into a single-cell suspension using collagenase type IV (Worthington) for 30 min at 37 °C. Cells were filtered with 100-µm cell strainer, treated with red blood cell lysis and filtered through a 35-µm strainer. Cells were stained with DAPI before FACS sorting, selecting only for live, single cells and separating CD45-positive and CD45-negative cells. Sorted cells were then loaded onto the Chromium Controller (10x Genomics) using the Single Cell Immune Profiling kits and subsequently sequenced as per the manufacturer’s protocol.

#### Disease tissue from patients with Crohn’s disease, ulcerative colitis and coeliac disease

Crohn’s disease tissue used for validations was obtained from multiple sites. Adult Crohn’s disease surgical resections were collected from patients in the IBSEN III (Inflammatory Bowel Disease in South Eastern Norway) at Oslo University Hospital (*n* = 4) or Hospital Clinic Barcelona (*n* = 9), and biopsy material was collected from patients undergoing colonoscopy at Addenbrookes Hospital Cambridge (*n* = 4); all patients gave informed written consent. Fresh tissue was fixed in formalin and embedded in paraffin for subsequent immunostaining. Ulcerative colitis tissue was also collected from Hospital Clinic Barcelona (*n* = 3) during colonic resections, with the same consent and tissue processing procedure. Coeliac disease tissue was obtained from Oslo University Hospital (*n* = 2) or the Oxford University Hospitals NHS Foundation Trust (OUHFT) coeliac disease clinic (*n* = 2 treated coeliac, *n* = 3 untreated coeliac). As controls, healthy tissue was also collected at Oslo University Hospital from the proximal duodenum (during pancreaticoduodenectomy for patients with pancreatic cancer, *n* = 2) and the terminal ileum (*n* = 4).

Duodenal biopsies from Oslo University Hospital were collected from newly diagnosed untreated patients with coeliac disease (*n* = 2) and subsequently fixed in formalin and embedded in paraffin for immunostaining. Mucosal pinch biopsies from the second part of the duodenum from the OUHFT were obtained during gastroscopy of untreated patients with coeliac disease (*n* = 3) and treated patients with coeliac on a gluten-free diet (*n* = 2). Equivalent healthy control samples from the OUHFT (*n* = 3) were obtained from patients undergoing gastroscopy with gastrointestinal symptoms without coeliac disease. Biopsies were stored in MACS tissue storage solution (Miltenyi Biotec) before cryopreservation in freezing medium (Cryostor Cs10, Sigma-Aldrich). Samples were later recovered by thawing in a 37 °C water bath and washed in 20 ml R10 (90% RPMI (Sigma-Aldrich) and 10% FBS) before tissue dissociation. Epithelial cells were isolated using v1.11 of the published protocol (10.17504/protocols.io.bcb6isre)^69^. After isolation, epithelial cells proceeded to single-cell sequencing (10x Genomics Next GEM 5′ v1.1) as per the manufacturer’s protocol. Details of samples and metadata are available in Supplementary Table 4.

#### Ethical approval for collection of disease tissue

Tissue collected at Oslo University Hospital was approved by the Regional Committee for Medical Research Ethics (REK 20521/6544, REK 2015/946 and REK 2018/703, Health Region South-East, Norway) and comply with the Declaration of Helsinki. Tissue collected at Hospital Clinic Barcelona was approved by the Ethics Committee of Hospital Clinic Barcelona (HCB/2016/0389). Tissue from Addenbrookes Hospital was collected through the Addenbrookes–Human Research Tissue Bank HTA research licence no: 12315 (Cambridge University Hospitals Trust). Tissue collected at the OUHFT was collected under the Oxford Gastrointestinal Illnesses Biobank (REC 21/TH/0206).

### Single-molecule fluorescence in situ hybridization

Intestinal tissue was embedded in OCT and frozen on an isopentane-dry ice slurry at −60 °C, and then cryosectioned onto SuperFrost Plus slides at a thickness of 10 μm. Before staining, tissue sections were post-fixed in 4% paraformaldehyde in PBS for 15 min at 4 °C, then dehydrated through a series of 50%, 70% and 100% ethanol, for 5 min each. Staining with the RNAscope Multiplex Fluorescent Reagent Kit v2 Assay (Bio-Techne, Advanced Cell Diagnostics) was automated using a Leica BOND RX, according to the manufacturers’ instructions. After manual pre-treatment, automated processing included epitope retrieval by protease digestion with Protease IV for 30 min before RNAscope probe hybridization and channel development with Opal 520, Opal 570 and Opal 650 dyes (Akoya Biosciences). Stained sections were imaged with a Perkin Elmer Opera Phenix High-Content Screening System, in confocal mode with 1-μm *z*-step size, using a 20× water-immersion objective (NA 0.16, 0.299 μm per pixel). Channels were: DAPI (excitation 375 nm, emission 435–480 nm), Opal 520 (excitation 488 nm, emission 500–550 nm), Opal 570 (excitation 561 nm, emission 570–630 nm) and Opal 650 (excitation 640 nm, emission 650–760 nm). The fourth channel was developed using TSA-biotin (TSA Plus Biotin Kit, Perkin Elmer) and streptavidin-conjugated Atto 425 (Sigma-Aldrich).

### Immunohistochemistry

For samples collected at Oslo University Hospital, sections of formalin-fixed, paraffin-embedded tissue were cut in series at 4 µm and mounted on Superfrost Plus object glasses (Thermo Fisher Scientific). Haematoxylin–eosin staining was performed on the first sections and reviewed by an expert pathologist (F.L.J.) and the following sections were used for immunohistochemical studies. AB-PAS staining was performed by dewaxing formalin-fixed, paraffin-embedded samples and staining with Alcian blue (8GX) (AB) at pH 2.5 for acidic mucins and periodic acid-Schiff reagent (PAS) staining for neutral mucins, as previously described^70^.

Multiplex immunostaining was performed sequentially using a Ventana Discovery Ultra automated slide stainer (Ventana Medical System, 750-601, Roche). After deparaffinization of the sections, heat-induced epitope retrieval was performed by boiling the sections for 48 min with cell conditioning 1 buffer (DISC CC1 RUO, 6414575001, Roche) followed by incubation with DISC inhibitor (7017944001, Roche) for 8 min. The following primary antibodies were used: anti-human MUC6 clone CLH5 dilution 1:400 (RA0224-C.1, Scytek), anti-human MUC5AC clone CLH2 dilution 1:100 (MAB2011, Sigma), anti-human CD3 rabbit polyclonal dilution 1:50 (A0452, Dako), anti-human CD8 clone 4B11 dilution 1:30 (MA1-80231, Leica Biosystems, Invitrogen), anti-human CD4 clone SP35 dilution 1:30 (MA5-16338, Thermo Fisher), anti-TCRδ clone H-41 dilution 1:100 (sc-100289, Santa Cruz Biotechnology), anti-human FOXP3 clone 236A/E7 dilution 1:1,000 (NBP-43316, Novus Biologicals), anti-human HLA-DRα-chain clone TAL.1B5 dilution 1:200 (M0746, Dako), anti-human CD68 clone PG-M1 dilution 1:100 (M0876, Dako), anti-human CD20 clone L26 dilution 1:200 (M0755, Dako), anti-human TFF2 clone #366508 dilution 1:1,000 (MAB4077, RnD), anti-human TFF3 clone BSB-181 dilution 1:1,000 (BSB-3820-01, BioSB) and anti-human pan-CK clone AE1/AE3/PCK26, ready to use reagent (RTU) (Ventana Medical System, 760–2595, Roche).

Each primary antibody was diluted in antibody diluent (5266319001, Roche), incubated for 32 min and then washed in a 1× reaction buffer (Concentrate (10X), 5353955001, Roche). OmniMap anti-mouse horseradish peroxidase (HRP) RTU (5269652001, Roche) secondary antibody was incubated for 16 min followed by 12-min incubation with diluted opal fluorophores (Opal 6-Plex Detection Kit for Whole Slide Imaging formerly Opal Polaris 7 Color IHC Automated Detection Kit NEL871001KT) following the manufacturer’s instructions. After that, bound antibodies were denatured and HRP was quenched using Ribo CC solution (DISC CC2, 5266297001, Roche) and DISC inhibitor (7017944001, Roche). Sections were then counterstained with DAPI (DISC QD DAPI RUO, 5268826001, Roche) for 8 min and mounted with ProLong Glass Antifade mountant (Molecular Probes). Imaging was performed using a Vectra Polaris multispectral whole-slide scanner (PerkinElmer). Irrelevant, concentration-matched primary antibodies were used as negative controls. For some tissue sections, bound anti-CD3, anti-CD20, anti-MUC6 and anti-MUC5AC primary antibodies were detected with secondary antibodies conjugated with peroxidase, using the automated Ventana Discovery Ultra system and DAB, purple-responsive, yellow-responsive or teal-responsive chromogens (ChromoMap DAB Detection Kit, 5266645001; DISCOVERY Purple Kit, 07053983001; DISCOVERY Yellow Kit, 07698445001; and Discovery Teal-HRP detection kit) all from Ventana Medical System.

For samples collected at Hospital Clinic Barcelona, sections of formalin-fixed, paraffin-embedded tissue were cut into 3.5-µm sections. Immunohistochemistry was conducted for the following commercially available antibodies: anti-human MUC5AC (1:4,000; MAB2011, Sigma-Aldrich) and anti-human MUC6 (1:4,000; RA0224-C.1, ScyTek). Deparaffinization, rehydration and epitope retrieval of the sections were automatedly performed with PT link (Agilent) using Envision Flex Target Retrieval Solution Low pH (Dako). Samples were blocked with 20% of goat serum (Vector) in a PBS and 0.5% BSA solution. Biotinylated anti-mouse secondary antibodies were used (1:200; Vector). Positivity was detected with the DAB Substrate kit (K3468, Dako). Image acquisition was performed on a Nikon Ti microscope (Japan) using Nis-Elements Basic Research Software (v5.30.05).

### Image quantification

For quantification of T cell density in MUC6^+^ and neighbouring control epithelium, tissue sections from patients with Crohn’s disease (*n* = 5 sections, 3 donors) and patients with coeliac disease (*n* = 2 sections, 2 donors) stained with antibodies to MUC6, CD3, CD4, CD8 and TCRδ (see above) were used. Individual glands/epithelium (either MUC6^+^ or MUC6^−^) were annotated manually using PathViewer v3.4.0 freehand region-of-interest tool outlining the entire gland cross-section. We subtracted 3 × 3 pixel averages of autofluorescence measurement per channel with subtraction coefficients of: DAPI (1.5), TCRγδ (0.5), MUC6 (1.0), CD4 (0.25), CD3 (0.25) and CD8 (0.25). We next used QuPath^71^ v0.5 with the cellpose^72^ v2.2.3 extension to segment T cells with the ‘cyto2’ model from maximum projection of CD3, CD4, CD8 and TCRγδ, with DAPI as the nuclear marker, an expected median diameter of 10 μm and excluding cells with diameters of less than 5 μm. Segmented cells were thresholded for mean intensity expression of T cell markers by manual inspection with cut-offs of more than 25 (CD3), more than 20 (CD4), more than 10 (CD8) and more than 10 (TCRδ) and classified into subsets based on positive and negative marker expression as indicated. Using the centroid position of cells, we counted T cells per gland if the majority of the cell area was within the region of interest and quantified the T cell density per gland area comparing MUC6^+^ and control epithelium.

### Data curation and mapping

Datasets (Supplementary Table 1) were chosen from a literature search of scRNA-seq studies^5–7,9,19,22,23,50–67^. Studies were included when there was raw scRNA-seq data (FASTQ) from human gastrointestinal tract tissues (oral cavity (excluding tongue), salivary glands, oesophagus, stomach, and small and large intestine).

Available metadata from each sample were collated from various data repositories and harmonized for consistent nomenclature. Metadata related to sample retrieval methods, tissue processing and cell enrichment methods were retrieved from the methods section of the original study. Where possible, the suggestions of sample metadata from the Gut Cell Atlas Roadmap manuscript were considered^3^. An explanation and overview of metadata included and harmonized in the atlas are available in Supplementary Table 2.

For public datasets deposited to ArrayExpress, archived paired-end FASTQ files were downloaded from the European Nucleotide Archive (ENA) or ArrayExpress. For public datasets deposited to the Gene Expression Omnibus (GEO), if the Sequence Read Archive (SRA) archive did not contain the barcode read, URLs for the submitted 10X bam files were obtained using srapath v2.11.0. The bam files were then downloaded and converted to FASTQ files using 10x bamtofastq v1.3.2. If the SRA archive did contain the barcode read, the SRA archives were downloaded from the ENA and converted to FASTQ files using fastq-dump v2.11.0. Sample metadata were gathered from the abstracts deposited to the GEO or ArrayExpress, and supplementary files from publications.

Following the FASTQ file generation, 10X Chromium scRNA-seq experiments were processed using the STARsolo pipeline v1.0 detailed in https://github.com/cellgeni/STARsolo. In brief, STAR v2.7.9a was used. Transcriptome reference exactly matching Cell Ranger 2020-A for human was prepared as described in the 10X online protocol (https://support.10xgenomics.com/single-cell-gene-expression/software/release-notes/build#header). Automated script ‘starsolo_10x_auto.sh’ was used to automatically infer sample type (3′ or 5′, 10X kit version, among others). STARsolo command optimized to generate the results maximally similar to Cell Ranger v6 was used. To this end, the following parameters were used to specify unique molecular identifiers (UMI) collapsing, barcode collapsing and read clipping algorithms: ‘--soloUMIdedup 1MM_CR --soloCBmatchWLtype 1MM_multi_Nbase_pseudocounts --soloUMIfiltering MultiGeneUMI_CR --clipAdapterType CellRanger4 --outFilterScoreMin 30’. For cell filtering, the EmptyDrops algorithm used in Cell Ranger v4 and above was invoked using ‘--soloCellFilter EmptyDrops_CR’ options. Options ‘--soloFeatures Gene GeneFull Velocyto’ were used to generate both exon-only and full-length (pre-mRNA) gene counts, as well as RNA velocity output matrices.

Following read alignment and quantification, Cellbender v0.2.0 with default parameters was used to remove ambient RNA (soup). In cases where the model learning curve did not indicate convergence, the script was re-run with ‘--learning-rate 0.00005 --epochs 300’ parameters. For certain large datasets or datasets with low UMI counts, ‘--expected-cells’ and ‘--low-count-threshold’ parameters had to be adjusted individually for each sample.

### scAutoQC

On a per sample basis, scAutoQC calculated the following metrics: logarithmized numbers of counts per cell (log1p_n_counts), logarithmized numbers of genes per cell (log1p_n_genes) and the percentages of total genes expressed that are mitochondrial genes (percent_mito), ribosomal genes (percent_ribo), haemoglobin genes (percent_hb), within the top 50 genes expressed in a given cell (percent_top50), classified as soup by CellBender (percent_soup) and spliced genes (percent_spliced) (Extended Data Fig. 2). The dimensions of these eight metrics were reduced to generate a neighbourhood graph and UMAP for each sample, which was then clustered at low resolution; these clusters are referred to as quality control (QC) clusters. Classification of cells/droplets as passing or failing QC was then performed in a two-step process, first by classifying each cell as passing or failing QC based on four-metric parameters and thresholds set by a Gaussian mixture model (GMM). For the atlas, the number of GMM components was set to 10 for an overfit model. scAutoQC was subsequently improved to automate the best model fit between 1 and 10 components based on the Bayesian information criterion. Then, whole clusters were classified as passing QC if 50% or more of individual cells within the cluster passed QC. The benefits of the approach include the automated nature, removing most manually set thresholds and limiting hands-on analysis. Our unbiased approach exploits both the distribution of individual metrics and their correlations. Although there are some parameters that are set up-front, they only serve as guidance for the final flagging of low quality cells and are not sensitive to small changes in the starting points (for example, setting an initial per cent of mitochondrial genes to 15% or 20% is likely to flag the same clusters). An overview of the pipeline is in Extended Data Fig. 2, and the code (https://github.com/Teichlab/sctk/blob/master/sctk/_pipeline.py v0.1.1) and example workflow (https://teichlab.github.io/sctk/index.html) can be found in GitHub.

### Assembly of the healthy reference

After samples were run through scAutoQC, they were pooled and cells were flagged as failing QC, along with samples where less than 10% of cells or 100 cells total passed QC (18 samples). In total, we removed 596,449 (31.22%) low-quality cells during this initial filtering step. Cells were further filtered through automated doublet removal based on scrublet scores, removing a further 67,846 from the healthy reference (Extended Data Fig. 2). Cells from healthy/control samples were integrated using scVI^73–75^ (v0.16.4) with donorID_unified as batch key, log1p_n_counts and percent_mito as continuous covariates, cell cycle genes removed and 7,500 highly variable genes. For comparison, we integrated with Harmony^76^ (v0.1.7) and BBKNN^77^ (v1.4.1) using donorID_unified as the batch key and ran through the standard scIB benchmarking pipeline^78^ (v1.1.4), assessing batch correction metrics based on donorID_unified as batch key.

### Annotations of the healthy reference

Cells from the core atlas were grouped by Scanpy (v1.8.0) leiden clustering into seven broad lineages based on marker gene expression (annotation level 1; Extended Data Fig. 1a). Each lineage was split, and reintegrated with scVI (using the settings above but selecting for 5,000 highly variable genes with lineage-dependent gene list exclusions: cell cycle genes removed for all non-epithelial subsets, ribosomal genes removed for all epithelial subsets and variable immunoglobulin genes removed for B/B plasma cells) to annotate cells at fine resolution (annotation level 3). Mesenchymal populations were further split by developmental age group (first trimester fetal, second trimester fetal/preterm and adult/paediatric). Epithelial cells were further split by gastrointestinal region and/or developmental age group (oral all ages, oesophagus all ages, stomach all ages, small intestine first trimester fetal, small intestine second trimester fetal/preterm, small intestine adult/paediatric, large intestine first trimester, large intestine second trimester fetal/preterm, large intestine adult/paediatric). For fine-grained annotations of objects by broad compartment (and age/region if applicable), a combined approach including automated annotation with leiden clustering and marker gene analysis was used. Celltypist^53^ predicted labels were calculated for the entire core atlas using various relevant models (Cells_Intestinal_Tract v2, Immune_All_Low v2 and Pan_Fetal_Human v2 based on studies^5,15,53^) and custom-label transfer models based on intestinal^6^ and salivary gland^79^ datasets. During annotation, further doublets were manually removed based on a combinatorial approach considering factors such as coexpression of different cell-type marker genes, scrublet scores, gene counts, positioning relative to other cells and CellTypist predictions. Notebooks for all annotations are available via our GitHub (https://github.com/Teichlab/PanGIAtlas). MGN cells (MUC6^+^) in the healthy reference in the small intestine were identified in the healthy duodenum with leiden clustering resolution 0.5, and further refined to remove any residual doublets or MUC6^−^ cells by subclustering.

### Data projection and label prediction for diseased data

To include the disease data, we started from the raw data, remapped and applied scAutoQC to the disease data, ensuring that the healthy and disease references are comparable. Models for disease projection were made on the full healthy reference dataset (without doublets) using scANVI^80^ incorporating broad (level 1) annotations, based on the healthy reference scVI model. We projected disease data using scArches^81^ with the scANVI model. To annotate at fine resolution, we first predicted broad (level 1) lineages in the projected disease data using a label transfer method based on majority voting from *k*-nearest neighbour (kNN). Broad lineages were then split as for the healthy reference. For all lineages except epithelial, lineage-specific disease cells were projected onto the respective healthy reference lineage-specific latent space and fine-grained annotations predicted using the same method as for broad lineage predictions. Owing to an underrepresentation of epithelial cells, we added additional epithelial cell data from coeliac disease duodenum (unpublished data from the Klenerman laboratory (M.E.B.F., unpublished) and Crohn’s disease ileum and colon^22^, increasing the amount of diseased epithelial cells from 57,406 to 92,342 cells plus an additional 219,472 cells from healthy controls/non-inflamed tissue. These additional datasets were not remapped, instead these studies were added based on the raw counts matrix. Split epithelial cells from the original disease set (remapped data) and the additional disease sets (from count matrices) were concatenated and reduced to a common gene set of 18,485 genes. The resulting epithelial dataset was further split by region (stomach, small intestine and large intestine), prepared for projection using scANVI_prepare_anndata function (fills 0s for non-overlapping genes) and projected onto the respective healthy reference epithelial region-specific latent space embeddings.

To refine level 3 annotations on disease cells, we utilized the scArches weighted kNN uncertainty metric. We labelled cells as unknown if they had an ‘uncertainty score’ greater than the 90th quantile for each lineage. For epithelial cells, the 90th quantile was calculated separately for cancer cells and non-cancer cells to account for high uncertainty labelling of tumour cells. To refine the labels of these unknown cells, we performed leiden clustering (resolution = 1) and reassigned the label based on both majority voting of the higher certainty cells (above the cut-off) and marker genes. In stomach epithelium, there was one cluster of unknown cells, likely to be cancer cells, which could not be assigned a label and was therefore left annotated as unknown. In large intestinal epithelium, we found a cluster that corresponded to metaplastic Paneth cells (a cell type not present in the healthy reference), which were reannotated based on the distinct marker genes (Extended Data Fig. 4).

### Technical and biological variation

To determine the contribution of different metadata covariates to the integrated embedding of the healthy reference data, we performed linear regression for each latent component of the embedding with each covariate as previously described^82^. We performed the analysis per cell type based on level_1_annot (broad level) and level_2_annot (medium level) annotations, and for all ages or adult/paediatric only (excluding developing and preterm samples). It should be noted that although this analysis can be informative, many of the covariates included in our atlas are correlated, for example, specific studies with tissue processing methods, diseases, ages or organs. Therefore, multiple covariates can explain the same variance in the data.

### Differential abundance analysis

To identify differentially abundant cell populations, we used Milo^83^ (Milopy v0.0.999), which tests for differentially abundant neighbourhoods from kNN graphs. For comparisons between healthy developing (6–31 pcw, including preterm infants ex utero) and adult/paediatric gut, Milo was run separately per tissue with more than two donors for each group (stomach, duodenum, ileum and colon) using default parameters. For comparisons between organs in the healthy adult gut (18 years of age or older), Milo was run for each organ (oral mucosa, salivary gland, oesophagus, stomach, small intestine, large intestine and mesenteric lymph node) versus the others combined with the covariates of tissue_fraction and cell_fraction_unified and otherwise default parameters. For comparisons between disease and healthy adult samples, Milo was run comparing disease and controls from an individual study, rather than all disease and controls in the atlas, on the kNN graph from joint embedding, which has been shown to have greater sensitivity for detecting disease-associated cell states^84^. We focused comparing inflamed with neighbouring inflamed tissue from the Martin (2019)^6^ dataset.

### Differential gene expression analysis

Differential gene expression (DGE) analysis was performed using Scanpy rank gene groups function (Wilcoxon rank-sum test with default parameters) and/or by pseudobulking (decoupler^85^) and DESeq2 (ref. ^86^) analysis. For Scanpy DGE analysis, samples were preprocessed by downsampling to 200 cells per cell type per donor and removing ribosomal-related and mitochondrial-related genes to limit unwanted batch and technical effects. Decoupler pseudobulking (v1.5.0) was performed combining donor-cell-type combinations, summing raw counts per gene across cells for each combination. DGE analysis was then performed with DESeq2 (v1.38.0), with log_2_(fold change) (log_2_FC) shrinkage calculated using the ashr (v2.2_63) estimator. Genes were classified as differentially expressed when log_2_FC ≥ 0.5 or log_2_FC ≤ −0.5 and adjusted *P* ≤ 0.05. For comparison of metaplastic Paneth cells, INFLAREs and oral mucosa fibroblasts with healthy counterparts, minimum cells per donor-cell-type combination for pseudobulking was 2 and DESeq2 was run without covariates. For oral mucosa fibroblasts, comparison was between oral mucosa fibroblasts in healthy oral mucosa versus inflammatory fibroblasts annotated as oral mucosa fibroblasts in diseased ileum. For all other comparisons, minimum cells were 10 and study was included as a covariate, and comparison was between small intestinal cells in IBD versus healthy controls. DESeq2 run on bulk data from the GSE126299 LCM dataset compared metaplastic glands and inflamed epithelium from patients with IBD using default settings, without covariates. For gene set analysis, the output from Scanpy rank gene groups was filtered to contain genes with a minimum log fold change of 0.25 and a *P* value cut-off of 0.05. The resulting gene list was used for gene set analysis using the GSEApy (v1.0.4) enrichr function with relevant gene sets such as MSigDB, KEGG and GO Biological Process examined. Gene scores for epithelial cells were calculated using Drug2Cell^87^ score function with default parameters. Gene scores for fibroblasts were calculated using the Scanpy score_genes function with default parameters. Full gene lists used for gene scores are available in Supplementary Table 6. Odds ratio and *P* value of gene overlap for MGN and INFLARE marker genes in different gastrointestinal regions were calculated using GeneOverlap^88^ (v0.99.0), with the genomic background set to 18,485 genes as the total number of genes used in the marker gene analysis.

### Cell–cell interaction analysis

Cell–cell interaction analysis was performed using LIANA+ (v1.0.4)^89^, CellChat (v1.1.1)^90^ and CellPhoneDB v3 (statistical_method)^91^ to determine cell–cell interactions occurring in the small intestine during Crohn’s disease. Interaction analysis was performed on remapped data, to avoid loss of genes or interactions lost when merging additional count matrices (see ‘Data projection and label prediction for diseased data’ for more detail). Before analysis, data were preprocessed by downsampling to 50 cells per cell type per donor. Normalized count matrix with cell annotation metadata were processed through the standard CellChat and CellPhoneDB pipeline, with the communication probability truncated mean/threshold set to 0.1. Output of LIANA+ analysis was further analysed using NMF with ligand–receptor mean expression and considering only interactions expressed in at least 5% of cells. This analysis resulted in 10 interaction programmes by an automatic elbow selection procedure. Pathway enrichment analysis on the resultant ligand–receptor loadings was performed using decoupler’s univariate linear model method with pathway prior knowledge from PROGENy^92^; only factors in which at least one pathway was significantly enriched (false discovery rate ≤ 0.05) were included for analysis. Using the differential analysis statistics from DESeq2, as described above, we generated a list of deregulated ligand–receptor interactions in IBD versus healthy, or for INFLAREs and oral mucosa fibroblasts, comparing the disease cells to the appropriate healthy counterparts (see above).

### cNMF analysis

To identify shared activity and cell identity gene programs cells from diseased small intestine (Crohn’s disease, paediatric IBD and coeliac disease with a total of 99,465 cells), we analysed raw counts with cNMF (v1.3.4)^93^. We used the default processing and normalization of cNMF, which considers 2,000 highly variable genes along with 100 iterations of NMF. All other parameters were set at default values. We tested hyperparameter values of *K*, the number of factors, ranging in steps of 1 from 5 to 80, and picked on inspection a favourable tradeoff between factor stability and overall model error at *K* = 44. For determining consensus clusters, we excluded 6% of fitted cNMF spectra with a mean distance to kNNs above 0.3. The resulting per-cell gene program usage was compared across fine-grained cell annotations, identifying gene programs corresponding to the identity of MGN cells and other relevant cell types (goblet, stem and surface foveolar cells). To assess programs specific to health or disease, we performed analysis on all cells from small and large intestines using identical parameters, downsampled randomly to 200 cells per cell type per donor (resulting in 313,879 cells). In this case, we tested for values of *K* in steps of 2 from 10 to 80, choosing an optimal *K* = 64.

### Trajectory analysis

#### Monocle3

To infer the developmental trajectory giving rise to MGN or INFLAREs in the ileum IBD, we used monocle3 (v1.3.1)^94^ on a subset of data containing cells in the ileum from studies^5,6,22^. We performed Scanpy Louvain clustering on the original UMAP representation generated from the scANVI latent space to account for batch effects and inferred developmental trajectories along pseudotime by choosing the node assigned the highest number of epithelial stem cells as the root node. We then extracted the MGN or INFLARE-specific trajectory by selecting the nodes assigned the highest number of MGN or INFLAREs as the final nodes. Finally, we determined genes whose expression changes along pseudotime by using ‘monocle3::graph_test’, which leverages a Moran’s *I*-test considering gene expression changes within groups of *k* = 25 neighbouring cells on the principle trajectory graph.

#### Palantir

We analysed epithelial cell trajectories in the ileum from patients with IBD from studies^5,6^ by running transcriptome-based pseudotime estimation using Palantir (v1.3.1)^95^. Before running Palantir, we reintegrated the datasets using scVI with settings described above in ‘Assembly of the healthy reference’.

We used the default Palantir parameters with 500 waypoints specifying the root cell with the maximum gene score (using Scanpy rank genes function) of *LGR5*, *ASCL2*, *RGMB* and *OLFM4*. We then computed a CellRank^96^ (v2.0.1) kernel (Markov transition probability matrix) for Palantir pseudotime to allow projection of directional cell-state transitions onto the UMAP. To predict macrostates (potential terminal cell states), we ran CellRank’s Generalized Perron Cluster Analysis on the Markov matrix and then computed the fate probability for each cell under each terminal-state lineage. We calculated the top lineage driver genes along the stem → TA → INFLARE lineage using CellRank inference and generalized additive models. All corresponding visualizations were made using the plotting functions available in the CellRank package.

#### Genes2Genes trajectory alignment

We used Genes2Genes (G2G)^68^ to compare the INFLARE trajectory (stem → TA → INFLARE) in the diseased IBD to three other different trajectories: (1) the stem → MGN trajectory in the healthy duodenum, (2) the stem → enterocyte trajectory in the diseased ileum, and (3) the stem → goblet trajectory in the diseased ileum.

*Preparing trajectories for comparison*. For comparison 1, we ran scVI integration and Palantir pseudotime analysis as above for healthy small intestinal epithelial cells to facilitate reconstruction of the stem → MGN trajectory in the healthy duodenum. To be more confident, we also took only the stem and TA cells that have a pseudotime estimate less than the mean pseudotime of the INFLARE population (as there were some outlier stem/TA cells with higher pseudotime values in the INFLARE pseudotime range). For comparisons 2 and 3, we used the already estimated Palantir pseudotime. To extract lineage-specific cells with high confidence, we assessed the fate probability distribution (estimated by Palantir) for the INFLARE lineage across all the cells annotated under the non-lineage-specific cell types (that is, a negative control under the cells not annotated as either stem, TA or INFLARE), and removed the stem and TA cells if their fate probability was less than the 75th percentile of the negative control.

*Trajectory alignment*. G2G aligns genes along reference and query trajectories by running a dynamic programming algorithm that optimizes matching and mismatching of gene expression distributions between timepoints. This function formulates an alignment cost based on a minimum message length inference framework. As per the G2G workflow, we first discretized each pseudotime trajectory into interpolation timepoints at equal-length intervals based on the optimal number of bins inferred using the optbinning package. We then ran G2G (under its default settings) for each of the three trajectory comparisons to align transcription factors^97^ using log_1_p normalized gene expression and pseudotime estimates for each cell. For comparison 1, we considered 1,171 transcription factors common between the healthy and disease datasets, whereas 1,262 common transcription factors were aligned for comparisons 2 and 3. Interrogating the output of G2G alignment, we considered mismatches between trajectories when transcription factors had an alignment similarity ≤ 50% and optimal alignment cost ≥ 30 nits (in the unit of Shannon information).

### Bulk RNA-seq deconvolution

For bulk deconvolution analysis, we first downloaded published bulk RNA-seq datasets of adult IBD from the GEO database (GSE111889), paediatric IBD from the ArrayExpress database (E-MTAB-5464) and the Expression Atlas (E-GEOD-101794), The Cancer Genome Atlas colon adenocarcinoma using R package TCGAbiolinks (v2.18.0), coeliac disease data from the GEO (GSE131705 and GSE145358) and RNA-seq from laser capture microdissected pyloric metaplasia, inflamed and control epithelium (GSE126299). A single-cell reference for deconvolution analysis was then prepared by subsetting the overall object to only include cells from the small intestine in IBD and downsampling to 200 cells for each fine-grained cell-type annotation. BayesPrism^98^ (v2.0) was used for deconvolution analysis with raw counts for both single-cell and bulk RNA-seq data as inputs. Both the ‘cell-type labels’ and the ‘cell-state labels’ were set to fine-grained annotations. Ribosomal protein genes and mitochondrial genes were removed from single-cell data as they are not informative in distinguishing cell types and can be a source of large spurious variance. We also excluded genes from sex chromosomes and lowly transcribed as recommended by the BayesPrism tutorial. For further analysis, we applied a pairwise Welch *t*-test to select differentially expressed genes with the ‘pval.max’ being set to 0.05 and ‘lfc.min’ to 0.1. Finally, a prism object containing all data required for running BayesPrism was created using the new.prism() function, and the deconvolution was performed using the run.prism() function. For correlation analysis, we calculated the Pearson correlation between (1) the estimated abundance of INFLAREs and other cell types, and (2) the estimated INFLAREs abundance and gene expression in bulk RNA-seq datasets. For the later calculation, we first normalized raw counts in the expression matrix from each bulk dataset using R package DESeq2. To estimate the number of patients with INFLAREs in bulk RNA-seq data, we categorized samples by *MUC6* expression with a cut-off higher than the mean + 2× the standard deviation, stratifying patients as *MUC6*-high above this cut-off.

### Reporting summary

Further information on research design is available in the Nature Portfolio Reporting Summary linked to this article.

## Online content

Any methods, additional references, Nature Portfolio reporting summaries, source data, extended data, supplementary information, acknowledgements, peer review information; details of author contributions and competing interests; and statements of data and code availability are available at 10.1038/s41586-024-07571-1.

## Supplementary information

Supplementary Information Supplementary Notes 1–9, Supplementary References and Supplementary Figs 1–10.

Reporting Summary

Supplementary Tables Supplementary Tables 1–6.

## Data Availability

Raw sequencing data for adult samples are available through ArrayExpress with the accession number E-MTAB-14050. Published datasets are readily available to access through the GEO, ArrayExpress, European Genome-Phenome Archive, BioProject and Broad Institute Single Cell Portal with the accession numbers GSE152042, GSE188478, GSE180544, E-MTAB-11536, E-MTAB-9543, E-MTAB-9536, E-MTAB-8901, GSE159929, E-MTAB-9489, GSE121380, GSE157477, E-MTAB-8007, E-MTAB-8474, E-MTAB-8484, E-MTAB-8486, GSE167297, GSE150290, GSE114374, EGAS00001003779, E-MTAB-8410, GSE122846, PRJEB31843, GSE134809, GSE161267, GSE116222, GSE182270, GSE125970, GSE164241, E-MTAB-10187, E-MTAB-10268 and SCP1884, which are also detailed in Supplementary Table 1. Published bulk RNA-seq datasets are available through the GEO, ArrayExpress and Expression Atlas with the accession numbers GSE111889, E-MTAB-5464, E-GEOD-101794, GSE131705, GSE145358 and GSE126299. Imaging data are available for download from the European Bioinformatics Institute (EBI) BioImage Archive with the accession number S-BIAD1139. All relevant processed single-cell objects and models for use in future projects are available at https://gutcellatlas.org/pangi.html.

## Code availability

Code for scAutoQC is readily available on GitHub and installable via PyPI (https://github.com/Teichlab/sctk). Additional code including atlas assembly, annotation and downstream analyses is described in detail throughout the Methods and is available on GitHub (https://github.com/Teichlab/PanGIAtlas). All the analyses and plots have been made on standard Python (v3.8 or higher) and R (v4.0.4) environments, using the third-party libraries mentioned in the Methods; standard data and single-cell experiment data structures; and basic libraries: numpy, scipy, pandas, scikit-learn, statsmodels, python-igraph, seaborn, matplotlib and ggplot2. All imaging analyses were performed using PathViewer, QuPath, cellpose and OMERO.web.
