# Supplementary material for: Single-cell integration reveals metaplasia in inflammatory gut diseases
Source: Nature. 2024 Nov 20;635(8039):699–707. doi: 10.1038/s41586-024-07571-1 (PMC11578898; doi:10.1038/s41586-024-07571-1)
Supplement: Supplementary file 1 — Supplementary Notes 1–9, Supplementary References and Supplementary Figs 1–10. [file 41586_2024_7571_MOESM1_ESM.pdf]

nature portfolio

https://doi.org/10.1038/s41586-024-07571-1

Supplementary information

Single-cell integration reveals metaplasia in inflammatory gut diseases

In the format provided by the authors and unedited

Supplementary Notes

Supplementary Note 1 - scAutoQC, data integration and annotation

To integrate the different data sets, we harmonised metadata from each study including age, sex, fine-grained anatomical region, sampling methods such as sequencing technology, cell, and tissue fraction enrichment (Extended data 1c, Supplementary Table 2). To reduce batch effects from different genome references, sequence alignment and quality control (QC) methods, we remapped raw data and processed gene counts uniformly through a newly developed automated QC pipeline (Methods, Figure 1b, Extended data 1 and 2). Our pipeline (scAutoQC) removes the reliance on manual thresholds by considering 8 standard metrics (such as mitochondrial and ribosomal genes) in a reduced QC metric space, with cells/droplets filtered out per cluster on thresholds set by Gaussian Mixture Modelling on a per sample basis (Extended data 2a). A major advantage of our approach is the calculation of thresholds using multiple QC metrics per cell, exploiting both the distribution of individual metrics as well as correlations between them and allowing retention of cells with unique features (e.g. plasma cells with lower numbers of unique genes). 596,449 (~31%) low quality cells were filtered from the healthy reference using scAutoQC (Methods), with additional downstream filtering to remove doublets. Most cells included in the final healthy reference overlapped with the published studies, but some cells were unique to the atlas or to original studies due to varied QC methods (Extended data 1d).

Altogether, our atlas identified cells across all major lineages, highlighting rare and difficult to distinguish cell populations with representation across donors and appropriate studies (Supplementary Figure 4). For example, we identified cell types previously described in one region, but whose comprehensive distribution along the GI was not fully appreciated – for example, BEST4 cells in stomach (Supplementary Figure 2d), *MUC6*+ mucous gland neck (MGN)[1] and *MUC5AC*+ surface foveolar cells in the duodenum (Supplementary Figure 2e). Our approach uncovered rare cell types that represented less than 0.02% of the atlas, such as deep crypt secretory (DCS) *MUC17*+ cells[2] in the ceacum and large intestine (Supplementary Figure 2f), and Langerhans dendritic cells (DCs) (Supplementary Figure 1a). Finally, the integrated atlas allowed increased distinction between highly similar cell types such as IgA1 and IgA2 B plasma cells (Supplementary Figure 1d). Using scAutoQC and our integration approach, we uncovered all expected cell types across the GI, with the exception of neutrophils.

Granulocytes present a particular challenge in scRNAseq profiling, due to sensitivity to tissue dissociation methods, high RNase content and low number of expressed genes[3,4]. To identify potential neutrophils in our data, we filtered cells failing scAutoQC and with CellTypist predictions as monocyte/neutrophil related subsets. Using this approach we identified 1,893 putative neutrophils, which were mostly derived from one unpublished donor where samples were processed at 4°C (see methods for donor D105) (Supplementary Figure 5). Hence, we highlight potential solutions to capture neutrophils in future studies, and provide a reference for annotating and mapping neutrophils.

Supplementary Note 2 - Disease-specific gene expression programs

Exploring disease-specific gene expression across lineages, we performed consensus non-negative matrix factorisation (cNMF) analysis in cells from both small and large intestine across the atlas (Supplementary Figure 9a). We identified disease and cell type specific factors such as factors 27, 31, 33 and 47. Factors 27 and 47 were specific to fibroblasts from IBD and healthy controls respectively (Supplementary Figure 9a). Top genes in factor 27 included genes known to be expressed in inflammatory fibroblasts (eg. *CXCL1, 2, CCL2, IL6*) with highest scores in "oral mucosa" fibroblasts and factor 47 including genes for extracellular matrix deposition (*CD248, MFAP5, FBN1, FBLN2*) and TGFβ signalling (*CLEC3B, TGFBR3)* (Supplementary Figure 9b). Factor 31 (with high ranking of genes including *IL1B, IL1A, CCL3L1, CCL3, CXCL2, CXCL3* and *TNF*) was specific to monocytes from IBD patients, reflecting ongoing inflammation. We identified an IBD specific factor (33) in epithelial cells including *DUOX, DUOXA2, LCN2,* a gene signature that emerges over time in inflamed epithelial cells in IBD[2]. We next performed differential gene expression (DGE) analysis across all cell types to compare transcriptional changes in IBD to healthy small intestinal tissue (Supplementary Figure 9c). *DUOX2* and *FAM3D* were upregulated in inflamed enterocytes across the atlas, and in other inflamed or metaplastic epithelial cells within our atlas (Supplementary Figure 9d). Intriguingly, Tregs from inflamed tissue had ~400 fold downregulation of *PTPRCAP,* encoding the CD45 activating protein involved in lymphocyte activation, and downregulation of *TNFRSF18* (GITR), important for suppressive function[5] (Supplementary Figure 9e). Gene set enrichment analysis (GSEA) for downregulated genes highlighted that Tregs in IBD exhibit gene expression patterns consistent with lack of activation and suppressive function (Supplementary Figure 9f).

Supplementary Note 3 - Inflammatory fibroblasts in IBD

Disease-specific fibroblasts from the intestines of IBD patients phenocopy healthy oral mucosa fibroblasts. In the healthy reference, oral mucosa fibroblasts included cells from gingival mucosa, with marker genes involved in collagen and matrix deposition (*CTHRC1*, *COL12A1*, *COL1A1*, *COL5A2, CSTK*) (Supplementary Figure 1). Compared with their healthy counterparts, disease-associated oral mucosa-like fibroblasts in single cell data overexpressed marker genes of inflammatory/activated fibroblasts reported in UC and CD[6–10] (Extended data 4e). Moreover, using CellTypist models from published studies[6,7], oral mucosa fibroblasts were predicted as inflammatory/activated fibroblast populations (Extended data 4f). Hence, we refer to this disease cell population as inflammatory fibroblasts. Hierarchical clustering of oral mucosa/Inflammatory fibroblasts across locations distinguished cells from gingiva/periodontium but not buccal mucosa, potentially reflecting unique microbiome and disease susceptibilities of gingival mucosa[11,12]. Furthermore, in disease-associated inflammatory fibroblasts vs healthy oral mucosa fibroblasts, DEGs were enriched for various inflammatory pathways (such as KEGG IL-17 signalling pathway, MSigDB Hallmark Interferon gamma response, MSigDB Hallmark inflammatory response; Figure 2e, Extended data 4h-j), supporting an immune-modulating role of this population in the lower GI tract during disease.

This oral mucosa fibroblast state, which exists in homeostasis in healthy gingival mucosa, may be primed to promote inflammation and resolve infection. Gingival mucosa is particularly vulnerable to damage and infection, as the first point of contact for commensals and pathogens entering the GI tract, and due to injury exposure through mastication[13]. Thus, it is possible that a primed fibroblast state in homeostasis facilitates rapid immune and healing responses. We noted that oral mucosa fibroblasts from periodontitis expressed higher levels

of marker genes than healthy oral mucosa fibroblasts or the equivalent population in IBD (Extended data 4k, l) and had increased levels of inflammatory gene scores compared with control populations (Extended data 4l, m). Understanding the regulation of inflammatory gene expression programs in this fibroblast population could further our understanding of their pathogenic role in disease.

Supplementary Note 4

Identification of metaplastic *MUC6*+ cells in single cell data

In healthy tissue, Brunner's glands reside primarily in the submucosal layer of the proximal duodenum and function to guard the epithelium by secreting gel-forming mucins (including *MUC6* and *MUC5AC*), and factors involved in immune defence, pH regulation and cell proliferation and differentiation[14]. These cells are highly abundant in the healthy stomach epithelium, with surface foveolar (SF) cells (*MUC5AC* expressing, also known as pit or surface mucous cells) residing at the top of the glands and mucous gland neck cells (*MUC6* expressing) residing in the lower half of the pyloric gland, with similar mucous-secreting and barrier functions[15,16] (Figure 3g). Importantly, these cells are absent in healthy jejunum, ileum or large intestine (Figure 3g, h) and their presence (usually identified histologically via pyloric gland morphology in H&E or MUC6/MUC5AC IHC) indicates pyloric metaplasia. In our atlas, the original published annotations of *MUC6*-expressing cells in disease were a mixture of cell types including microfold cells, *OLFM4*+ stem cells and goblet cells, or these cells were excluded from original studies entirely (Supplementary Figure 10).

To explore the hypothesis that *MUC6*-expressing cells represent pyloric metaplasia (ie. pyloric and Brunner's gland-like appearance), we examined sequencing metadata and compared our data to previous studies. These cells had similar QC metric distributions to other epithelial cells, indicating that they were not a result of technical artefacts. In addition, they were more frequent in resection rather than biopsy samples, which may reflect the larger tissue areas captured in resection samples and/or disease severity of patients requiring resection (Extended data 6g, h). Pyloric metaplasia has also been reported in the large intestine of IBD patients, albeit less frequently (Supplementary Table 3). Therefore we mapped additional epithelial cells from CD and UC samples from 3 published studies (209,347 cells from 23 control, 24 CD and 23 UC patients)[9,17,18] to our healthy reference to identify potential *MUC6*-expressing cells (Extended data 6i-k). We found only a small number of cells expressing *MUC6, PGC, AQP5* and *BPIFB1,* mostly coming from one control sample (a deceased organ donor[6]), and thus could not confidently identify MGN/INFLARE cells in available data from the large intestine. Larger or stratified patient cohorts with histologically identified pyloric metaplasia are needed to profile INFLAREs from the large intestine by scRNAseq.

Supplementary note 5 - Surface foveolar-like cells

In contrast to healthy duodenum and stomach, the disease cells labelled as SF cells did not express high levels of *MUC5AC* (Extended data 6b). Thus, despite the automated label, it is unlikely that these cells are true metaplastic SF cells. This population was distinct from other epithelial cells in the atlas (Extended data 6c), had low uncertainty score for label transfer (Supplementary Figure 7i) and had transcriptional similarity to healthy stomach and duodenum SF cells (Extended data 6e, f). Intriguingly, known epithelial inflammatory genes (*DUOX2, LCN2 and DUOXA2*) are expressed in SF-like cells and overlap with markers of healthy stomach SF cells, suggesting that this disease-specific epithelial gene signature may exist in

the healthy stomach (Extended data 6f). Overall, we were unable to identify metaplastic SF cells in the atlas and dedicated studies capturing these cells by scRNAseq could provide further insights into their role(s) in disease.

Supplementary note 6 - INFLARE validation in bulk RNAseq data

To estimate the percentage of CD patients with INFLAREs, we stratified healthy controls and CD patients from bulk datasets as high or low *MUC6* (Methods). Across studies, the percentage of *MUC6*-high CD patients was ~29%, consistent with previous histological reports (Extended data 7b). *MUC5AC* gene expression in bulk data from datasets was similarly increased in CD and UC patients, further supporting the presence of pyloric metaplasia in disease datasets (Extended data 7c). Deconvolution of published data from laser capture microdissected (LCM) pyloric metaplasia confirmed a high proportion of INFLAREs, further supporting these cells as *MUC6*+ metaplastic cells (Figure 3e). Differential expression of metaplastic glands versus inflamed crypts from IBD patients revealed upregulation of INFLARE marker genes such as *MUC6* and *BPIFB1* (Extended data 7d). In duodenal samples from celiac disease, proportions of INFLAREs were higher, albeit not significantly, when compared to healthy duodenum (Extended data 7e).

In CRC TCGA data, INFLARE proportions were elevated compared to healthy controls, and particularly elevated in microsatellite unstable instability (MSI)-high cancers, which display higher mutational burden and increased infiltration of immune cells[19] (Extended data 7f). Intriguingly, there is a potential link between INFLAREs and CRC. We observed INFLAREs in tissue sections and bulk RNAseq data of UC patients, who have an increased risk of CRC[20]. Bulk deconvolution of TCGA data suggested that INFLAREs are present in colon adenocarcinoma, particularly in MSI-high tumours. In two independent studies, MUC6 expression in the colon of UC patients was significantly associated with neoplasms, suggesting that INFLAREs may play a direct role in colitis-associated CRC[21,22]. These results are consistent with recent identification of gastric metaplasia related gene expression (including *TFF2, AQP5* along with reduced *CDX2*) in serrated polyps, which are pre-cancerous lesions associated with MSI high CRC[23].

Supplementary Note 7 - TFs altered in INFLARE versus control trajectories

To further investigate gene expression changes that drive the formation of INFLAREs cells, we used Genes2Genes to compare the trajectories of INFLAREs to healthy MGN cells or inflamed enterocytes or goblet cells (Figure 4d, Extended data 8c-e). These three comparisons revealed 19 common significantly mismatched TFs: *DACH1, EGR1, ETS1, FLYWCH1, FOSB, HES1, HOXB9, JUNB, MAFF, MYRFL, NR4A1, PITX2, PLAGL1, SPDEF, ZFPM1, ZNF236, ZNF629, ZNF814* and *ZNF90*. These TFs have been implicated in regulating stemness, development and secretory programmes (*DACH1, HES1, PITX2, SPDEF)*, epithelial injury responses (*EGR1, FOSB, JUNB, ETS1*), and metaplasia in the stomach and pancreas (*HES1, SPDEF)*. DACH1 is involved in cell fate determination and promotes organoid formation, stem cell proliferation and maintenance[24]. EGR1, FOSB and JUNB are involved in cell survival and growth, with EGR1/FOSB differentially expressed in young vs old intestinal stem cells and promoting growth of intestinal organoids[25,26]. In a study of colonic organoid cytokine exposure, ETS1 was the only TF found to be regulated by more

than two IBD relevant cytokines (TNFα, IFNɣ and IL13)[27]. Expression of ETS1 is predictive of anti-TNFα responses and confers susceptibility to IBD[28–30]. HES1, a Notch signalling target upregulated in INFLAREs toward the end of the trajectories, is expressed in gastric epithelial cells and implicated in metaplasia of the oesophagus and pancreas[31–34]. PITX2, a gene involved in left-right asymmetry in development, is downregulated in IBD[35,36] but upregulated in CRC. Intriguingly we discuss the role of LEFTY1 (Supplementary Note 6), another gene involved in left-right asymmetry, in intestinal metaplasia[37,38]. In our atlas, we find an upregulation of LEFTY1 in inflamed stem cells (Figure 4g), together suggesting that developmental programs may be reactivated during epithelial cell metaplasia. SPDEF is involved in secretory programs and maturation of intestinal goblet and Paneth cells[39,40] and has been linked to mucous cell metaplasia in the airways, pancreas and stomach[41,42]. SPDEF has also been implicated in transcriptional activation of NR4A1[43], another mismatched TF, suggesting that there could be interplay between mismatched TFs in addition to their target genes. In conclusion, the TFs differentially expressed along the INFLARE trajectory have been implicated in processes related to mucinous epithelial cell metaplasia.

Supplementary Note 8 - Comparison of inflamed and healthy stem cells

Our cNMF analysis showed LGR5+ stem cells and MGN/INFLAREs both expressed LEFTY1, which is also expressed in undifferentiated progenitor populations of intestinal metaplasia in the oesophagus (Barrett's oesophagus)[37] and in stomach[38]. In the stomach, the *LEFTY1+* progenitors represent a small subcluster of MGN cells in healthy stomach mucosa, referred to as "linking" stem cells expressing *LEFTY1* and *OLFM4*. In mammary gland epithelial cells, LEFTY1 was shown to regulate self-renewal and drive proliferation in breast tumorigenesis[44]. In our atlas, we found significantly upregulated *LEFTY1* expression in inflamed intestinal stem cells compared to controls (Figure 4g, Extended data 8i). *LEFTY1* was also expressed in INFLAREs, but the expression was dataset dependent and needs further validation (Extended data 8i). Other stem cell marker genes (*REG1A, OLFM4, SLC12A2)* were also upregulated in IBD (Extended data 8j). In addition, stem cells from inflamed tissue had enhanced expression of interferon stimulated genes such as *IFI27*, MHC class II antigen presentation genes (*HLA-DRA, HLA-DRB1*) and the transcription factor (TF) STAT1, which is implicated in interferon induced MHC-II expression (Extended data 8j). Intriguingly, MHC-II expression in murine *Lgr5+* intestinal stem cells is important for epithelial cell remodelling during infection, through direct interaction with CD4+ T helper cells[45]. In addition, STAT1 has recently been implicated in regulating hematopoietic stem cell maintenance and self-renewal[46]. Overall, we see substantial expression differences in stem cell associated genes between normal and inflamed gut. These differences could result in altered cell fates in inflamed gut, ultimately leading to the emergence of metaplastic cells.

Supplementary Note 9 - Intercellular networks

To identify potential intercellular networks impacting on stem cell and INFLARE trajectories and function, we performed cell-cell communication analysis with LIANA+[47], which combines different ligand-receptor databases and analysis methods. Using the output of LIANA+ for NMF analysis, we identified a factor representing signalling from fibroblast subsets to stem

cells and INFLAREs (Extended data 8k, l). Predicted interactions among these cell types included the ligands *NGR1, AREG* and *EREG*, which were upregulated in oral mucosa/inflammatory fibroblasts and potentially signal to stem cells and INFLAREs via *EGFR/ERBB2/ERBB3* (Extended data 8m). All three ligands have been implicated in maintaining the intestinal stem cell niche and promoting regeneration and barrier function[48–52]. *ERBB3* is expressed in intestinal metaplasia of the stomach and is hypothesised to interact with fibroblasts during epithelial injury via *NRG1* for tissue regeneration leading to metaplasia[38,53]. Similar interactions and mechanisms could be involved in pyloric metaplasia, however further experimentation is needed to confirm this.

References

1. Hickey, J. W. *et al.* Organization of the human intestine at single-cell resolution. *Nature* **619**, 572–584 (2023).
2. Nie, H. *et al.* Single-cell meta-analysis of inflammatory bowel disease with scIBD. *Nature Computational Science* **3**, 522–531 (2023).
3. Kim, N., Kang, H., Jo, A., Yoo, S.-A. & Lee, H.-O. Perspectives on single-nucleus RNA sequencing in different cell types and tissues. *J Pathol Transl Med* **57**, 52–59 (2023).
4. Wigerblad, G. *et al.* Single-Cell Analysis Reveals the Range of Transcriptional States of Circulating Human Neutrophils. *J. Immunol.* **209**, 772–782 (2022).
5. Tian, J., Zhang, B., Rui, K. & Wang, S. The Role of GITR/GITRL Interaction in Autoimmune Diseases. *Front. Immunol.* **11**, 588682 (2020).
6. Elmentaite, R. *et al.* Cells of the human intestinal tract mapped across space and time. *Nature* **597**, 250–255 (2021).
7. Martin, J. C. *et al.* Single-Cell Analysis of Crohn's Disease Lesions Identifies a Pathogenic Cellular Module Associated with Resistance to Anti-TNF Therapy. *Cell* **178**, 1493–1508.e20 (2019).
8. Kinchen, J. *et al.* Structural Remodeling of the Human Colonic Mesenchyme in Inflammatory Bowel Disease. *Cell* **175**, 372–386.e17 (2018).
9. Smillie, C. S. *et al.* Intra- and Inter-cellular Rewiring of the Human Colon during Ulcerative Colitis. *Cell* **178**, 714–730.e22 (2019).
10. Huang, B. *et al.* Mucosal Profiling of Pediatric-Onset Colitis and IBD Reveals Common Pathogenics and Therapeutic Pathways. *Cell* **179**, 1160–1176.e24 (2019).

11. Moutsopoulos, N. M. & Moutsopoulos, H. M. The oral mucosa: A barrier site participating in tissue-specific and systemic immunity. *Oral Dis.* **24**, 22–25 (2018).

12. Proctor, D. M. & Relman, D. A. The Landscape Ecology and Microbiota of the Human Nose, Mouth, and Throat. *Cell Host Microbe* **21**, 421–432 (2017).

13. Waasdorp, M. *et al.* The Bigger Picture: Why Oral Mucosa Heals Better Than Skin. *Biomolecules* **11**, (2021).

14. Krause, W. J. Brunner's glands: a structural, histochemical and pathological profile. *Prog. Histochem. Cytochem.* **35**, 259–367 (2000).

15. Hanby, A. M., Poulsom, R., Playford, R. J. & Wright, N. A. The mucous neck cell in the human gastric corpus: a distinctive, functional cell lineage. *J. Pathol.* **187**, 331–337 (1999).

16. Willet, S. G. & Mills, J. C. Stomach Organ and Cell Lineage Differentiation: from Embryogenesis to Adult Homeostasis. *Cell Mol Gastroenterol Hepatol* **2**, 546–559 (2016).

17. Kong, L. *et al.* The landscape of immune dysregulation in Crohn's disease revealed through single-cell transcriptomic profiling in the ileum and colon. *Immunity* **56**, 444–458.e5 (2023).

18. Garrido-Trigo, A. *et al.* Macrophage and neutrophil heterogeneity at single-cell spatial resolution in human inflammatory bowel disease. *Nat. Commun.* **14**, 4506 (2023).

19. Mlecnik, B. *et al.* Integrative Analyses of Colorectal Cancer Show Immunoscore Is a Stronger Predictor of Patient Survival Than Microsatellite Instability. *Immunity* **44**, 698–711 (2016).

20. Lakatos, P.-L. & Lakatos, L. Risk for colorectal cancer in ulcerative colitis: changes, causes and management strategies. *World J. Gastroenterol.* **14**, 3937–3947 (2008).

21. Borralho, P., Vieira, A., Freitas, J., Chaves, P. & Soares, J. Aberrant gastric apomucin expression in ulcerative colitis and associated neoplasia. *J. Crohns. Colitis* **1**, 35–40 (2007).

22. Tatsumi, N. *et al.* Cytokeratin 7/20 and mucin core protein expression in ulcerative colitis-associated colorectal neoplasms. *Virchows Arch.* **448**, 756–762 (2006).

23. Chen, B. *et al.* Differential pre-malignant programs and microenvironment chart distinct

paths to malignancy in human colorectal polyps. *Cell* **184**, 6262–6280.e26 (2021).

24. Hu, X. *et al.* Organoid modelling identifies that DACH1 functions as a tumour promoter in colorectal cancer by modulating BMP signalling. *EBioMedicine* **56**, 102800 (2020).
25. Nefzger, C. M. *et al.* Intestinal stem cell aging signature reveals a reprogramming strategy to enhance regenerative potential. *NPJ Regen Med* **7**, 31 (2022).
26. Vickers, E. R. *et al.* Ternary complex factor-serum response factor complex-regulated gene activity is required for cellular proliferation and inhibition of apoptotic cell death. *Mol. Cell. Biol.* **24**, 10340–10351 (2004).
27. Pavlidis, P. *et al.* Cytokine responsive networks in human colonic epithelial organoids unveil a molecular classification of inflammatory bowel disease. *Cell Rep.* **40**, 111439 (2022).
28. de Lange, K. M. *et al.* Genome-wide association study implicates immune activation of multiple integrin genes in inflammatory bowel disease. *Nat. Genet.* **49**, 256–261 (2017).
29. Ellinghaus, D. *et al.* Analysis of five chronic inflammatory diseases identifies 27 new associations and highlights disease-specific patterns at shared loci. *Nat. Genet.* **48**, 510–518 (2016).
30. Reshef, Y. A. *et al.* Detecting genome-wide directional effects of transcription factor binding on polygenic disease risk. *Nat. Genet.* **50**, 1483–1493 (2018).
31. Sekine, A., Akiyama, Y., Yanagihara, K. & Yuasa, Y. Hath1 up-regulates gastric mucin gene expression in gastric cells. *Biochem. Biophys. Res. Commun.* **344**, 1166–1171 (2006).
32. Chen, X. *et al.* Aberrant expression of Wnt and Notch signal pathways in Barrett's esophagus. *Clin. Res. Hepatol. Gastroenterol.* **36**, 473–483 (2012).
33. Nishikawa, Y. *et al.* Hes1 plays an essential role in Kras-driven pancreatic tumorigenesis. *Oncogene* **38**, 4283–4296 (2019).
34. Hidalgo-Sastre, A. *et al.* Hes1 Controls Exocrine Cell Plasticity and Restricts Development of Pancreatic Ductal Adenocarcinoma in a Mouse Model. *Am. J. Pathol.* **186**, 2934–2944 (2016).
35. Sæterstad, S. *et al.* Profound gene expression changes in the epithelial monolayer of active ulcerative colitis and Crohn's disease. *PLoS One* **17**, e0265189 (2022).

36. Ouahed, J. *et al.* Mucosal Gene Expression in Pediatric and Adult Patients With Ulcerative Colitis Permits Modeling of Ideal Biopsy Collection Strategy for Transcriptomic Analysis. *Inflamm. Bowel Dis.* **24**, 2565–2578 (2018).
37. Nowicki-Osuch, K. *et al.* Molecular phenotyping reveals the identity of Barrett's esophagus and its malignant transition. *Science* vol. 373 760–767 Preprint at https://doi.org/10.1126/science.abd1449 (2021).
38. Tsubosaka, A. *et al.* Stomach encyclopedia: Combined single-cell and spatial transcriptomics reveal cell diversity and homeostatic regulation of human stomach. *Cell Rep.* **42**, 113236 (2023).
39. Gregorieff, A. *et al.* The ets-domain transcription factor Spdef promotes maturation of goblet and paneth cells in the intestinal epithelium. *Gastroenterology* **137**, 1333–45.e1–3 (2009).
40. Noah, T. K., Kazanjian, A., Whitsett, J. & Shroyer, N. F. SAM pointed domain ETS factor (SPDEF) regulates terminal differentiation and maturation of intestinal goblet cells. *Exp. Cell Res.* **316**, 452–465 (2010).
41. Ma, Z. *et al.* Single-Cell Transcriptomics Reveals a Conserved Metaplasia Program in Pancreatic Injury. *Gastroenterology* **162**, 604–620.e20 (2022).
42. Curran, D. R. & Cohn, L. Advances in mucous cell metaplasia: a plug for mucus as a therapeutic focus in chronic airway disease. *Am. J. Respir. Cell Mol. Biol.* **42**, 268–275 (2010).
43. Wang, Y. *et al.* SPDEF suppresses head and neck squamous cell carcinoma progression by transcriptionally activating NR4A1. *Int. J. Oral Sci.* **13**, 33 (2021).
44. Zabala, M. *et al.* LEFTY1 Is a Dual-SMAD Inhibitor that Promotes Mammary Progenitor Growth and Tumorigenesis. *Cell Stem Cell* **27**, 284–299.e8 (2020).
45. Biton, M. *et al.* T Helper Cell Cytokines Modulate Intestinal Stem Cell Renewal and Differentiation. *Cell* **175**, 1307–1320.e22 (2018).
46. Li, J. *et al.* STAT1 is essential for HSC function and maintains MHCIIhi stem cells that resist myeloablation and neoplastic expansion. *Blood* **140**, 1592–1606 (2022).
47. Dimitrov, D. *et al.* LIANA+: an all-in-one cell-cell communication framework. *bioRxiv* 2023.08.19.553863 (2023) doi:10.1101/2023.08.19.553863.

48. Shao, J. & Sheng, H. Amphiregulin promotes intestinal epithelial regeneration: roles of intestinal subepithelial myofibroblasts. *Endocrinology* **151**, 3728–3737 (2010).
49. Jardé, T. *et al.* Mesenchymal Niche-Derived Neuregulin-1 Drives Intestinal Stem Cell Proliferation and Regeneration of Damaged Epithelium. *Cell Stem Cell* **27**, 646–662.e7 (2020).
50. Chen, F. *et al.* Neutrophils Promote Amphiregulin Production in Intestinal Epithelial Cells through TGF-β and Contribute to Intestinal Homeostasis. *J. Immunol.* **201**, 2492–2501 (2018).
51. Childs, C. J. *et al.* EPIREGULIN creates a developmental niche for spatially organized human intestinal enteroids. *JCI Insight* **8**, (2023).
52. Holloway, E. M. *et al.* Mapping Development of the Human Intestinal Niche at Single-Cell Resolution. *Cell Stem Cell* **28**, 568–580.e4 (2021).
53. Huang, K. K. *et al.* Spatiotemporal genomic profiling of intestinal metaplasia reveals clonal dynamics of gastric cancer progression. *Cancer Cell* **41**, 2019–2037.e8 (2023).

Supplementary Figures

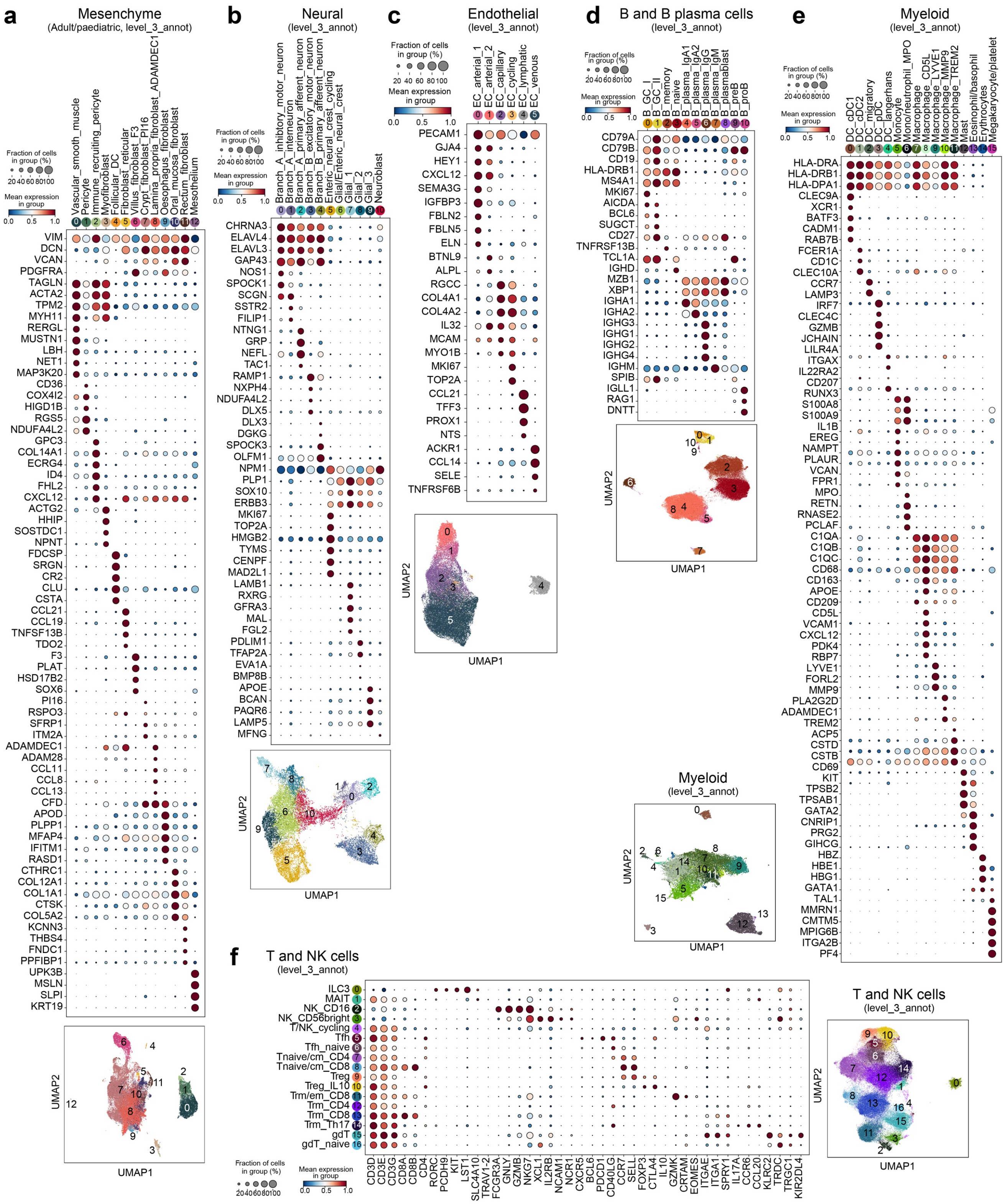

Supplementary Figure 1: Fine-grained annotations with marker dot plot and UMAP of cells from non-epithelial lineages. a) Mesenchymal lineage annotations for cells in adult/pediatric samples. b) Neural lineage annotations. c) Endothelial lineage annotations. d) B and B plasma lineage annotations. e) Myeloid lineage annotations. f) T and NK lineage annotations.

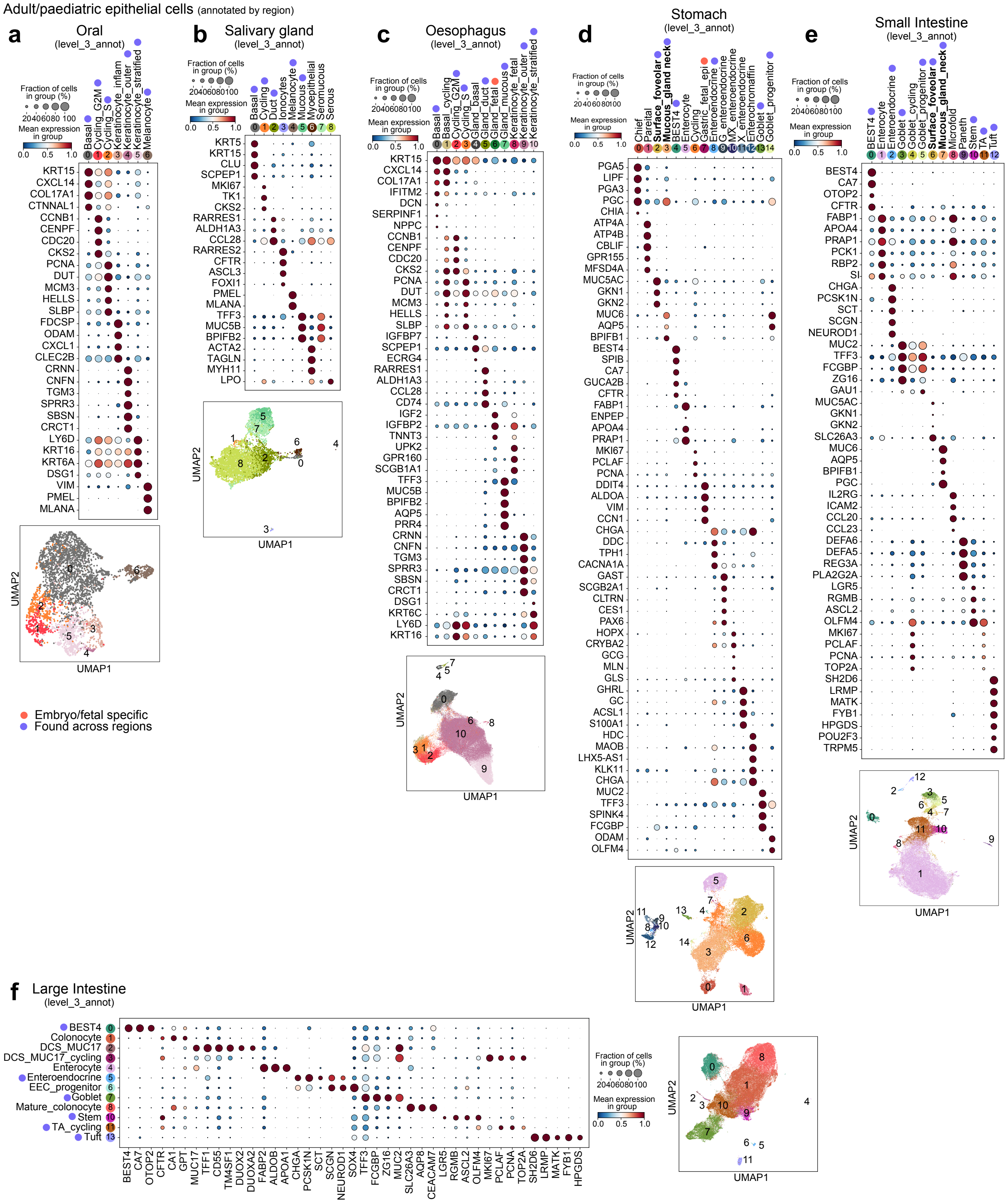

Supplementary Figure 2: Fine-grained annotations with marker dot plot and UMAP of cells from adult/pediatric epithelial lineages, subclustered by organs. a) Oral mucosa epithelial cells (periodontium, gingival and buccal mucosa). b) Salivary gland epithelial cells. c) Oesophagus epithelial cells. d) Stomach epithelial cells. e) Small intestine epithelial cells (duodenum, jejunum, ileum). f) Large intestine epithelial cells (appendix, ceacum, ascending/descending/transverse/sigmoid colon, rectum). Epithelial cells found only in embryonic/fetal samples are highlighted with an orange dot, those found across regions with a purple dot.

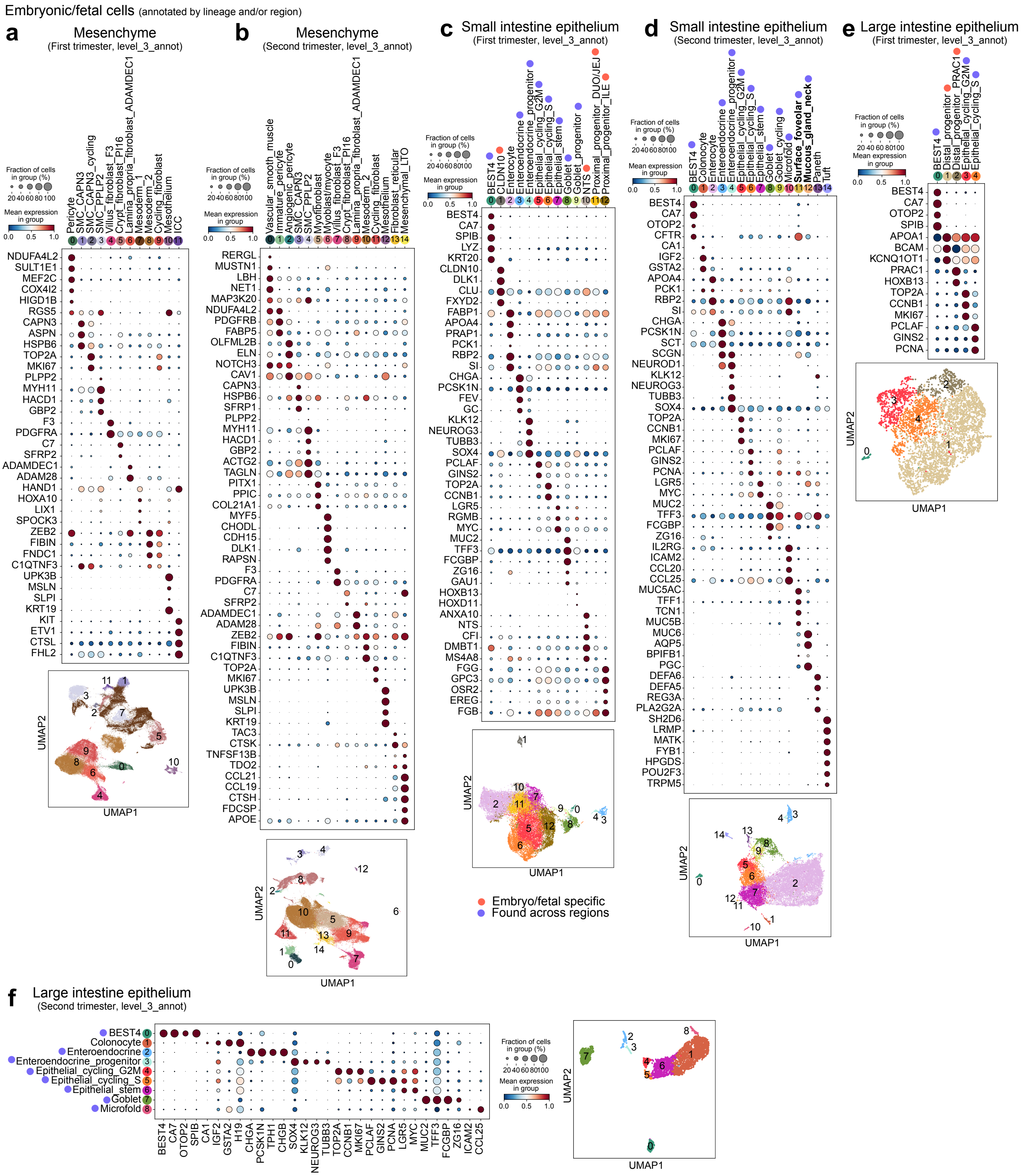

Supplementary Figure 3: Fine-grained annotations with marker dot plot and UMAP of mesenchymal and small/large intestine epithelial cells from embryo, fetal and preterm samples. a) Mesenchymal cells from first trimester samples. b) Mesenchymal cells from second trimester second trimester and preterm samples. c) Small intestine epithelial cells from first trimester samples. d) Small intestine epithelial cell second trimester and preterm samples. e) Large intestine epithelial cells from first trimester samples. f) Large intestine epithelial cell second trimester and preterm samples. Epithelial cells found only in embryonic/fetal samples are highlighted with an orange dot, those found across regions with a purple dot.

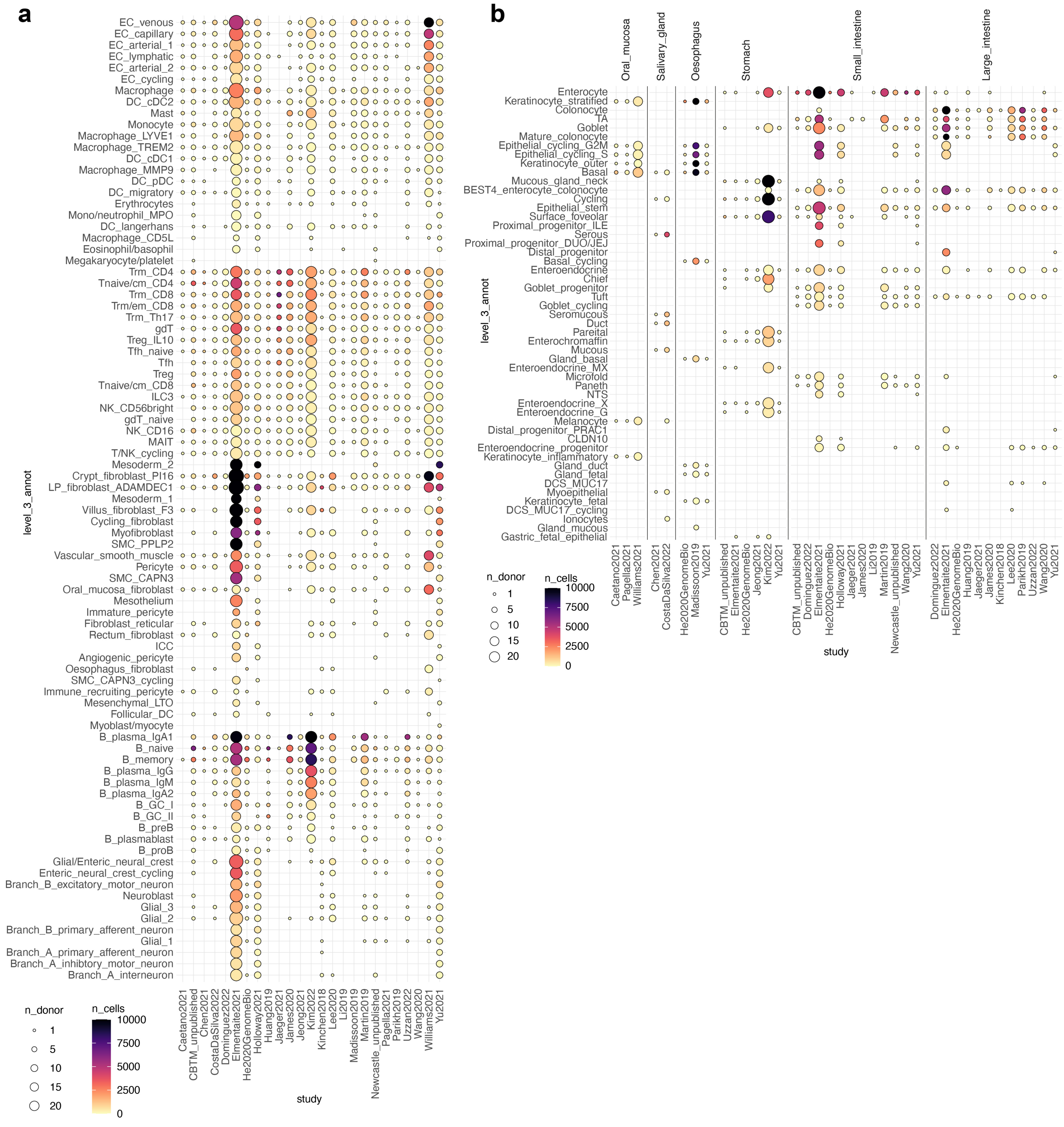

Supplementary Figure 4: Number of cells and donors per cell type across studies for (a) all non-epithelial subsets and (b) epithelial cells by GI region. Plots show representation of cell types from at least two donors and across appropriate studies.

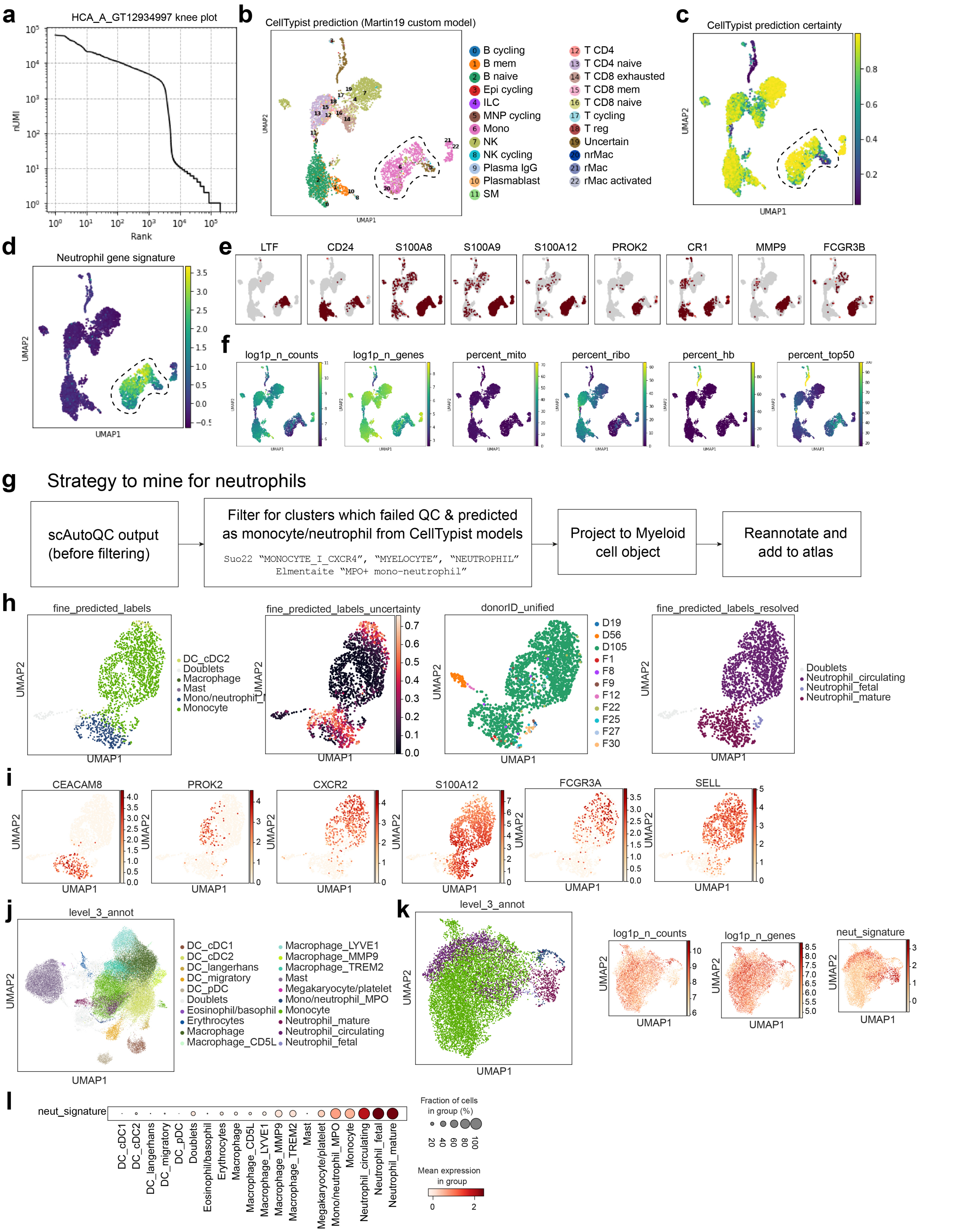

Supplementary Figure 5: Strategy to find neutrophils in the atlas. a) Barcode rank plot of a sample with neutrophils, showing a "knee" with sudden drop in number of UMIs characteristic of neutrophils. b, c) UMAPs of celltypist predicted labels and label certainty for the sample in (a) showing a distinct cluster predicted as monocytes and uncertain cells. d) UMAP as in (b) and (c) showing high scoring for neutrophil genes (shown individually in e) in the potential neutrophil cluster. e) Expression of neutrophil marker genes used for neutrophil genes score in (d). f) UMAP of QC metrics for the same sample (mito = mitochondrial, ribo = ribosomal, hb = haemoglobin). g) Overview of the final strategy used to identify neutrophils in the atlas, based cells based on failed QC status and CellTypist predictions as subsets related to neutrophils. Using this method, 1,893 cells were identified in the healthy reference and 0 cells in the extended atlas. h) UMAP of the 1,893 potential neutrophils in the atlas showing predicted labels as monocytes, the uncertainty score for label transfer, donors and

annotated labels. Donors beginning with F are from fetal samples. Most neutrophils come from donor D105 where samples were processed mainly at 4 °C (see Methods), a more gentle tissue processing that seemingly resulted in greater capture of neutrophils. Mature neutrophils are defined by expression of *CEACAM8* and *S100A12*, representing neutrophils newly matured from bone marrow precursors and circulating neutrophils express *CXCR2, S100A12, FCGR3A* and *SELL*, representing classical circulating neutrophils potentially coming from blood contamination in the sample rather than tissue resident neutrophils. i) UMAPs of marker gene expression from cells in (h). j) UMAP showing neutrophils mapped back to healthy and disease myeloid cells in the atlas. k) UMAP of subclustered neutrophil and monocyte subsets, although some overlapping between circulating neutrophils and monocytes occurs (also observed in (j)), cells annotated as neutrophils have lower numbers of counts and genes, and higher neutrophil signature than monocytes. l) Dotplot showing expression of the neutrophil signature across myeloid subsets.

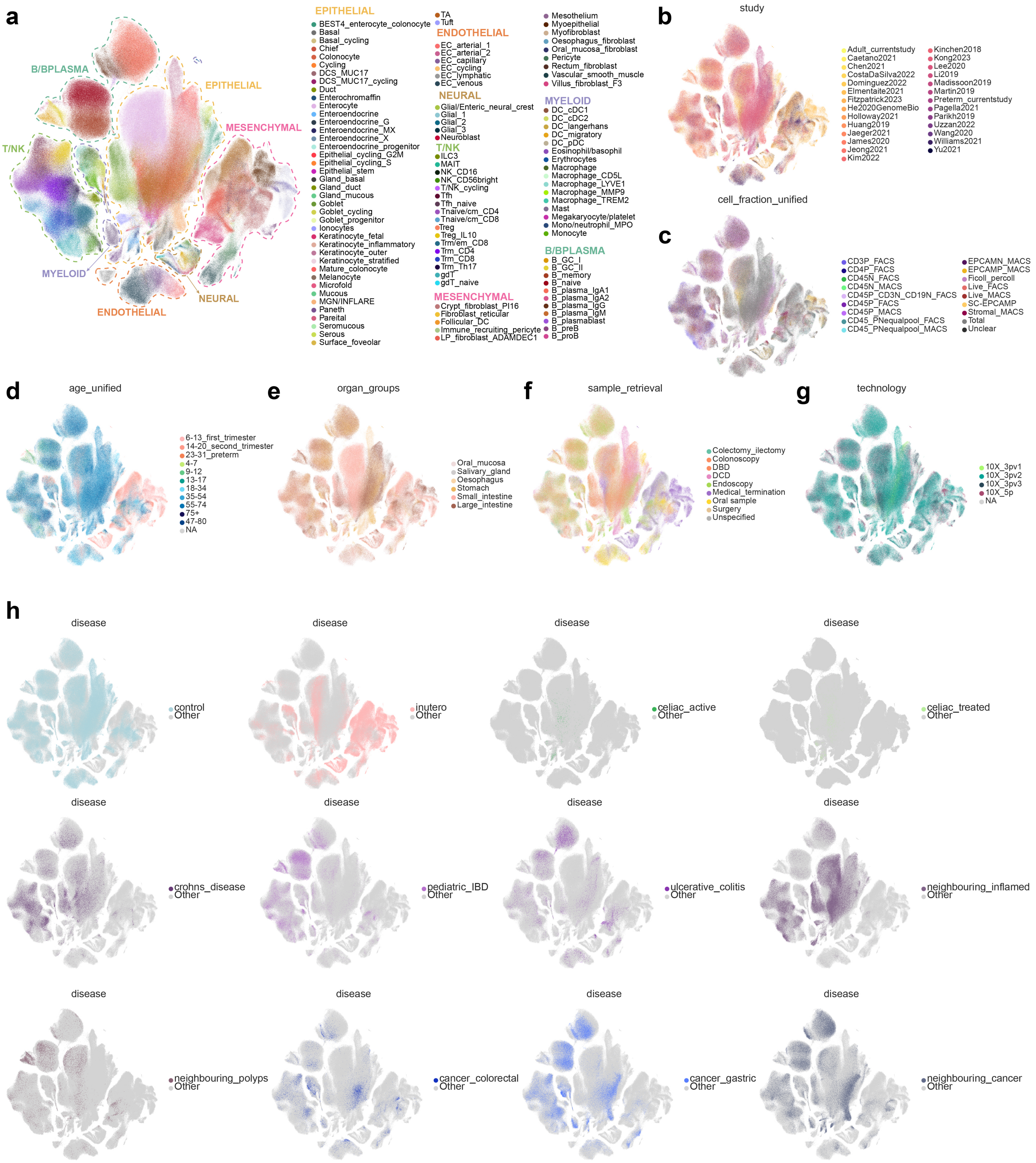

Supplementary Figure 6: Visualisations of the full pan-GI atlas. a-h) UMAP as in Figure 2a coloured by various metadata, a) fine grained annotation (level_3_annot), b) study, c) cell fraction, b) age group, e) GI region, f) sample retrieval method, g) sequencing chemistry/technology, h) disease as in Figure 2a but each condition highlighted individually for easier visualisation.

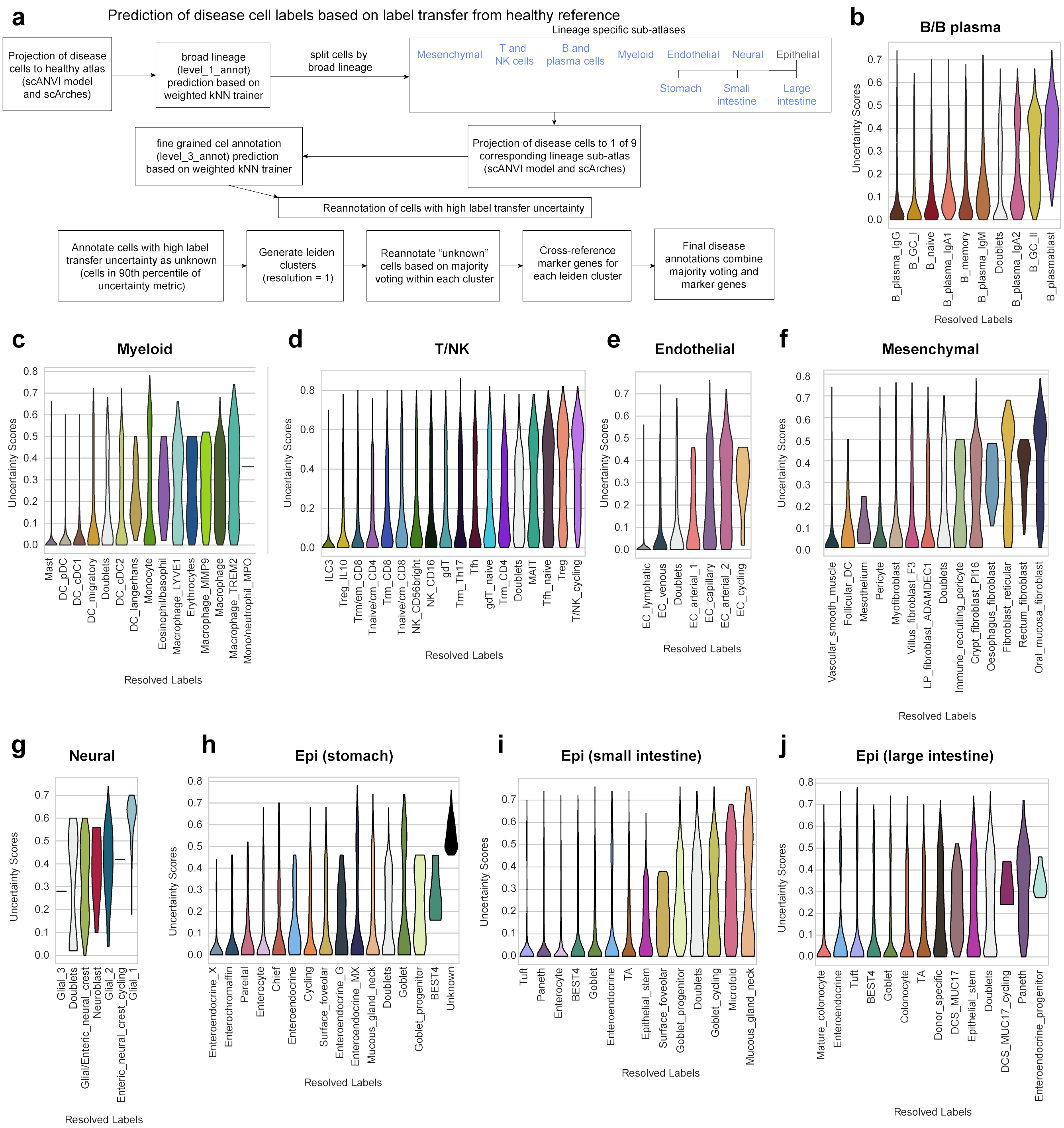

Supplementary Figure 7: a) Flowchart for the annotation of cells from disease samples. As described in Extended data 1, cells from disease samples were added to the atlas by scArches projection using scANVI models. Annotations from the healthy reference were transferred by a weighted kNN classifier, cells with an uncertainty score within the 90th percentile were classified as unknown and reannotated based on majority voting of leiden clusters. All disease annotations were cross-referenced with marker gene expression from literature and through differential gene expression analysis. b-j) Violin plots of label transfer uncertainty per cell type, grouped by lineage for annotations of diseased cells using the approach described in Methods, and summarised in Extended data 1a and Extended data 4. b) B/B plasma (106,472 cells), c) myeloid (18,221 cells), d) T/NK (89,537 cells), e) endothelial (8,121 cells), f) mesenchymal (18,903 cells), g) neural (918 cells), h) epithelial cells from stomach (29,381 cells), i) epithelial cells from small intestine (165,793 cells), j) epithelial cells from large intestine (116,103 cells).

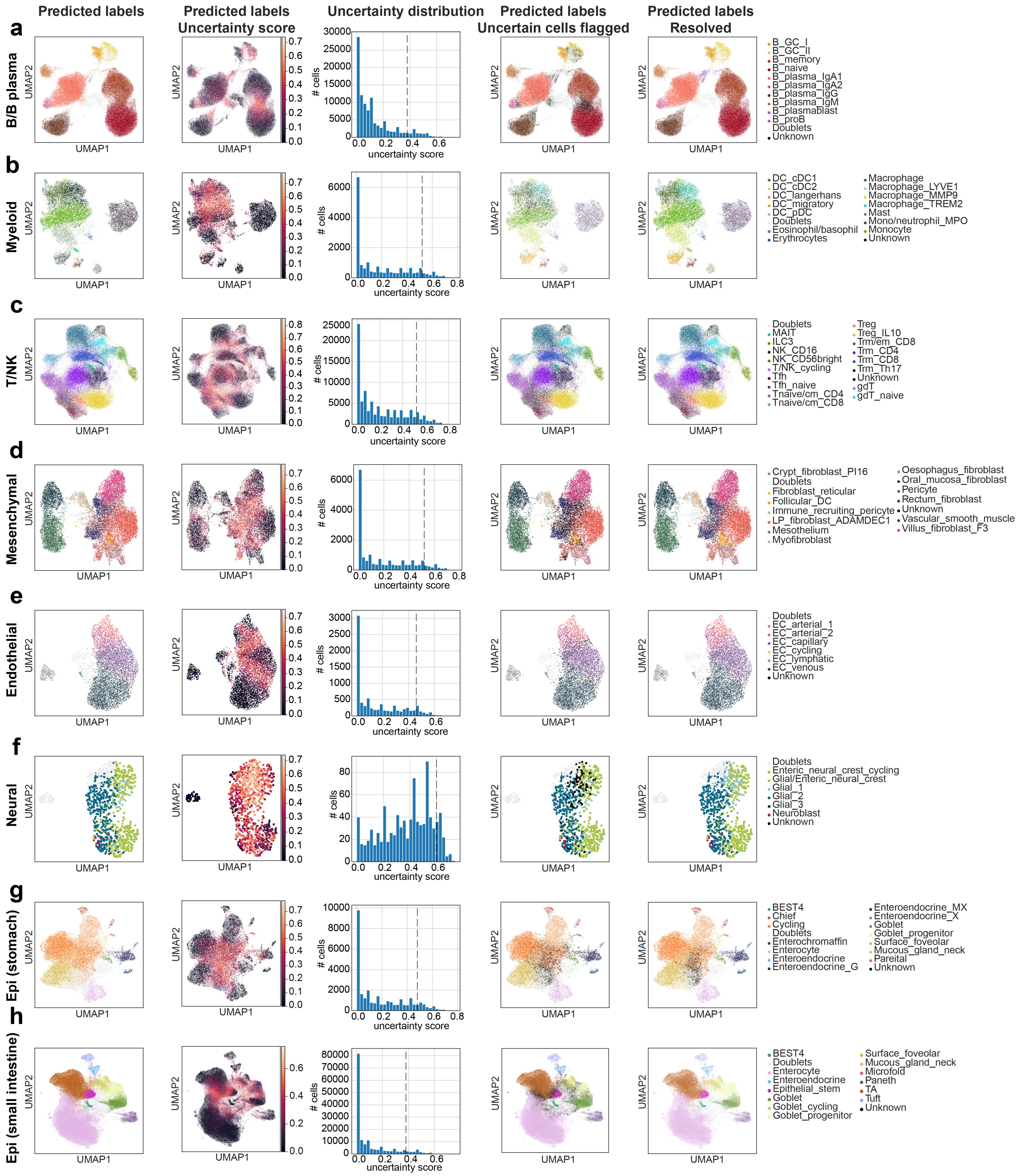

Supplementary Figure 8: Annotation and mapping of diseased cells in the extended atlas with scANVI/scArches transfer learning using the approach outlined in Methods and summarised in Extended data 1a and Supplementary Figure 7a. Each row shows plots by column for predicted labels (weighted kNN trainer), uncertainty score, uncertainty distribution with dotted line at 90th percentile cut off, cells labelled as uncertain based on the uncertainty scores within the 90th percentile and the resolved annotations based on leiden clustering and majority voting/manual assignment. Rows are a) B/B plasma cells, b) myeloid cells, c) T/NK cells, d) mesenchymal cells, e) endothelial cells, f) neural cells, g) epithelial cells from stomach and h) epithelial cells from small intestine. Equivalent plots for epithelial cells from large intestine can be found in Extended data 5.

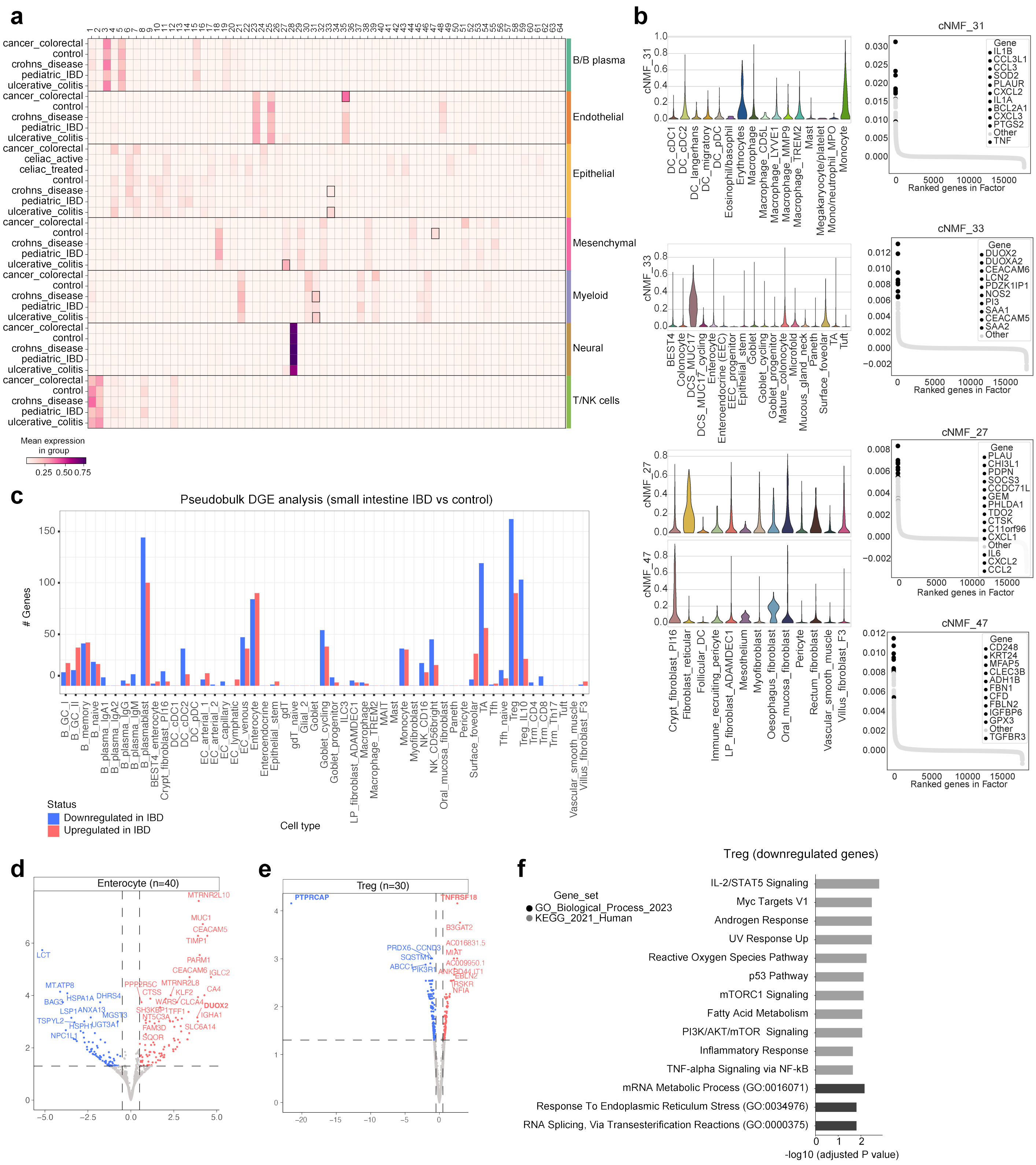

Supplementary Figure 9: Analyses across all lineages comparing health and disease conditions. a) cNMF analysis of cells from health and disease in the small and large intestine (see Methods) with heatmap showing mean expression of scores for genes within 64 factors across disease conditions grouped by broad cell types (level_1_annot). Noteable disease/cell type specific factors are highlighted with a black box. b) Violin plot for gene scores in highlighted factors for relevant lineage along with gene rank plot highlighting top 10 genes and notable highly ranked genes in the corresponding factor. c) Pseudobulk (decoupler) and differential gene expression analysis (DESeq2) across all cell types in small intestine comparing healthy control samples with inflamed IBD samples, plot shows the number of significantly differentially expressed genes ($p < 0.05$, log2FC > 0.5), based on two-sided Wald test with Benjamini and Hochberg correction. d, e) Volcano plot of DEGs in enterocytes (control n = 25, IBD n = 15) (d) and Tregs (control n = 13, IBD n = 17) (e) genes

with positive log2FC are upregulated in IBD. f) Gene set enrichment analysis (GSEA) for genes downregulated in Tregs from IBD (The adjusted p-values have been calculated using wilcoxon rank-sum test).

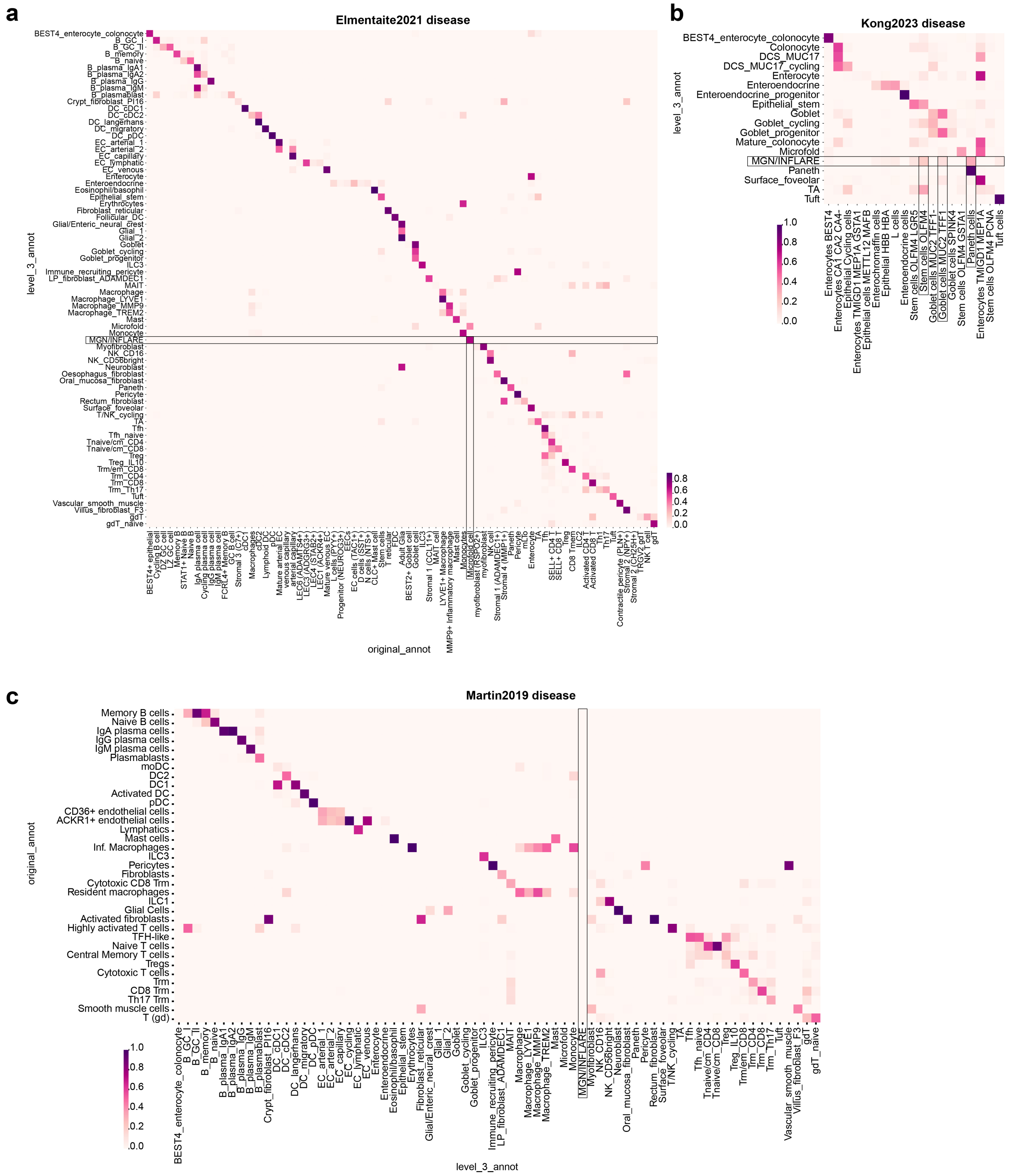

Supplementary Figure 10: Confusion matrix of annotations of cells from disease in the original published studies (with INFLAREs) versus the harmonised annotations from the current atlas. a) Elmentaite et al. 2021, b) Kong et al. 2023, c) Martin et al. 2019.
