# Supplementary material for: Single-cell integration reveals metaplasia in inflammatory gut diseases
Source: Nature. 2024 Nov 20;635(8039):699–707. doi: 10.1038/s41586-024-07571-1 (PMC11578898; doi:10.1038/s41586-024-07571-1)
Supplement: Supplementary file 2 — Reporting Summary [file 41586_2024_7571_MOESM2_ESM.pdf]

nature portfolio

Corresponding author(s): Dr Sarah Teichmann

Last updated by author(s): Apr 26, 2024

Reporting Summary

Nature Portfolio wishes to improve the reproducibility of the work that we publish. This form provides structure for consistency and transparency in reporting. For further information on Nature Portfolio policies, see our Editorial Policies and the Editorial Policy Checklist.

Statistics

For all statistical analyses, confirm that the following items are present in the figure legend, table legend, main text, or Methods section.

n/a	Confirmed	
☐	☒	The exact sample size (*n*) for each experimental group/condition, given as a discrete number and unit of measurement
☐	☒	A statement on whether measurements were taken from distinct samples or whether the same sample was measured repeatedly
☐	☒	The statistical test(s) used AND whether they are one- or two-sided<br>*Only common tests should be described solely by name; describe more complex techniques in the Methods section.*
☐	☒	A description of all covariates tested
☐	☒	A description of any assumptions or corrections, such as tests of normality and adjustment for multiple comparisons
☐	☒	A full description of the statistical parameters including central tendency (e.g. means) or other basic estimates (e.g. regression coefficient) AND variation (e.g. standard deviation) or associated estimates of uncertainty (e.g. confidence intervals)
☐	☒	For null hypothesis testing, the test statistic (e.g. *F*, *t*, *r*) with confidence intervals, effect sizes, degrees of freedom and *P* value noted<br>*Give P values as exact values whenever suitable.*
☒	☐	For Bayesian analysis, information on the choice of priors and Markov chain Monte Carlo settings
☒	☐	For hierarchical and complex designs, identification of the appropriate level for tests and full reporting of outcomes
☐	☒	Estimates of effect sizes (e.g. Cohen's *d*, Pearson's *r*), indicating how they were calculated

Our web collection on statistics for biologists contains articles on many of the points above.

Software and code

Policy information about availability of computer code

Data collection	For public datasets deposited to ArrayExpress, archived paired-end FASTQ files were downloaded from ENA or ArrayExpress. For public datasets deposited to GEO, if the SRA archive did not contain the barcode read, URLs for the submitted 10X BAM files were obtained using srapath v2.11.0. The bam files were then downloaded and converted to fastq files using 10x bamtofastq v1.3.2. If the SRA archive did contain the barcode read, SRA archives were downloaded from the ENA and converted to FASTQ files using fastq-dump v2.11.0. Sample metadata was gathered from the abstracts deposited to GEO or ArrayExpress, and supplementary files from publications.
Data analysis	The following software packages were used, with version number available where applicable:<br>- General: anndata = v0.8.0, numpy = v1.20.1, scipy = v1.6.1, pandas = v1.3.0, scikit-learn = v0.24.1, statsmodels = v0.12.2, python-igraph = v0.8.3, seaborn = v0.11.1, matplotlib = v3.6.3, ggplot2 = v3.4.2<br>- Single cell analysis and processing: STARsolo v1.0, STAR v2.7.9a, CellBender v0.2.0, scanpy = v1.8.0, scVI-tools = v0.16.4, CellChat = v1.1.1, CellPhoneDB = v3, Milopy = v0.0.999, gseapy v1.0.4, cNMF (https://github.com/dylkot/cNMF) = v1.3.4, monocle3 = v1.3.1, BayesPrism = v2.0, TCGAbiolinks = v2.18.0, Harmony-pytorch = v0.1.7 , BBKNN = v1.4.1 , scIB = 1.1.4, Decoupler = v1.5.0, DESeq2 = v1.38.0, GeneOverlap = v0.99.0, LIANA+ = v1.0.4, Palantir = v1.3.1, CellRank = v2.0.1, SingleCellExperiment = v1.12.0.<br>Our newly developed package:<br>scAutoQC (Teichmann sctk package: https://github.com/Teichlab/sctk/blob/master/sctk/_pipeline.py)<br>- Imaging analysis: PathViewer = v3.4.0, QuPath = v0.5, cellpose = v2.2.3, OMERO.web = v5.14.1.

For manuscripts utilizing custom algorithms or software that are central to the research but not yet described in published literature, software must be made available to editors and reviewers. We strongly encourage code deposition in a community repository (e.g. GitHub). See the Nature Portfolio guidelines for submitting code & software for further information.

Data

Policy information about availability of data

All manuscripts must include a data availability statement. This statement should provide the following information, where applicable:

- Accession codes, unique identifiers, or web links for publicly available datasets
- A description of any restrictions on data availability
- For clinical datasets or third party data, please ensure that the statement adheres to our policy

Raw sequencing data for adult samples are available through ArrayExpress with accession number E-MTAB-14050.
Published single cell transcriptomic data accessed and harmonised in the atlas are available under the following accession numbers: Caetano2021 (GSE152042), Chen2021 (GSE188478), CostaDaSilva2022 (GSE180544), Dominguez2022 (E-MTAB-11536), Elmentaite2021 (E-MTAB-9543, E-MTAB-9536, E-MTAB-8901), He202 (GSE159929), Holloway2021 (E-MTAB-9489), Huang2019 (GSE121380), Jaeger2021 (GSE157477), James2020 (E-MTAB-8007, E-MTAB-8474, E-MTAB-8484, E-MTAB-8486), Jeong2021 (GSE167297), Kim2022 (GSE150290), Kinchen2018 (GSE114374), Lee2020 (EGAS00001003779, E-MTAB-8410), Li2019 (GSE122846), Madissoon2019 (PRJEB31843), Martin2019 (GSE134809), Pagella2021 (GSE161267), Parikh2019 (GSE116222), Uzzan2022 (GSE182270), Wang2020 (GSE125970), Williams2021 (GSE164241), Yu2021 (E-MTAB-10187, E-MTAB-10268), Kong (SCP1884).
Published bulk transcriptomic data used for bulk devoncolution are available under the following accession numbers: adult IBD from the Gene Expression Omnibus (GEO) database (GSE111889), LCM tissue from IBD patients and controls (GSE126199), pediatric IBD from the ArrayExpress database (E-MTAB-5464) and the Expression Atlas (E-GEOD-101794), TCGA colon adenocarcinoma using R package TCGAbiolinks and celiac disease data from GEO (GSE131705 and GSE145358).
Imaging data are available for download from the European Bioinformatics Institute (EBI) BioImage Archive with accession number S-BIAD1139. All relevant processed single cell objects and models for use in future projects will be available upon publication, through gutcellatlas.org/pangi.html.

Research involving human participants, their data, or biological material

Policy information about studies with human participants or human data. See also policy information about sex, gender (identity/presentation), and sexual orientation and race, ethnicity and racism.

Reporting on sex and gender	Biological sex is reported for all donors with available information, gender information is not available.<br>Breakdown of sex in the atlas (pulished and unpublished data): male = 123, female = 108<br>Breakdown of sex in tissue sections used for validation: male = 5, female = 12.<br>Sex comparison was not performed.
Reporting on race, ethnicity, or other socially relevant groupings	Self-reported ethnicity information was only reported for 3 out of 271 donors in the atlas. For unpublished data the race, ethnicity or other socially relevant groupings are not reported.
Population characteristics	Available information about other population characteristics is available in the atlas meta data. For key information:<br>Breakdown of ages in the atlas (published and unpublished data): 6-13 weeks old embryo = 16, 14-20 weeks old fetus = 12, 23-31 weeks preterm infants = 4, 4-7 years old = 6, 9-12 years old = 7, 13-17 years old = 5, 18-34 years old = 49, 35-54 years old = 51, 55-74 years old = 68, 75+ = 11, 47-80 = 8.<br>Breakdown of donor diseases in the atlas (published and unpublished data): healthy controls (all ages) = 129, Crohn's disease = 61, gastric or colorectal cancer = 48, ulcerative colitis = 10, pediatric IBD = 10, jeuvenyle polyps = 3, active celiac = 3, treated celiac = 2, mandibular gingiva carcinoma = 1, fistula revision = 1, focal intestinal perforation = 1.<br>Breakdown of ages in the tissue sections used for validation: 13-17 years = 2, 18-34 years = 3, 35-54 years = 6, 55-74 years = 5.<br>Breakdown of donor diseases in the tissue sections used for validation: celiac disease = 2, Crohn's disease = 16, ulcerative colitis = 3.
Recruitment	Most single cell transcriptomics data comes from published studies. For unpublished data, healthy tissue from adult donors was obtained from the Cambridge Biorepository of Translational Medicine (CBTM) from deceased transplant organ donors. For control tissue from preterm infants, patients between 23 and 31 post conception weeks (pcw), with necrotising enterocolitis (NEC), focal intestinal perforation or intestinal fistula (n = 4) were collected at the Neonatal Department of Newcastle upon Tyne Hospitals NHS Foundation Trust with consent and ethical approval as part of the SERVIS study. Adult CD surgical resections were collected from patients in the IBSEN III (Inflammatory Bowel Disease in South Eastern Norway) at Oslo University Hospital, or Hospital Clinic Barcelona and biopsy material was collected from patients undergoing colonoscopy at Addenbrookes Hospital Cambridge. Ulcerative Colitis tissue was also collected from Hospital Clinic Barcelona during colonic resections. Celiac disease tissue was obtained from Oslo University hospital or the Oxford University Hospitals NHS Foundation Trust (OUHFT) celiac disease clinic.
Ethics oversight	Ethical approval references:<br><br>Healthy tissue from adults from CBTM (REC 15/EE/0152 approved by East of England - Cambridge South Research Ethics Committee)<br>Control tissue from preterm infants from Newcastle upon Tyne Hospitals NHS Foundation Trust as part of the SERVIS study (REC 10/H0908/39 approved by North East - Newcastle & North Tyneside 2 Research Ethics Committee)<br>Disease tissue collected at Oslo University hospital (REK 20521/6544, REK 2015/946, and REK 2018/703, Health Region South-East, Norway<br>Disease tissue collected at OUHFT (REC 21/TH/0206, Yorkshire & The Humber - Sheffield Research Ethics Committee)<br>Disease tissue collected at Hospital Clinic Barcelona (HCB/2016/0389, Ethics Committee of Hospital Clinic Barcelona)

Note that full information on the approval of the study protocol must also be provided in the manuscript.

Field-specific reporting

Please select the one below that is the best fit for your research. If you are not sure, read the appropriate sections before making your selection.

☒ Life sciences ☐ Behavioural & social sciences ☐ Ecological, evolutionary & environmental sciences

For a reference copy of the document with all sections, see nature.com/documents/nr-reporting-summary-flat.pdf

Life sciences study design

All studies must disclose on these points even when the disclosure is negative.

Sample size	Data was integrated from a total of 271 donors, across 688 single cell sequencing runs. Total number of transcriptomes analysed was 1,596,203. No Sample size calculation was performed, sample size was dictated by the availability of published datasets, with raw FASTQ files available to run through our QC and atlas building pipeline.
Data exclusions	Approximately 30% of cells/droplets were excluded based on failed QC using our custom method scAutoQC (see methods and extended data 2 for details), a further ~20% were excluded as doublets based on doublet detection methods and manual removal during cell annotation. Samples with less than 10% of cells or less than 100 cells total passing QC were removed from the study due to poor overall quality. These exclusion criteria were set based on logical QC processes common for single cell data analysis to derive high quality data.
Replication	Cells from single cell data come from the studies outlined in the data availability statement. Each cell type is represented from at least 2 donors from at least 2 studies (except myoblast/myocytes which were only found in one study due to biological reasons related to age range and organs sampled). Cell types key to the manuscript conclusions (eg. INFLARE cells), were represented in at least 8 donors from at least 4 independent studies. Key findings from single cell transcriptomics were validated using IHC/smFISH in tissue sections from disease patients (at least n = 2 for validation staining), and generalised in public bulk RNAseq datasets. All attempts at replication were successful.
Randomization	Randomisation was not applicable in the study due to use of publicly available single cell data, and for validation cohorts due to low patient numbers and analysis of a rare cell type.
Blinding	Blinding was not applicable to this study due to use of publicly available single cell data, and for validation cohorts due to low patient numbers and analysis of a rare cell type.

Reporting for specific materials, systems and methods

We require information from authors about some types of materials, experimental systems and methods used in many studies. Here, indicate whether each material, system or method listed is relevant to your study. If you are not sure if a list item applies to your research, read the appropriate section before selecting a response.

Materials & experimental systems

n/a	Involved in the study	
☐	☒	Antibodies
☒	☐	Eukaryotic cell lines
☒	☐	Palaeontology and archaeology
☒	☐	Animals and other organisms
☒	☐	Clinical data
☒	☐	Dual use research of concern
☒	☐	Plants

Methods

n/a	Involved in the study	
☒	☐	ChIP-seq
☒	☐	Flow cytometry
☒	☐	MRI-based neuroimaging

Antibodies

Antibodies used	The following antibodies were used for IHC staining:<br>- anti-human MUC6 clone CLH5 (RA0224-C.1, Scytek, 1:400)<br>- anti-human MUC5AC clone CLH2 (MAB2011, Sigma, 1:100)<br>- anti-human CD3 rabbit polyclonal (A0452, Dako, 1:50)<br>- anti-human CD8 clone 4B11 (MA1-80231, Leica Biosystems, Invitrogen, 1:30)<br>- anti-human CD4 clone SP35 (MA5-16338, Thermo Fisher, 1:30)<br>- anti-human TCR delta clone H-41 (sc-100289, Santa Cruz Biotechnology, 1:100)<br>- anti-human Foxp3 clone 236A/E7 (NBP-43316, Novus Biologicals, 1:1000)<br>- anti-human HLA-DR alpha-chain clone TAL.1B5 (M0746, Dako, 1:200)<br>- anti-human CD68 clone PG-M1 (M0876, Dako, 1:100)<br>- anti-human CD20 clone L26 (M0755, Dako, 1:200)<br>- anti-human TFF2 clone #366508 (RnD, MAB4077, 1;1000)<br>- anti-human TFF3 clone BSB-181 (BioSB BSB-3820-01, 1:1000)<br>- anti-human pan-CK (Ventana, 760-2595, neat)

- anti-mouse HRP (Roche, 5269652001,)

Validation

All antibodies are commercially available and validated by the manufacturers. Datasheets are available at the manufacturer's website. All antibodies were validated by the manufacturers using biological and orthogonal strategies, and previously used in published data (https://www.citeab.com). Each antibody was titrated and validated in single stains, and irrelevant, concentration-matched primary antibodies were used as negative controls.

The link to the protocol, with the validation statement in each vendor website, is provided below for each antibody:
Anti-MUC6 (Scytek): https://www.scytek.com/products/99.12-RA0224-C.1-MUC6-(Mucin-6---Gastric-Mucin)-Clone-CLH5-(Concentrate).asp
Anti-MUC5AC (Sigma-Aldrich) :https://www.sigmaaldrich.com/GB/en/product/mm/mab2011?utm_source=google&utm_medium=cpc&utm_campaign=10193651930&utm_content=101663337573&gclid=CjwKCAjwt-OwBhBnEiwAgwzrUtoU_wp2SrGxoUNLQK91n-cB1odzi8g5QlirBxk0BDhkNwGKTh4HyxoCyDYQAvD_BwE
Anti-CD3 (Dako, Agilent): https://www.agilent.com/en/product/immunohistochemistry/antibodies-controls/primary-antibodies/cd3-%28concentrate%29-76133
Anti-CD8A (Leika, Invitrogen): https://shop.leicabiosystems.com/ihc-ish/ihc-primary-antibodies/pid-cd8
Anti-CD4 (Thermo Fisher): https://www.thermofisher.com/antibody/primary/target/cd4?gclid=CjwKCAjwt-OwBhBnEiwAgwzrUuUcG7aO5YAKTlvDU55Pa3k29HuYCxxVtmg_x-gfHyUpLGcWUgZtVBoCjecQAvD_BwE&ef_id=CjwKCAjwt-OwBhBnEiwAgwzrUuUcG7aO5YAKTlvDU55Pa3k29HuYCxxVtmg_x-gfHyUpLGcWUgZtVBoCjecQAvD_BwE:G:s&s_kwcid=AL!3652!3!593537744328!p!!g!!ebioscience%20cd4!2081760689!80608360681&cid=bid_pca_aup_r01_co_cp1359_pjt0000_bid00000_0se_gaw_bt_pur_con&gad_source=1
Anti-TCR delta: (Santa Cruz Antibodies) https://www.scbt.com/p/tcr-delta-antibody-h-41
Anti-Foxp3 (Novus Biologicals): https://www.novusbio.com/primary-antibodies/foxp3?gad_source=1&gclid=CjwKCAjwt-OwBhBnEiwAgwzrUnjzTVa9kG4-USgw_7DGCXZ7Gn_giAhWA8aObxRjctI7FsVw9Omo3hoC_scQAvD_BwE&gclsrc=aw.ds
Anti-Pan Keratin (Roche): https://elabdoc-prod.roche.com/eLD/web/pi/en/products/RTD00068
Anti-HLA-DR (Dako, Agilent): https://www.agilent.com/cs/library/packageinsert/public/SSM0746CEEFG_01.pdf
Anti-CD68 (Dako, Agilent): https://www.agilent.com/en/product/immunohistochemistry/antibodies-controls/primary-antibodies/cd68-%28concentrate%29-76550
Anti-CD20 (Dako, Agilent): https://www.agilent.com/en/product/immunohistochemistry/antibodies-controls/primary-antibodies/cd20cy-%28concentrate%29-76520
Anti-TFF2 (R&D): https://www.rndsystems.com/products/human-tff2-antibody-366508_mab4077
Anti-TFF3 (BioSB): https://www.biosb.com/biosb-products/tff3-antibody-mmab-bsb-181
